# Geochemical properties of stream sediments and suspended particulate matter and determination of geochemical background values for the Alpine River Mura

**DOI:** 10.1007/s10653-026-03331-4

**Published:** 2026-07-18

**Authors:** Barbara Čeplak, Ulrike Moser, Johanna Irrgeher, Martin Šala, Gorazd Žibret

**Affiliations:** 1 Geological Survey of Slovenia, Dimičeva Ulica 14, 1000 Ljubljana, Slovenia; 2 Chair of General and Analytical Chemistry, Franz-Josef-Strasse 18, 8700 Leoben, Austria; 3 National Institute of Chemistry, Hajdrihova Ulica 19, 1000 Ljubljana, Slovenia

**Keywords:** Stream sediments, Suspended particulate matter, Cluster analysis, Contaminant monitoring

## Abstract

Suspended particulate matter (SPM) and stream sediments (STS) from 66 locations (main channel and tributaries) were geochemically investigated in the Alpine transboundary river Mura to provide an integrated view of the long-term accumulation of pollutants and the present-day pollution status of the catchment, including the distribution of YREE (yttrium and rare earth elements) which are regarded as emerging pollutants due to use of modern technologies. Cluster analysis identified 4 geochemical associations in SPM for the low water regime: (1) Cu, Cd, Bi, Pb, Mn, Ni and Cr; (2) Ca, Sr; (3) U, Fe, Co, Rb, YREE, V, Ti, Mg and (4) Ba, Ga, Al and Li. Six groups were identified for STS samples: (1) YREE, U, Mn, Fe, Ti; (2) As, V, Ga, Al; (3) Ca, Sr, Na; (4) Cu, Ni, Co, Cr, Mg; (5) Ag, Bi, Pb, Cd, Zr and (6) Ba, Zn, Rb and Li. The study concluded that the identified geochemical groups reflect the geological composition of the catchment area as well as anthropogenic impacts, notably mining, steel industry, and urbanisation. The spatial distribution of YREE was identified as an indicator of sediment provenance. Eu and Ce anomalies were observed in SPM, reflecting the local geology, while the Gd anomaly may indicate anthropogenic inputs. The study concludes that STS primarily reflect the geogenic distribution of elements, while SPM is a more sensitive indicators of anthropogenic activities for the Alpine fluvial environment. Study also provides upper background and threshold values for Mura River catchment that may serve as reference values in future environmental studies.

**Graphic Abstract:**

**Figure:**
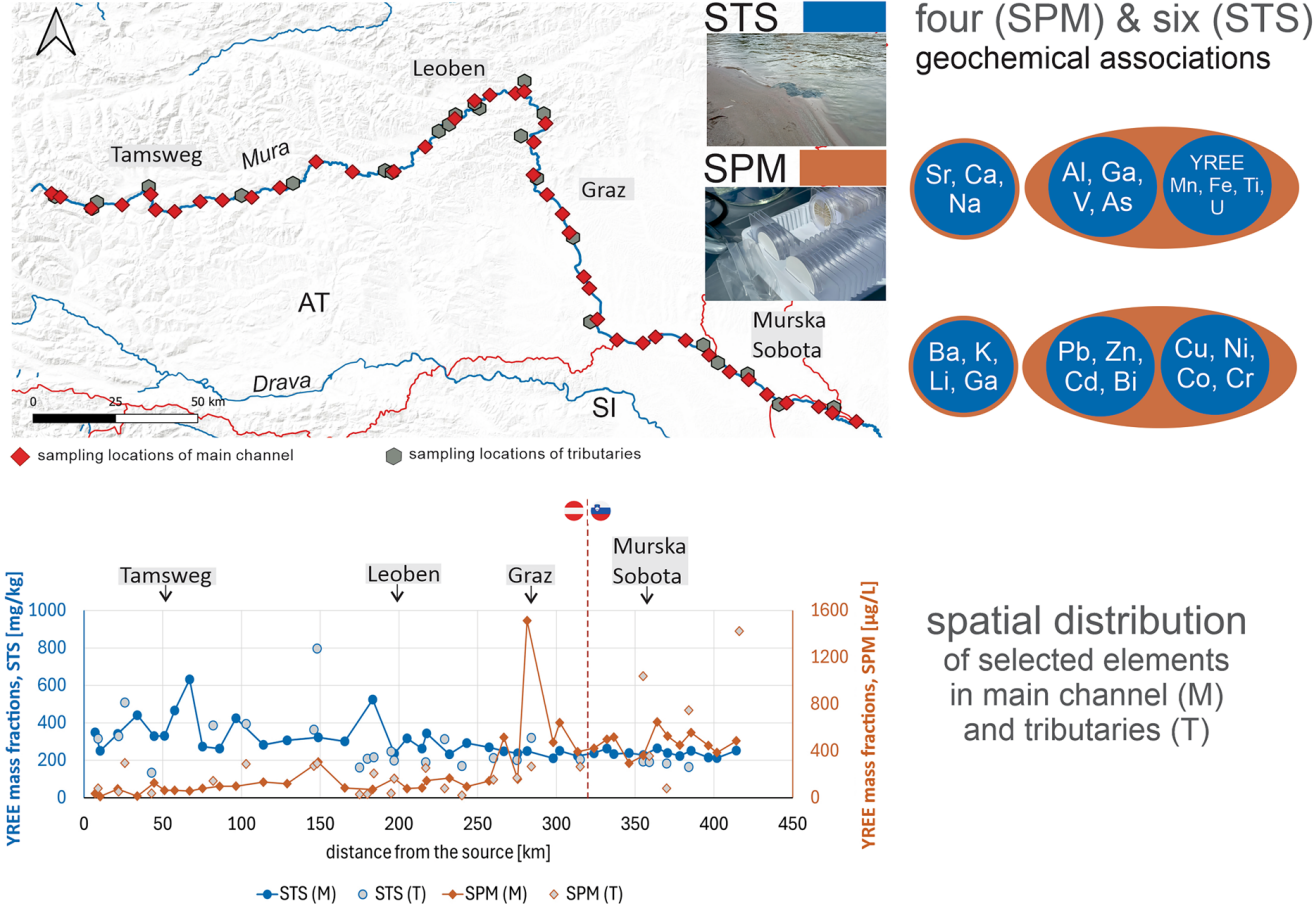

**Supplementary Information:**

The online version contains supplementary material available at 10.1007/s10653-026-03331-4.

## Introduction

Rivers represent an important continental pathway for transporting products of physical and chemical weathering (Meybeck, 1976). The elemental and mineralogical composition of alluvial sediments primarily reflects the composition of source material and post-erosional alteration by weathering processes and also reveals past and present anthropogenic influences (Klein et al., 2021, 2022b; Natali & Bianchini, 2017; Zimmermann et al., 2020).

In order to distinguish between natural and anthropogenic inputs to fluvial systems, it is essential to define the geochemical background data. This topic has been addressed by several studies. Within the GEMAS project, geochemical background concentrations and threshold values for European agricultural soils were established (Reimann et al., 2018). At the regional scale, Gosar et al. (2019) determined corresponding background and threshold values for Slovenian topsoil. Comparable efforts have been carried out in Austria, where Pfleiderer et al. (2012) defined threshold values for minor elements in soils from the Vienna area. Beyond soils, several studies have also emphasized the importance of determining background and threshold values in sediments worldwide, as demonstrated by the work of Klein et al. (2022a), Salomão et al. (2020), Kralj et al. (2016) and Barrio-Parra et al. (2025).

Rare earth elements (REEs) are immobile and remain relatively inactive in the processes of low-grade metamorphosis, weathering and hydrothermal alteration (McLennan, 1989; Wu et al., 2013), which makes them an excellent indicator for sediment provenance studies. Rock weathering is the primary source of REEs and other major and trace elements; however, with the growth of the high-tech industry in the recent decades, REEs have become emerging pollutants. They are widely used in various fields, including medicine, green technologies (e.g. solar panels, electric vehicles), smartphones, computers, and agricultural activities (Cobelo-García et al., 2015; da Silva et al., 2018). Although REEs are present in our everyday lives, the influence of REEs on the environment, their behaviour and their potential impact on health of living organisms are not well understood (Tepe et al., 2014).

REE anomalies can occasionally be found in rocks and sediments, including Eu, Ce, La, Sm, Gd and Tm (Barrat et al., 2023). Eu and Ce anomalies are particularly important, and both elements occur in nature in two valence states (Eu^2+^ and Eu^3+^, Ce^3+^ and Ce^4+^). During weathering or magmatic fractionation, Ce and Eu can be separated from their neighbouring REE, thus causing specific elemental depletion or enrichment in normalized REE patterns (Barrat et al., 2023). On the other hand, La, Sm, Gd and Tm anomalies are independent of the influence of valence. The Tm anomaly is a legacy of the building blocks from which our planet was formed (Dauphas & Pourmand, 2015), while the origin of the other anomalies is most likely due to anthropogenic effects (Klein et al., 2022b; Kulaksiz & Bau, 2011). In this study, we focused on Eu, Ce and Gd anomalies. There are several different equations for calculating Eu, Ce and Gd anomalies, which are mainly divided into linear and geometric ones. Linear equations use constants of adjacent pairs, which can lead to significantly different results depending on whether chondritic or shale values are used for normalization, while the geometric methods are based on the assumption that the concentrations of neighbouring elements are nearly constant. In this study, geometric methods were used, which provide a more reliable determination of anomalies, regardless of the type of normalization (Lawrence et al., 2006).

The elemental composition of suspended particulate matter (SPM) has been treated as a useful tool for monitoring contamination in aquatic environments (Kaiser et al., 1989; Loring & Rantala, 1992; Masson et al., 2018; Schubert et al., 2012), where grain size, sampling methodology and sample preparation procedures may significantly influence the obtained mass fractions of individual elements. Unlike stream and/or bed sediments, SPM is more homogenous material, containing little or no proportion of bed material (Masson et al., 2018). Furthermore, inorganic particles are more likely to be incorporated into SPM than to remain dissolved (Violintzis et al., 2009). Several studies have addressed the anthropogenic incorporation of REE (e.g., Klein et al., 2022a, 2022b; Liu & Han, 2021; Soroaga et al., 2022). These studies have mostly focused on Gd anomalies in wastewater, which are related to the application of Gd-containing contrast agents in medical diagnoses. Although these Gd complexes have been shown to be very stable in water, it is suspected that Gd precipitation may occur, leading to increased Gd contents even in alluvial sediments (Klein et al., 2022b; Liu & Han, 2021; Soroaga et al., 2022). While the content of anthropogenically introduced REE in water is relatively well known, their level in sediments is generally poorly studied (i.e. Altomare et al., 2020; da Silva et al., 2018).

Elements of anthropogenic origin may enter an aquatic system in dissolved form (Goldstone et al., 1990). However, depending on their properties, they may remain dissolved or be absorbed by suspended particles through the formation of organic or inorganic complexes and then deposited in sediments (Cánovas et al., 2012). SPM tend to absorb the dissolved heavy metals due to their reactivity and large surface area (Li et al., 2018) and biochemical processes (Conway & John, 2014). Previous studies focused mostly on heavy metal absorption including Cu, Pb, Zn, Cd, Cr (Viet, 2018; Zeng et al., 2020), while such studies did not address technology critical elements (i.e. REE) (Culicov, et al., 2021).

This study builds on the work of Čeplak et al. (2025), which focused on the geochemical composition of stream sediments (STS) and alluvial sediments in the Mura River. The study found that the geochemical composition of the stream (main channel and tributaries) and floodplain sediments of the Mura River–based on two fractions (< 0.063 mm and 0.063–0.125 mm)–is mainly influenced by natural processes. Therefore, the main objective of this study is to (i) extended the research to the SPM of the same river system (Table S1). Special attention is placed to the (ii) YREE (yttrium and rare earth elements) distribution in STS and SPM samples, because these elements can be regarded as a good tracer of sediment origins (Bau et al., 2018; Saha et al., 2023; Verma et al., 2022) and at the same time they are regarded as emerging pollutants because of the use of modern technologies (Cobelo-García et al., 2015; Hedayatzadeh et al., 2025). Identification of (iii) geochemical associations in SPM and STS will help to identify sources of elements and their processes in this fluvial system. (iv) Establishing geochemical background values for such emerging pollutants will enable assessing anthropogenic fluxes in the future.

## Materials and methods

### Study area and sampling locations

The Mura River is a transboundary Alpine watercourse that forms part of the Black Sea (Danube River) catchment and flows through Austria, Slovenia, Hungary and Croatia, where it flows into the Drava. The Mura River is 466 km long and its catchment area covers 14,371 km^2^ (Brilly et al., 2011). The river has an ice-snow regime, with a maximum flow at the beginning of summer, i.e., in May and June, while the minimum flow is from October to March (Hrvatin, 1998). In the upper part, the Mura is a mountainous river, while in the middle part, it transforms into a typical lowland river. In the lower part it becomes a meandering river.

A total of 65 samples of STS and 107 samples of SPM from the main channel (labelled as M-00 to M-40) and the tributaries (labelled as T-01 to T-25) were collected throughout the Mura River catchment (Table S1). STS samples of the main channel were collected in August 2022, while SPM samples were obtained in February 2023, during a low water regime, in August 2022, during average water regime and during November 2023, when STS and SPM samples from tributaries were collected. Table 1 shows water discharges at the Gornja Radgona gauging station, located in the lower part of the catchment near Slovenia-Austria border, measured during sampling campaigns.

**Table 1:** Water discharges at the gauging station Gornja Radgona

Date	August 2022 discharge (m^3^/s)	Sampling date	February 2023 discharge (m^3^/s)	Sampling date	November 2023 discharge (m^3^/s)
1. 8.	100	6. 2	78	21. 11	166
2. 8.	95	7. 2	76	22. 11	148
3. 8.	90	8. 2	69	23. 11	142
4. 8.	86	9. 2	67		
5. 8.	84				
Average 1991–2020	167		95		148

**Fig. 1:**
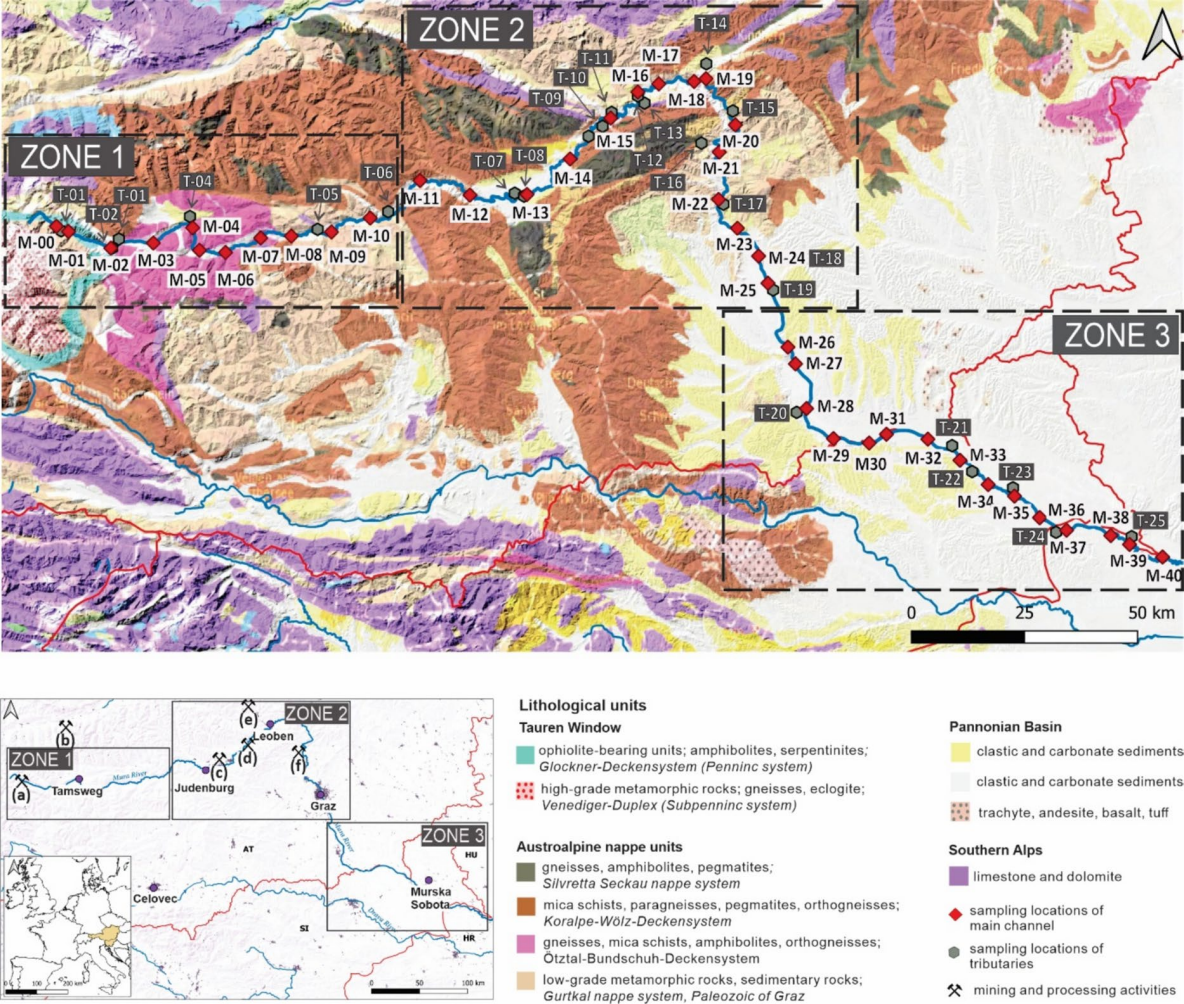
Simplified geological map of the study area in Zones 1, 2 and 3 with sampling locations (M–main channel, T–tributaries) and major mining areas: (**a**) As–Au–Ag mine Rotgülden, (**b**) Ag–Ni mine Obertal, (**c**) Cu–Au mine Flatschach, (**d**) Kraubath area, (**e**) Fe mine Erzberg and (**f**) Pb–Zn mine Arzwaldgraben (Source: OneGeology GISEurope Bedrock and Structural geology, SRTM 90 m DEM) (summarized by Čeplak et al. (2025))

The Mura catchment area is lithologically heterogeneous and can be divided into three different zones: Zone 1, Zone 2 and Zone 3 (Fig. 1) described in Čeplak et al. (2025).

### Analytical procedures

Different analytical procedures were used for STS and SPM and are described separately in the next chapters.

#### Stream sediments (STS)

The material for STS (approximately 2 kg) was collected with a plastic spatula from at least 5 different locations within the convex river band in a prelabelled plastic bag and then dried in an oven at 303 K (30 °C) to a constant weight. Plants, rocks and other debris were removed from the samples, aggregates were gently crushed in a ceramic mortar. Approx. 5 g pulps of < 0.063 mm fraction were sent to the external laboratory for determination of elemental composition (Bureau Veritas laboratories, Vancouver, Canada, MA250 analytical package). A detailed description of the sample preparation of STS and the analytical procedure is provided in Čeplak et al. (2025).

Accuracy was validated by 31 measurements of 8 reference materials (SdAR–L2–RM, SdAR–H1–RM, SdAR–M2, OU–6, OREAS45F, OREAS45H, OREAS501D and OREAS25A–4A; Table S2). Measurements below the detection limit were estimated with 50% of the corresponding lower limit of detection (LOD) (USEPA, 2000). Elements were excluded from further statistical and other analyses if the element mass fractions were below the LOD in more than one-third of the sample set.

#### Suspended particulate matter (SPM)

Water samples were collected 10 cm below the surface using acid-cleaned polyethylene (PE) bottles, which were rinsed three times with river water before collecting the water sample containing SPM. SPM was obtained using pressurized filtration through a 0.22 µm filter with a vacuum pump (LABOPORT® N 816.3 KT.18). The filters were changed every 250 ml to 1 L of filtrated water, depending on the amount of suspended material (the filter supposed to be completely covered with SPM). After filtration, the filters were stored in petri dishes. Determination of elemental composition of dried filters containing SPM was performed at the National Institute of Chemistry in Ljubljana. The SPM containing filters were digested at 473 (200 ℃) K in Teflon beakers using 2 ml HNO_3_ and 6 ml H_2_O_2_. The cooled beaker walls were subsequently washed with MQ to obtain a total volume of 25 ml. The resulting solutions were diluted 40 times before analysing. The mass fractions of chemical elements were determined with inductively coupled plasma–mass spectrometry (ICP-MS).

The results are expressed in µg/L, instead of mg/kg, because of the challenges associated with accurate weighting of water-saturated materials in the field (before and after sampling), which would introduce a high risk of significant weighing uncertainty and associated errors. To avoid this, we chose not to rely on gravimetric determination. Additionally, reporting concentrations in mg/L rather than mg/kg allows us not only to describe the elemental composition of suspended matter, but also to directly relate the measured elemental concentrations to the volume of water filtered. This approach provides information on the amount of suspended matter per litre of water, which we believe is an important and complementary indicator, rather than focusing solely on the composition of the particulate material itself.

HF was not used for digestion because preliminary validation tests were performed using certified reference (SRM) material. The recoveries for all analysed elements were in very good agreement with certified values, demonstrating that the digestion procedure was complete. In the case of fine particulate matter, even materials that are typically more resistant to digestion tend to be more easily digested due to the very small particle size and high surface area, further supporting the adequacy of the applied method. Validation was complemented using internal laboratory quality control solutions, blanks and standard reference material. Clean filters were also digested and analysed, and obtained average values were subtracted from the values obtained on filters containing SPM. Average elemental levels of blank filters (3 measurements) are presented in supplementary material, Table S3. Seven repeated analyses of 4 reference materials (SdAR-L2, SdAR-M2, SdAR-H1, OU-6) were performed. Measurements below the LOD were estimated at 50% of the corresponding detection limit (USEPA, 2000). Elements with measurements below the LOD in more than one-third of samples were excluded from further data processing.

### Data processing

Statistical analyses were done using the software Statistica 13 (TIBCO Software Inc., 2020). Spearman correlation coefficients were calculated on raw data, while cluster analysis was done on logarithmically transformed datasets (ln(x + 1); x means elemental mass fractions in the sample expressed in mg/kg). 26 elements or a group of elements in STS and SPM samples (low water regime only) were considered in the statistical analysis. Distribution maps of selected elements of each elemental group in the STS and SPM samples were created using Golden Software Surfer version 22.3.185. The abundances of YREEs in a sample were normalized to European shales (ES) (Bau et al., 2018).

YREE levels were normalized to remove the so-called Oddo-Harkins effect (i.e., elements with even atomic numbers are more abundant) (Bau et al., 2018). Eu, Ce and Gd anomalies were calculated afterwards using normalized values according to Eqs. 1, 2 and 3. Eu anomaly was calculated based on Lawrence et al. (2006) whereas Nd and Sm are more commonly used for calculating the Eu anomaly because Gd could be influenced by anthropogenic activities

1 $$Eu/Eu^{*} = \frac{{Eu_{N} }}{{Sm_{N} *\left( {\frac{{Sm_{N} }}{{Nd_{N} }}} \right)^{1} /2^{{}} }}$$

Ce anomaly is most often based on elements that do not show anomalies in nature and have relatively constant concentrations, i.e., Pr and Nd (Eq. 2) (Lawrence et al., 2006; Wu et al., 2019). La may be introduced into the environment by anthropogenic activities and is therefore not recommended to be used for calculating Ce anomaly.

2 $$Ce/Ce^{*} = \frac{{Ce_{N} }}{{\Pr_{N}^{2} /Nd_{N} }}$$

The logarithmic equation was used to calculate the Gd anomaly (Eq. 3) in a similar way it was used in the Rhine River case (Kulaksiz & Bau, 2013). This method is usually applied to non-tropical rivers, as tropical rivers usually have a medium REE enrichment, and are slightly acidic and enriched in organic colloids.

3 $$Gd/Gd^{*} = \frac{{Gd_{N} }}{{10^{{\frac{{4 \times \log Eu_{N } - \log Nd_{N} }}{3}}} }}$$

The positive and negative Eu, Ce and Gd anomalies are determined with values more or less than 1 considering a 5% error (i.e., 0.95–1.05).

### Estimation of background values

The estimation of elemental background values for the Mura River began with a visual assessment of elemental concentration distributions along the river course to identify elements exhibiting spatial patterns indicative of anthropogenic influence. In cases where pronounced increases in elemental concentrations were observed in association with urban areas, major mining sites, or industrial centres, only data from Zone 1 were used for background value determination, where no significant anthropogenic influences are expected. For other elements background values were determined using a percentile-based method as proposed by Reimann et al. (2018). In this study the 75th and 90th percentiles were presented. Threshold concentrations were additionally calculated using two statistical approaches: the MD2MAD method (Median + 2 × Median Absolute Deviation; Eq. 4) and the Tukey Inner Fence (TIF; Eq. 5) methods by given formulas.

4 $$\begin{gathered} MD2MAD = 10^{{median (\log_{10} \left( {X_{i} )} \right) + 2 \times MAD \left( {\log_{10} \left( {X_{i} } \right)} \right)}} \hfill \\ MAD = 1.48 median\left( {X_{i} - median \left( {X_{i} } \right)} \right) \hfill \\ \end{gathered}$$

X_i_ represents the levels of the respective element.

5 $$TIF = {\mathrm{Q}}3 + 1.5 {\mathrm{IQR}}$$

Q3 is the 3rd quartile of the dataset while IQR is the interquartile range (Q3-Q1).

The TIF method was applied to log-transformed or otherwise normalized data (Reimann et al., 2008).

## Results

**Fig. 2:**
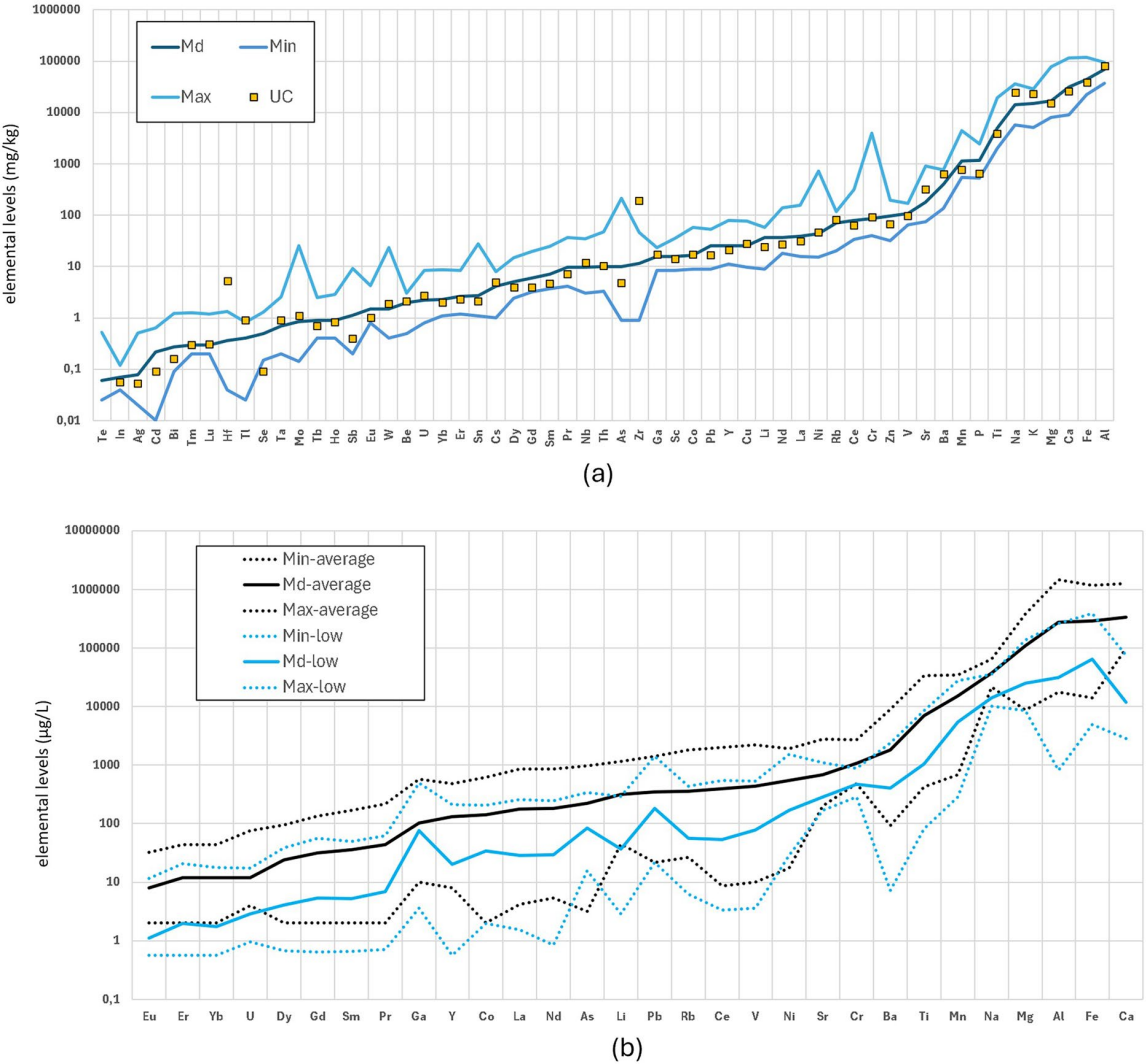
(**a**) Abundance of elements in STS (Min–minimum, Md–median and Max–maximum values) and comparison with elemental abundance in Earth’s upper crust (UC, Rudnick & Gao, 2013), and (**b**) abundance of elements in SPM (Min–minimum, Md–median and Max–maximum values) in average and low water regime

The coordinates of exact sampling locations (Table S1), descriptive statistical parameters for STS (Table S2), SPM (Table S3), values for elemental levels in STS (Table S4) and SPM (Tables S5 and S6) are presented in the supplementary material. Figure 2 presents the minimum, maximum and median elemental levels for STS (all sampling points) and SPM (main channel only). It is evident that the most abundant elements in both STS and SPM are Al, Fe, Ca, Mg and Na.

### Geochemical associations

**Fig. 3:**
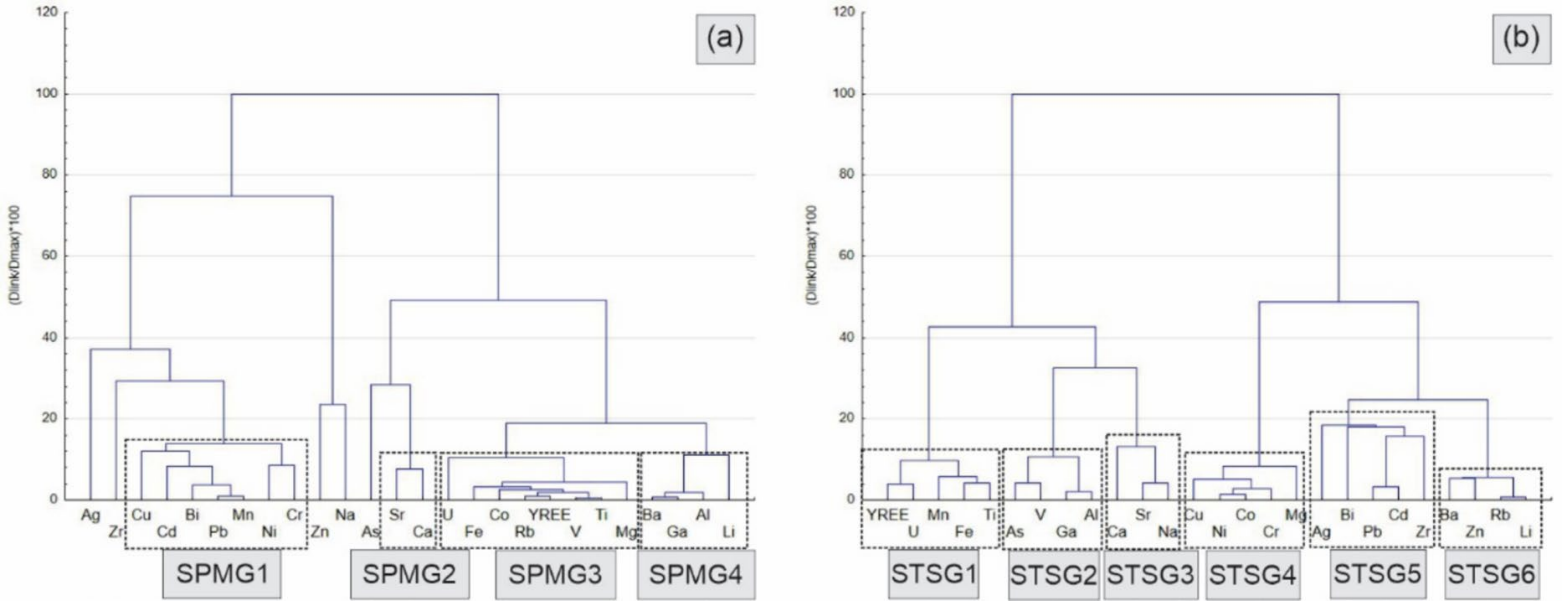
Cluster analysis dendrogram (Ward’s method, 1-Pearson r; n = 41, 26 elements or groups of elements) for (**a**) SPM and (**b**) STS

Four groups (G) of elements were identified for SPM (low water regime only): SPMG1 (Bi, Cd, Cr, Cu, Mn, Pb); SPMG2 (Ca, Sr); SPMG3 (Co, Fe, Mg, Rb, Ti, V, YREE); SPMG4 (Al, Ba, Ga, K, Li), and six groups for STS: STSG1 (YREE, U, Fe, Mn, Ti); STSG2 (As, Al, Ga, V); STSG3 (Ca, Na, Sr); STSG4 (Co, Cr, Cu, Mg, Ni); STSG5 (Ag, Bi, Cd, Pb, Zr); STSG6 (Ba, K, Li, Rb) (Fig. 3).

**Fig. 4:**
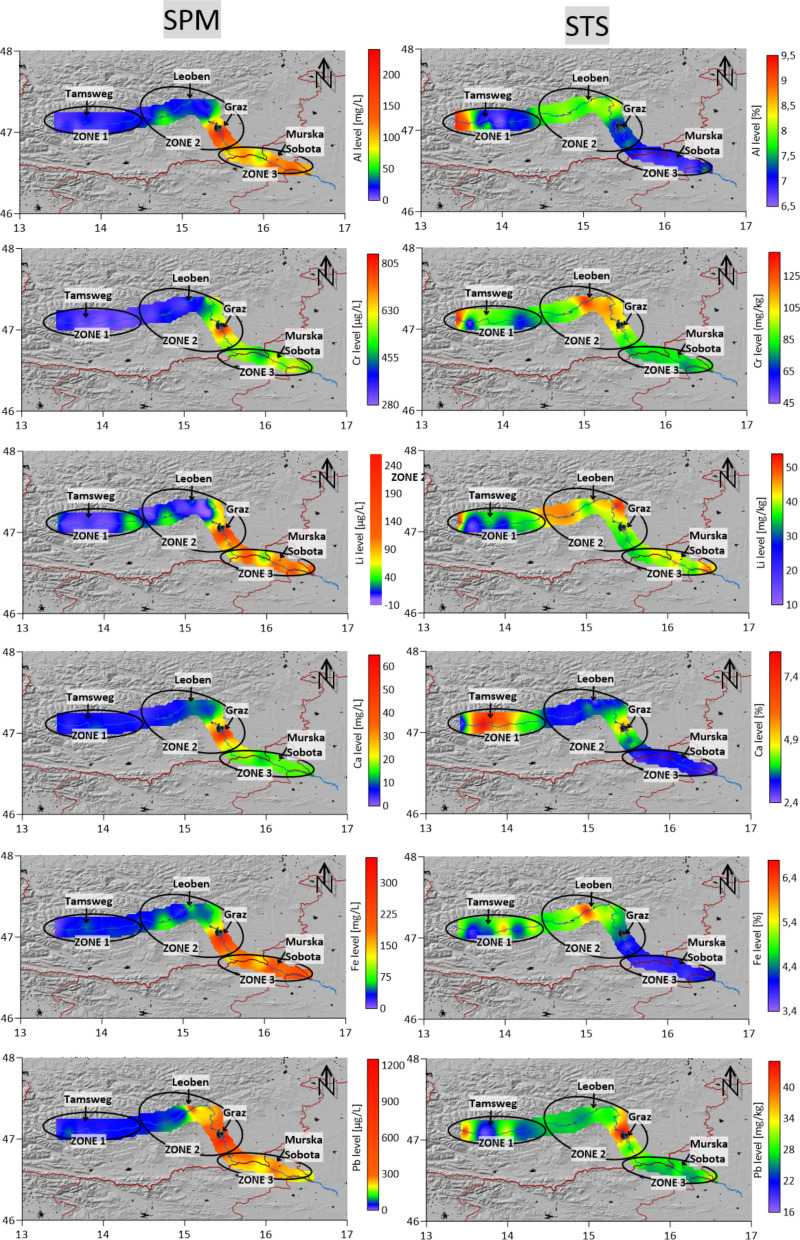
Spatial distribution of Al, Cr, Li, Ca, Fe and Pb mass fractions in stream sediment (STS) and suspended particulate matter (SPM) samples from the main channel. Circles represent zones (in accordance with Fig. 1)

The spatial distributions of the most representative elements of each group in STS and SPM (Al, Ca, Cr, Fe, Li and Pb) are presented in Fig. 4. The Al content in STS is generally higher in Zones 1 and 2 compared to Zone 3, while for SPM it is just the opposite–the higher levels were measured in the lower part of the river. Cr levels in SPM were the highest around Graz, with locally increased areas also in the Zone 3, while in STS the highest Cr levels were measured in the Zone 2, near Leoben. Li levels in STS are the highest in the area around Leoben, while in SPM they are the highest in Zone 3. Ca levels in STS are the highest in the upper part, while in SPM the highest Ca levels were measured around Graz. Fe levels in STS are the highest in Zone 2, while for SMP the highest Fe levels were measured in the lower part. Pb levels in SPM are especially high near Graz and sites below the city, while for STS the highest levels were measured in the area between Leoben and Graz.

**Fig. 5:**
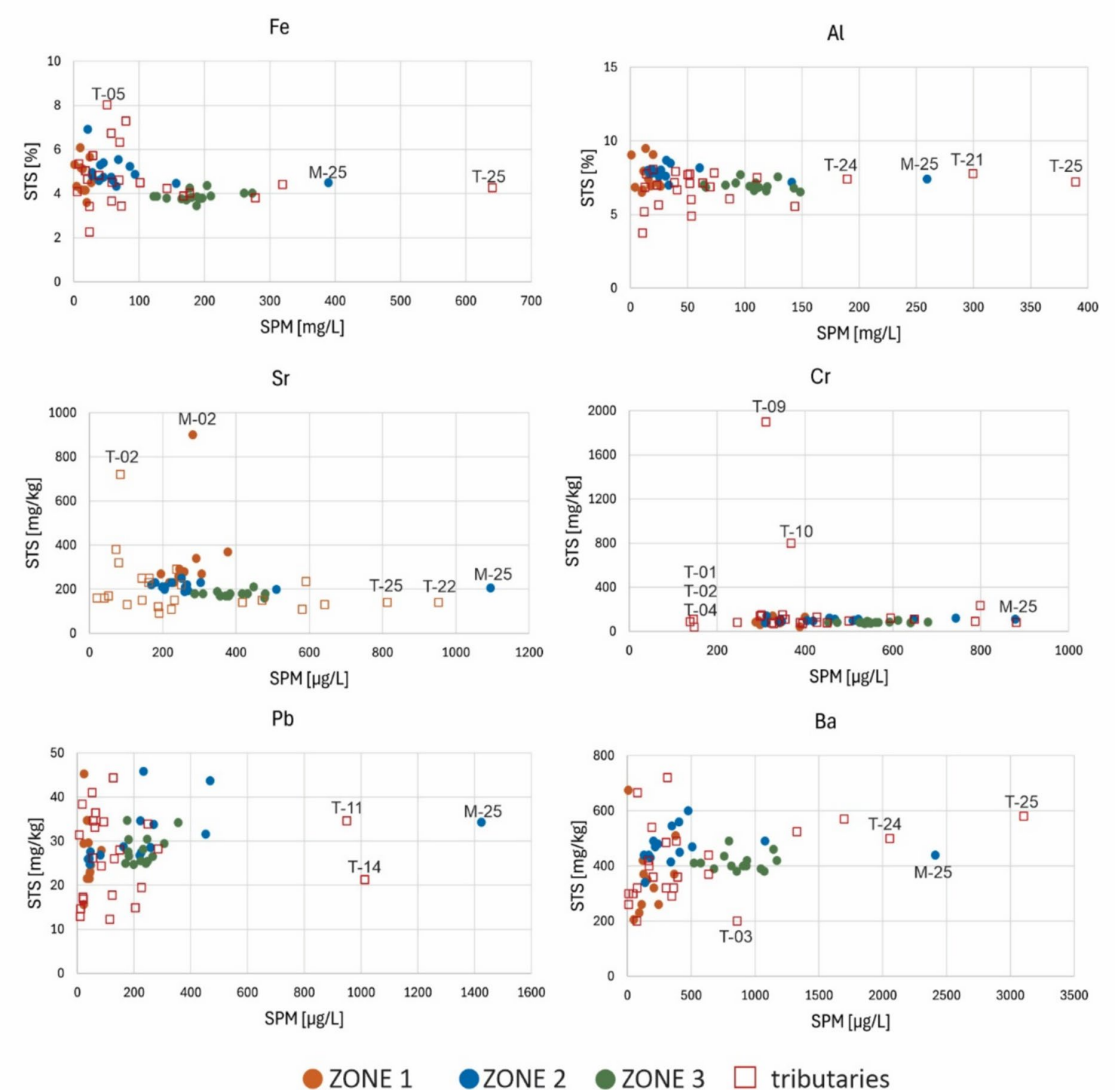
Scaling of elemental mass fractions in stream sediment (STS) and suspended particulate matter (SPM) for Fe, Al, Sr, Cr, Pb and Ba in upper (Zone 1), middle (Zone 2) and lower (Zone 3) parts of the catchment in the main channel (M) and tributaries (T)

Figure 5 shows scaling of elemental levels between STS and SPM in Zones 1 to 3 to detect possible patterns in the data. According to the plots it was not possible to establish clear relationships. However, it is evident that the points representing tributary samples are commonly scattered outside the main channel’s point cloud. This is especially evident in the cases of Fe, Al, Sr, Cr, Pb and Ba. The data also indicates that points representing measurements from the main channel in specific zones are generally clustered.

### Spatial distribution of rare earth elements

**Fig. 6:**
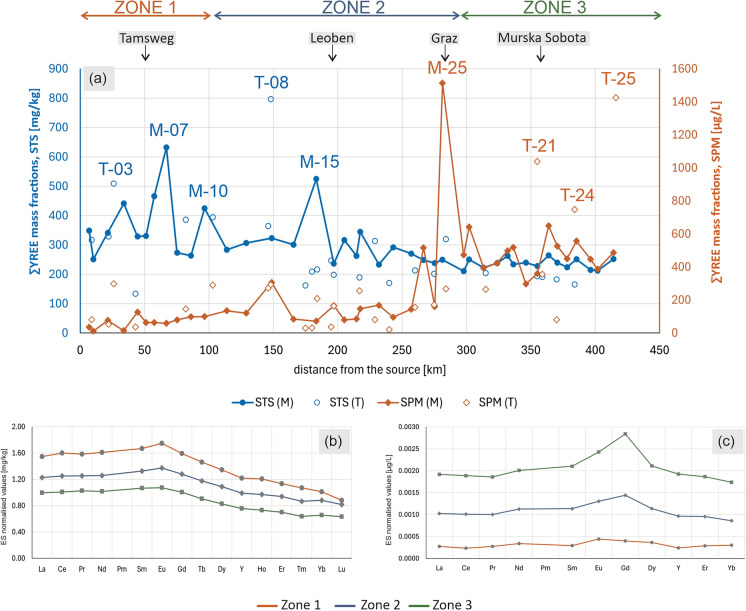
(**a**) Distribution of ∑YREE in STS and SPM according to the distance from the source (SPM samples do not include Tb, Ho, Tm and Lu values, as measurements in more than one-third of analyses are below the detection limits); M–main channel, T–tributaries and European shale (ES) YREE normalized values (expressed as average) in Zone 1, Zone 2 and Zone 3 in (**b**) STS and (**c**) SPM samples

**Fig. 7:**
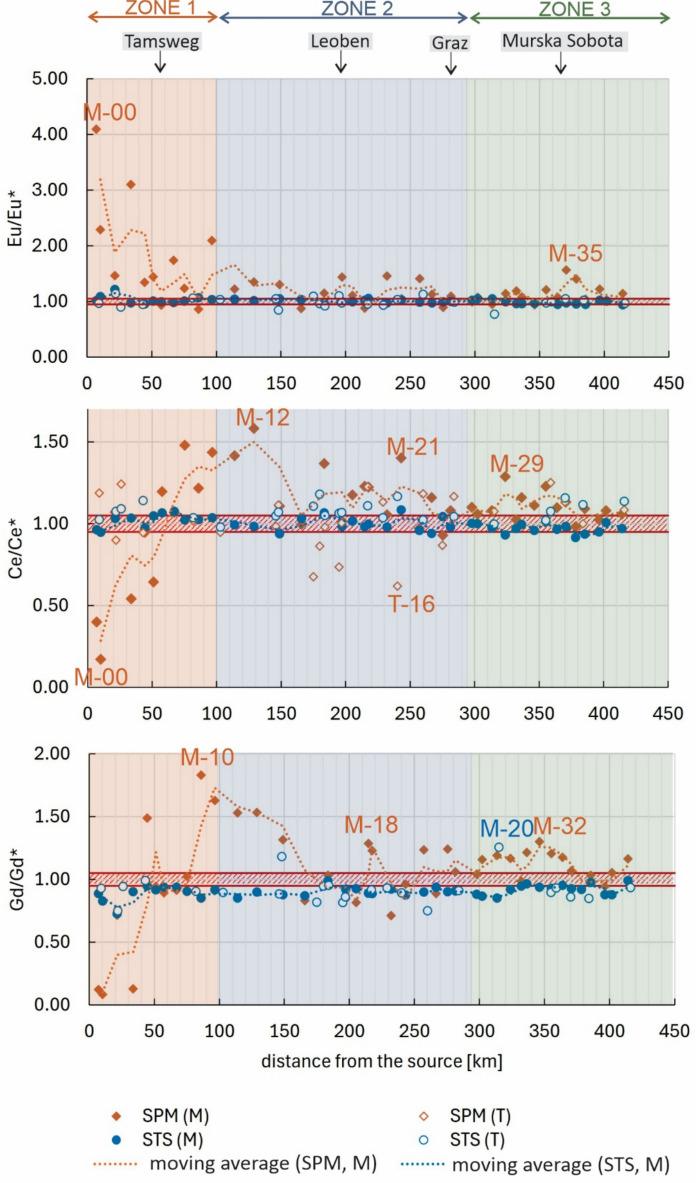
Eu, Ce and Gd anomalies (Eqs. 1, 2 and 3) for SPM (low water regime only) and STS; M–main channel, T–tributaries; the positive and negative Eu, Ce and Gd anomalies are determined by the values of higher or lower than 1 considering 5% error (red belt)

Mass fractions of the ∑YREEs in analysed samples (main channel and tributaries) ranged from 0.01 to 1.5 mg/l for SPM (low water regime) and from 133 to 789 mg/kg for STS. The YREE dataset, obtained from STS, includes the lanthanides from La to Lu and Y. Due to low concentrations of YREEs in SPM, which were near or below detection limits, the following elements were not included in calculations: Tb, Ho, Tm and Lu in the case of main channel, and Tb, Ho, Tm, Eu and Lu in the case of tributaries. Figure 6 presents YREE levels for STS and SPM in main channel and tributaries, and normalized values according to European shales for Zones 1, 2 and 3. Higher YREE levels are observed in the upper part of the river, comparing to the lower part for STS, while the opposite trend was observed for SPM. Figure 7 presents spatial distribution of Eu, Ce and Gd anomalies, while calculated values for Eu, Ce and Gd anomalies are presented in the supplementary material (Tables S4, S5 and S6). Eu anomalies for STS and SPM in the main channel are scattered around the value of 1, with the exception for Eu anomaly values for SPM in the uppermost part (Zone 1), where positive Eu anomaly was detected. The similar trend is observed for Ce, where anomaly values for STS are scattered around 1. Strong negative Ce anomaly was detected in the uppermost part in SPM, which then sharply increases and reach peak at the point M-12. Values of Ce anomaly then drops, but are still generally higher than corresponding values for STS. The same pattern as for Ce is observed also for Gd in STS and SPM.

### Determination of natural geochemical background

Based on the obtained dataset, the following natural geochemical background levels for STS of the Mura River can be suggested, as shown in the Table 2. In cases where element enrichment corresponds to potential anthropogenic emission centres (i.e., urban zones, industry, mines) percentile values have been calculated using data from Zone 1 only, area with less anthropogenic sources. The suggested background values have been established through careful observation of distribution patterns and by selecting criteria that best represent the natural background values.

**Table 2:** Geochemical background values for elements in STS (total levels) calculated according to 4 methods, and suggested background value by the expert. *—percentile values have been calculated for Zone 1 only

El	Unit	Threshold value		Percentile levels		suggested natural geochemical background value
		TIF	MD2MAD	75th	90th	
Ag	mg/kg	0.18	0.13	0.12	0.16	0.16
Al	%	9.9	8.0	8.0	8.5	8.0
As	mg/kg	20	15	12	15	12
Ba	mg/kg	641	509	461	512	461
Be	mg/kg	2.0	2.0	2.0	3.0	2.0
Bi	mg/kg	0.56	0.43	0.37	0.44	0.37
Ca	%	6.9	4.2	4.2	5.5	5.5
Cd	mg/kg	0.43	0.30	0.28	0.30	0.28
Ce	mg/kg	183	123	116	162	123
Co	mg/kg	27	21	18	21	18
Cr	mg/kg	149	104	100	120	100
Cs	mg/kg	6.7	5.5	4.9	5.7	4.9
Cu	mg/kg	55	41	33	41	33
Dy	mg/kg	11	8.2	6.9	8.6	6.9
Er	mg/kg	5.8	4.1	3.5	4.4	4.1
Eu	mg/kg	3.5	2.5	2.2	3.0	2.2
Fe	%	7.1	5.5	4.9	5.4	4.9
Ga	mg/kg	23	20	18	19	18
Gd	mg/kg	14	9.6	8.8	12	8.8
Hf*	mg/kg	2.0	0.78	0.25	0.33	0.25
Ho	mg/kg	1.8	1.5	1.2	1.6	1.2
In	mg/kg	0.10	0.09	0.08	0.09	0.08
K	%	2.3	1.9	1.7	2.0	1.7
La	mg/kg	87	62	58	79	62
Li	mg/kg	65	51	44	47	44
Lu	mg/kg	0.62	0.30	0.40	0.50	0.40
Mg	%	3.2	2.5	2.2	2.4	2.2
Mn	mg/kg	1870	1440	1240	1380	1240
Mo*	mg/kg	4.9	3.1	0.90	1.0	0.90
Na	%	2.0	1.7	1.6	1.7	1.6
Nb	mg/kg	21	16	14	16	14
Nd	mg/kg	82	56	53	74	56
Ni	mg/kg	93	69	57	68	57
P	%	0.26	0.19	0.16	0.19	0.16
Pb	mg/kg	40	34	30	35	30
Pr	mg/kg	21	15	14	19	15
Rb	mg/kg	103	93	85	96	85
Sb	mg/kg	2.0	1.5	1.4	1.6	1.4
Sc	mg/kg	34	26	20	25	20
Se*	mg/kg	1.7	0.8	0.45	0.55	0.45
Sm	mg/kg	16	11	10	13	11
Sn*	mg/kg	6.7	4.5	2.4	2.7	2.4
Sr	mg/kg	343	269	232	280	232
Ta	mg/kg	1.7	1.0	1.0	1.1	1.0
Tb	mg/kg	1.8	1.4	1.2	1.6	1.2
Te	mg/kg	0.13	0.10	0.07	0.11	0.07
Th	mg/kg	23	16	15	19	16
Ti	%	0.76	0.62	0.59	0.76	0.76
Tl	mg/kg	0.64	0.52	0.47	0.57	0.47
Tm	mg/kg	1.1	0.63	0.50	0.60	0.50
U	mg/kg	4.2	3.1	2.9	3.3	2.90
V	mg/kg	175	141	124	130	124
W*	mg/kg	11	4.4	2.3	2.6	2.3
Y	mg/kg	50	38	33	43	33
Yb	mg/kg	4.4	3.5	2.9	3.8	3.8
Zn*	mg/kg	155	129	95	108	95
Zr*	mg/kg	63	27	7.8	10	7.8

## Discussion

The results of STS and SPM analysis reveal that most elements fall within the average abundance in Earth’s crust as established by Rudnick and Gao (2013). Deviations were found for Zr and Hf, where significantly lower values were measured for SPM, which was likely the result of only partial digestion of zircon minerals (Fig. 2). This finding has already been explained in Čeplak et al. (2025). Contrary, Se values in STS are significantly higher, and the levels of P are also slightly elevated. This is likely due to the presence of organic matter in STS. Additionally, the increased levels of As can be attributed to natural enrichment in the uppermost part of the catchment area, where historic As mining occurred in Rotgülden (Fig. 1). The relative abundance of Sb in STS, compared to that of the upper Earth’s crust, reflects a regional depletion of Sb in stream sediments (Salminen et al., 2005). Relative abundance of main elements in SPM shows that most elements are for a factor of 10 more abundant in high water regime compared to low water regime, which is in line with expectations, since during high water more particles are transported in suspension, contributing to higher measurements. The results of this study align with previous research, which also reported higher element levels under high water regimes compared to low water regimes (Covelli et al., 2007; da Costa-Júnior et al., 2025; Horowitz, 2008; Vidmar et al., 2017). Interestingly, the maximum abundances of certain elements in SPM in low water regime is comparable to those in high water regime, for example Ga, Pb, Ni, Mn. Notably, Pb, Ni and Mn are commonly recognised as anthropogenically induced elements, and the emissions from the steel and ironworking industries (Miler & Gosar, 2015), which are present in the area, may be contributing to these findings. However, more detailed studies are needed to confirm anthropogenic links to these increased values. In high water regime, the dilution effect hinders the anthropogenic emission, while in low water regime the effect can be better observed. This is why further data processing considering SPM has been done only on the dataset from the low water regime.

Dragun et al. (2025) measured sediment composition in the lowest part of the Mura River in Croatia and found enrichments with Mo (1.4 mg/kg), W (6.51 mg/kg), Sn (4.52 mg/kg), Zn (139 mg/kg), Mn (0.19%), Ca (2.51%), Na (0.28%) and Fe (3.98%). These values are more comparable to median values for sediments established in this study (0.84, 1.5, 2.72, 95.5 mg/kg, 0.11, 3.1, 1.4 and 4.3% respectively), while maximum measured elemental levels in stream sediments are much higher in this study (25.4, 23.3, 27.8, 198 mg/kg and 0.44, 11.7, 3.6 and 12% respectively). The results of this study can also be compared to the results from the research of Šajn et al. (2011), who studied soils developed on sediments of the neighbouring Drava River. The Drava River flows in a similar geological environment as Mura, however, Drava sediments in the Pannonian basin are heavily polluted due to historic operations of large Pb–Zn mines in its catchment (i.e. Mežica mines). It is clear that the maximum established levels of Pb, Zn and Cd are 20-fold higher than those in Mura (3300 compared to 198 mg/kg for Zn; 1200 and 53.7 for Pb and 17 and 0.64 mg/kg for Cd), while medians for Zn, Pb and Cd are around twofold higher in the case of Drava (140 and 95.5; 53 and 25.3; 0.5 and 0.22 mg/kg respectively). Contrary, medians for major lithoforming elements are relatively comparable for both rivers, as for example for Al (6.8% in the case of Drava and 7.0% in the case of Mura), Fe (3.8 and 4.3%), K (1.6 and 1.5%), Na (1.1 and 1.4%), Ca (3.4 and 3.1%) etc. This data clearly illustrates the rate of pollution of Drava sediments compared to Mura.

Comparison can be made with the nearby Sava River sediments (Žibret & Gosar, 2017), which flows through the geotectonic area of Southern Alps, composed predominantly of carbonates, in comparison to Mura River, where catchment is located in geotectonic units of Tauern window, Eastern Alps and Pannonian basin. This is reflected in the composition of STS. Median Ca levels for Sava in the fraction < 0.063 mm is 8.01%, while the corresponding value for Mura is much lower, 3.1%. The same is evident for Mg (2.87% for Sava and 1.7% for Mura respectively). Contrary, the Mura River flows in the area composed of crystalline basements and oldest nappes, containing much higher amount of silicate minerals, which is reflected, for instance, in differences in median Al levels (0.705% for Sava and 6,9% for Mura), Fe (2.0 and 4.3%), K (0,06 and 1,5%), Na (0,01 and 1,4%) or Ti (0,003 and 0,49%). It is clear that the geological composition of the catchment plays a crucial role in the composition of STS.

The geochemical associations (6 associations) identified in the STS by cluster analysis are similar to those identified by factor analysis in the previous study by Čeplak et al. (2025), while 4 geochemical associations were identified in the SPM in this study. SPMG1 (Bi, Cd, Cr, Cu, Mn, Ni, Pb), STSG4 (Cu, Ni, Co, Cr, Mg) and STSG5 (Ag, Bi, Pb, Cd, Zr), contains potentially toxic metals, which are commonly linked to anthropogenic emissions. Cu along with As can be linked to polymetallic mineralisation in the Flatschach area (Raith et al., 2015) located in Zone 1 and the still visible impacts of its historic exploitation. The Cr spatial distribution in STS shows an anomaly around Leoben (Fig. 4), likely due to the occurrence of ultramafic rocks in the Kraubath area (Fig. 1), the quarry operation in Chromwerk and potentially also because of emissions from the metal industry. The highest levels of Cr have been found in the right tributaries, T-09 and T-10, which are discharging quarrying sites (Fig. 5). These levels are much greater than those recorded in any of the other measurement locations in this study. However, further studies are needed to precisely pinpoint the sources of Cr in the area. In the case of SPM, the highest Cr levels were measured downstream from Graz (Fig. 4). The spatial distribution of Pb in STS and SPM (Fig. 4) shows that the highest levels are measured upstream from Graz, which could be related to impacts of historic mining activities at Pb–Zn-Ba deposits in the vicinity of Graz (Arzwaldgraben as part of Paleozoic of Graz; Fig. 1) (Geringer et al., 2022; Weber, 1983). Elevated Pb mass fractions in SPM are mainly found downstream from larger urban areas and at sites below cities, suggesting the impact of urbanisation, and is in agreement with the study of Dendievel et al. (2022).

SPMG2 (Sr, Ca) and STSG3 (Ca, Na, Sr) reflects natural processes, related to weathering of carbonate rocks that are the most abundant in the uppermost part of the catchment (Fig. 1). The highest Ca levels in STS were measured in Zone 1 (Fig. 4), especially at point M-03. The study of Moser et al. (2025) also identified high Sr concentrations in river water in the area, indicating a carbonate origin, as Sr is commonly enriched in carbonate rocks and evaporites (Turekian & Kulp, 1956). Elevated Ca mass fractions in SPM samples, as well as slight increase in STS, are further observed in Graz and its surroundings (Fig. 4), where carbonates from Palaeozoic of Graz are exposed.

The composition of SPMG3 (U, Fe, Co, Rb, YREE, V, Ti, Mg; Fig. 3) do not offer clear and uniform explanation. This group contains elements, which are typical for emissions from ironworking and steel industry and ferrous ore mining, which is abundant in the area (Fe, Co, V, Ti and Mg). This is consistent with the results of the study of Reismann (2022), which indicated that Fe, Mn and Ti could be associated with the geochemical properties of iron deposits situated in the catchment i.e. Erzberg, Murau, Obdach and Judenburg, and consequently to the presence of Ti-, Fe- and Mn- bearing minerals such as ilmenite, rutile, titanite and Fe–Mn oxide/hydroxides (Čeplak et al., 2025). On the other hand, U, Rb and YREE could be geochemically linked to felsic and ultra felsic rocks, which are abundant especially in Zone 1, but can also be found in Zone 2. SPMG3 is analogous to geochemical group STSG1 (YREE, U, Mn, Fe, Ti; Fig. 3). Interestingly, the spatial distribution of Fe, which is a typical element of both groups, reveals that the highest levels of Fe in STS are around Leoben and generally in Zone 2, where ironworking, Fe mining and steel industry are prevalent. Conversely, the highest levels of Fe in SPM were found in Zone 3. The most likely explanation for this is that larger particles found in STS are deposited closer to the source. Over time these larger particles decompose due to effects of water, resulting in the formation of smaller and colloidal particles. These particles are more easily transported downstream and are more abundant in the lower part of the river.

SPMG4 contains Ba, Ga, Al and Li and can be analogous to STSG2 (As, V, Ga, Al) and STSG6 (Ba, Zn, Rb, Li). This geochemical group in SPM is likely associated with the weathering of phyllosilicate minerals, especially those from the mica group (i.e. lepidolite, phlogopite), which are known for their high abundance of Al and Li, as well as other elements (cations), that can be incorporated between alumosilicate layers (i.e. Ba, Ga, Rb).

One observation of this study is that certain elements (for example Al, Li, Ca, Fe, Fig. 4) in STS are more abundant in the upper part of the river, while the same elements in the SPM are more abundant in the lower part of the river, especially below Graz, where the Mura River reach Pannonian basin and the water energy drops significantly. The possible explanation is that larger geogenic particles are deposited closer to the source in STS, where they gradually decompose into smaller secondary mineral and colloidal particles, while soluble ions are leached away by water. Such secondary and colloidal particles remain in the suspension and are more abundant in the SPM in the lower part of the river. However, when STS particles reach lowlands, they are already geochemically altered, so levels of, for example, Al, Li, Ca or Fe are consequently low. Another finding of this study is that the highest levels of various elements were measured in tributaries and are significantly different than measurements from the main channel. This applies to STS and SPM, particularly for Fe, Al, Sr, Cr and Ba (Fig. 5). The simplest explanation for this finding is that tributaries carry a much “stronger geochemical signal” compared to the main channel. This effect is commonly utilised in geochemical exploration, where elevated levels of natural ore tracers are often found in active stream sediments from smaller tributaries (1st or 2nd order; Demetriades et al., (2022)). In contrast, the main channel contains more sediments and has a higher water flow, which causes it to act as an “averaging agent”, which is useful for studying elemental mobility and particle-water interactions.

Particular attention in this study was placed on YREE distribution as they are regarded as emerging pollutants at a global level. Several patterns emerge from the obtained data. The YREE levels in the STS vary significantly in the upper part of the river, remaining more constant in the lower part, while the highest variability is observed in the tributaries. The highest level of YREE in STS was observed at tributary T-08, which can be sufficiently explained by the geology. The observed trend is just the opposite for the SPM, where low levels are observed in the upper part, with increased trend downstream. The highest levels in SPM are observed just downstream of Graz, which is the main technological, urban, and industrial centre of the study area. Additionally, increased levels of YREEs have also been observed in some tributaries downstream of Graz, i.e., at T-21 and T-25. Future studies are needed to precisely assess the underlying reason for these increased levels.

Some elements tend to have higher levels in STS in the upper part, while their content in SPM is higher in the lower part. This is also valid for the YREE (Fig. 6). Light YREEs are more abundant in STS (Fig. 6b), and the Gd peak is observed in the lower part of the river in SPM (Fig. 6c). Higher YREE values in STS in the upper part can be associated with lithology, because pegmatites and other alkali rocks (Schmid et al., 2013), which are commonly enriched with YREE-bearing minerals, are present in Zones 1 and 2. Allanite, monazite and xenotime minerals are important YREE carriers, but other minerals such as plagioclases (Eu), apatite (Ce), ilmenite (Y), zircon (Y), spessartine (Y), galena (Y), etc. (Randive et al., 2014), can also be enriched with YREE. Generally, higher levels of LREE indicate the presence of allanite and monazite, while uniform levels of HREE levels suggest potential sources from xenotime, titanite, and garnet minerals (Garzanti et al., 2011; Randive et al., 2014). The presence of xenotime and monazite group of minerals in STS of the Mura River was confirmed by SEM/EDS observations in previous study (Čeplak et al., 2025), therefore mineralogical composition alone cannot sufficiently explain relatively higher abundance of LREEs over HREEs. Contrary, the solubility of HREE is higher than that of LREE (Liu & Byrne, 1998), which corresponds well with the observation from this study, which shows that LREEs in STS are more abundant than HREEs (Fig. 6), especially in Zone 3. This supports the interpretation that physically weathered mineral grains in STS undergo gradual physical and chemical alteration, which leads to slow release of secondary minerals containing a higher proportion of HREEs compared to LREEs, resulting in higher abundance of LREEs in STS. Another potential sources of YREE in the SPM in the lower part of the river can be attributed to the use of phosphorus containing fertilizers, which can contain high levels of YREEs (Liu & Han, 2021; Soroaga et al., 2022), or discharges from municipal water treatment plants (Klein et al., 2022b). Exceptionally high levels of YREEs in SPM in some tributaries (i.e. T-21, T-24 and T-25) can indicate that point sources of YREEs in SPM in Zone 3 are present.

The study by Martin et al. (2021) demonstrated, that light YREE are more likely dissolved in natural river water, compared to heavy YREE. Additionally, dissolved anthropogenic Eu and Gd were detected in the solution in low water flow, compared to suspended particles or in high water flow. These findings are not completely in line with the findings of this study, which indicates potential anthropogenic Gd anomaly in suspended particulate matter in low water regime. The presence of a Gd peak in SPM is likely of anthropogenic origin. This is suggested by the fact that there is no such peak in Zone 1, where no potential strong anthropogenic fluxes are expected. In contrast this peak is visible in Zone 2 and is even more clearly expressed in the lowest part of the river in Zone 3, where the strongest anthropogenic signals are anticipated. Anthropogenic Gd have been detected in rivers (Hissler et al., 2016; Kraemer et al., 2024) and seawater (Kraemer et al., 2024; Moreira et al., 2025; Zocher et al., 2025) in the past, while this study suggests, that anthropogenic Gd can also be detected in SPM. A potential source of anthropogenic Gd might be related to municipal wastewater (Kulaksiz & Bau, 2011; Tepe et al., 2014), because Gd anomaly in SPM is much better expressed in the lower part of the river, compared to the upper parts (Fig. 6).

Observations of Eu/Eu*, Ce/Ce* and Gd/Gd* in STS of the Mura River reveal that most measurements fall within the "neutral" interval close to the value of 1, while the corresponding values for SPM vary significantly in the upper part, while in the lower part settle around 1. Positive Eu anomaly in SPM was observed in Zone 1. Since there is no recognised significant anthropogenic source of Eu, this anomaly can be attributed to a natural Eu anomaly found in host rocks. This is particularly true for the highly metamorphosed rocks and granitic intrusions of the Tauern window, which underlie the Austroalpine units that are predominantly present in Zone 2. The spatial distribution of Ce and Gd anomalies (Fig. 7) in the main channel and its tributaries shows a negative anomaly in Zone 1. This anomaly shifts to a positive anomaly in the uppermost part of Zone 2, after which the values normalize to a slightly positive anomaly downstream. Further studies are needed to sufficiently explain which natural phenomena is the cause for these observed variations.

The comprehensive assessment of the STS in the main channel and tributaries allows the proposal of geochemical background levels for the Mura River system. However, results from various methods used to determine these geochemical backgrounds vary significantly, as is the case for Hf, Mo, Se, Sn, W, where deviations for a factor up to 5 were observed (Table 2). It has become clear that determining the natural geochemical background, due to potential past and present anthropogenic influences, cannot be accomplished by using a single method, but needs to be assessed for each element separately by carefully observing the spatial distribution of elemental levels in the STS. In the Mura River case, the 75th percentile, or the value of the MD2MAD parameter, has been suggested as the appropriate measure for natural geochemical background in most cases. When the locations with elevated elemental levels corresponded to potential anthropogenic sources, the percentile values from Zone 1 were used to avoid potential anthropogenic influences on the estimation of the natural background. This was the case for Hf, Mo, Sn, W, Zn, Se and Zr. Obtained values have been compared to the established internationally recognised target values (“the New Dutch List “) for the soils or sediment (ESDAT, 2000). Suggested background values in the Mura River case are generally below the target values according to the New Dutch List, except for Ba, Co, Mo and Ni. For those elements, target values according to the New Dutch List are 160, 9, 0.5 and 35 mg/kg, while suggested background values for the Mura River are 461, 18, 0.9 and 57 mg/kg respectively. The reason for this is that the natural background levels in the Alps are higher, which means that some elemental levels in STS from the areas with no detected anthropogenic impacts can exceed target values. Contrary, established natural background levels for Sb, As, Cd and Pb in the Mura River case (1.4, 12, 0.28 and 30 mg/kg respectively) are much lower than established target values according to the New Dutch List (3, 29, 0.8 and 85 mg/kg respectively). This means that even if measured metal levels in sediments, and possibly in soils developed on these sediments, do not exceed target values, it does not necessarily indicate that the area was not subjected to past or present anthropogenic impact.

The latter highlights the importance of detailed geochemical surveying on a larger scale for environmental monitoring and assessing anthropogenic impacts. Moreover, comprehensive geochemical data on sediment composition can be valuable for the future detection of natural anomalies, which may indicate the presence of undiscovered ore bodies. Obtained results also suggest possible normalization values, which can be used in future studies, either dealing with anthropogenic impacts, or defining natural enrichment in prospecting tasks.

The main limitation of this study is the question whether SPM samples are representative, because only limited amount of water was sampled and filtered in the field (i.e. limitations of battery life for pump, water sampling accessible only from riverbank etc.). Water for the collection of SPM was collected in a very short timeframe, which was not uniform through the river course. Particularly in the case that heavy precipitation occurs during the sampling campaign comparison of the results of SPM sampling might not be directly comparable before and after heavy rain event. These changes needs to be studied in more details in the future. Certain uncertainties remain considering whether SPM analysis is accurate, because HF was not used in the digestion procedure and acid-resistant silicate particles might not be digested, although quality control results shows that digestion might be complete. Therefore, the results of SPM could only be regarded as suitable as preliminary information about SPM composition and elemental behaviours in the Mura River. More rigorous sampling, filtration and analytical protocols needs to be applied in the case of SPM. Uncertainties arise in the case of multivariate statistical analysis results, because it was done on relatively low amount of data. The identified geochemical groups of elements can serve only as indicative information. By applying multivariate statistics on larger dataset more certain conclusions could be drawn.

The data obtained within this study improve our understanding of elemental behaviour in the Alpine watercourse, while the upper background and threshold values may present an upgrading for the sustainable management of transboundary rivers and expand the dataset on legacy and new pollutants in European river systems.

## Conclusions

This study delivers a comprehensive geochemical assessment of STS and SPM composition and their spatial patterns in geochemical data, identification of geochemical groups of elements, and determination of geochemical background levels for the STS of the Alpine river Mura (Austria, Slovenia, EU). Limitations of the study are linked to the collection and analysis of SPM, and the results of multivariate methods due to relatively low amount of available data. The main conclusions from the study are:

1. higher elemental variability was detected in SPM, compared to STS, which obviously acts as an “averaging agent”. This indicates that SPM might be better media for detecting spatio-temporal changes and source identification;
2. higher variability in elemental levels was observed in STS and SPM of tributaries, compared to STS and SPM in the main channel, especially in the lower part of the river. This means that STS and SPM of tributaries better reflect detect local sources of elements, while main channel acts as an “averaging agent”;
3. the Mura River stream sediments are depleted of Hf, Tl, Zr, Na and K, and more abundant in Cd, Bi, Se, Sb, As, Zn and P compared to the average composition of upper Earth’s crust. This can be most likely linked to geogenic differentiation of elements in the Alps and the weathering of highly metamorphic rocks and other nappes in the Austroalpine unit;
4. multivariate statistical approaches were effective in determining geochemical associations (4 associations in SPMa and 6 associations in STS), which reflect the lithological characteristics of the catchment area, erosion/depositional patterns, various interactions between sediments and water in the fluvial system, and anthropogenic impacts. However, the number of analysis used in this study are low causing uncertainties in the results;
5. YREE levels are higher in STS closer to the source, while YREE levels in SPM are higher closer to the mouth, probably reflecting higher erosion rates in the upper part, and physicochemical weathering of minerals in the lower part;
6. LREEs are more abundant in STS compared to HREEs, while no such trend was observed for SPM, potentially indicating that HREEs are removed from STS by physiochemical processes in sediment, however the process is still unknown;
7. a distinctive Gd anomaly was detected in SPM in the lower parts of the river, potentially indicating anthropogenic impacts;
8. natural background levels for STS have been suggested, which can be also used as normalization values for similar future studies.

## Supplementary Information

Below is the link to the electronic supplementary material.

Supplementary file1 (DOCX 248 KB)

## Data Availability

All data supporting the findings of this study are available within the paper and its Supplementary Information.
