# Supplementary material for: Geochemical properties of stream sediments and suspended particulate matter and determination of geochemical background values for the Alpine River Mura
Source: Environ Geochem Health. 2026 Jul 18;48(11):470. doi: 10.1007/s10653-026-03331-4 (PMC13380562; doi:10.1007/s10653-026-03331-4)
Supplement: Supplementary file 1 — Supplementary file1 (DOCX 248 KB) [file 10653_2026_3331_MOESM1_ESM.docx]

**Supplementary material**

**Geochemical properties of stream sediments and suspended particulate matter and determination of geochemical background values for the Alpine River Mura**

Barbara Čeplak^1^, Ulrike Moser^2^, Johanna Irrgeher^2^, Martin Šala^3^ and Gorazd Žibret^1*^

^1^Geological Survey of Slovenia, Dimičeva ulica 14, 1000 Ljubljana

^2^Montanuniversität Leoben, Chair of General and Analytical Chemistry, Franz-Josef-Strasse 18, 8700 Leoben

^3^National Institute of Chemistry, Hajdrihova ulica 19, 1000 Ljubljana

*corresponding author: [gorazd.zibret@geo-zs.si](mailto:gorazd.zibret@geo-zs.si)

**Content**

[Table S1: The sampling location (sample ID, name of sampling point, approx.. distance from the source, GPS coordinates for longitude and latitude) 1](#_Toc217370980)

[Table S2: Descriptive statistics of 57 elements in STS samples in main channel and tributaries 3](#_Toc217370981)

[Table S3: Descriptive statistics of 36 elements in main channel, 26 in tributaries within low water regime and 32 elements in main channel of average water regime 5](#_Toc217370982)

[Table S4: Element levels of 57 elements in stream sediments (main channel and tributaries); < 0.063 mm 7](#_Toc217370983)

[Table S5: Element levels of 36(M) and 26(T) elements in SPM samples at low water regime (main channel and tributaries) 13](#_Toc217370984)

[Table S6: Element levels of 32 elements in SPM samples at average water regime (main channel) 17](#_Toc217370985)

Table S1: The sampling location (sample ID, name of sampling point, approx. distance from the source, GPS coordinates for longitude and latitude)

| sample ID | zone | name of sampling point | approx. distance from the source [km] | longitude | latitude |
| --- | --- | --- | --- | --- | --- |
| M-00 | 1 | Muhr/Bergle Reservoir/dam | 7 | 13.423323 | 47.122291 |
| M-01 | 1 | Schmalzgraben | 10 | 13.457940 | 47.112030 |
| M-02 | 1 | Schellgaden | 21 | 13.579110 | 47.079710 |
| M-03 | 1 | St. Michael im Lungau | 34 | 13.700800 | 47.089600 |
| M-04 | 1 | Tamsweg | 45 | 13.813495 | 47.119415 |
| M-05 | 1 | Madling | 51 | 13.832976 | 47.075481 |
| M-06 | 1 | Predlitz/Turrach | 58 | 13.907716 | 47.071182 |
| M-07 | 1 | Stadl an der Mur | 67 | 14.008477 | 47.099444 |
| M-08 | 1 | St. Lorenzen | 75 | 14.096291 | 47.103126 |
| M-09 | 1 | St. Egidi | 86 | 14.211268 | 47.111110 |
| M-10 | 1 | Niederwölz | 97 | 14.322238 | 47.138350 |
| M-11 | 2 | St. Georgen. Judenburg | 114 | 14.464954 | 47.211525 |
| M-12 | 2 | St. Peter. Judenburg | 129 | 14.607872 | 47.183191 |
| M-13 | 2 | Zeltweg | 149 | 14.771388 | 47.184373 |
| M-14 | 2 | St. Lorenzen b. Knittelfeld | 166 | 14.894840 | 47.253570 |
| M-15 | 2 | St. Michael in der Obersteiermark | 183 | 15.011246 | 47.333086 |
| M-16 | 2 | Leoben | 197 | 15.089030 | 47.383690 |
| M-17 | 2 | Niklasdorf | 205 | 15.148989 | 47.399506 |
| M-18 | 2 | Unteraich/Oberaich | 215 | 15.250160 | 47.404170 |
| M-19 | 2 | Bruck an der Mur | 218 | 15.284226 | 47.409584 |
| M-20 | 2 | Mixnitz/Pernegg | 232 | 15.368470 | 47.319770 |
| M-21 | 2 | Frohnleiten | 243 | 15.322302 | 47.266943 |
| M-22 | 2 | Peggau- Deutschfeistritz | 257 | 15.320353 | 47.174693 |
| M-23 | 2 | Gratkorn | 267 | 15.374300 | 47.118560 |
| M-24 | 2 | Graz/Augarten | 275 | 15.434961 | 47.064127 |
| M-25 | 2 | Graz Dörfla/Gössendorf | 281 | 15.461854 | 47.011094 |
| M-26 | 3 | Mellach | 298 | 15.518940 | 46.885970 |
| M-27 | 3 | Bachsdorf | 302 | 15.540623 | 46.853110 |
| M-28 | 3 | Retznei/Ehrenhausen | 313 | 15.572262 | 46.765379 |
| M-29 | 3 | Spielfeld | 324 | 15.649501 | 46.706286 |
| M-30 | 3 | Sladki vrh/Paloma | 332 | 15.751770 | 46.697580 |
| M-31 | 3 | Cmurek | 336 | 15.801360 | 46.714870 |
| M-32 | 3 | Apače | 346 | 15.919980 | 46.705200 |
| M-33 | 3 | Mele | 355 | 16.012040 | 46.663660 |
| M-34 | 3 | Hrastje/Mota | 364 | 16.092700 | 46.615750 |
| M-35 | 3 | Veržej | 371 | 16.168150 | 46.593010 |
| M-36 | 3 | Brod/Krapje | 378 | 16.240366 | 46.550869 |
| M-37 | 3 | Gibina | 385 | 16.317309 | 46.526380 |
| M-38 | 3 | Petišovci/Mursko Središće | 396 | 16.444069 | 46.515891 |
| M-39 | 3 | Pince | 402 | 16.497814 | 46.498309 |
| M-40 | 3 | Dekanovec | 414 | 16.585118 | 46.472390 |
| T-01 | ns | Rothglüdenbach | 9 | 13.436651 | 47.113562 |
| T-02 | ns | Muhr | 22 | 13.583132 | 47.079980 |
| T-03 | ns | Zederhausbach | 26 | 13.602011 | 47.097361 |
| T-04 | ns | Taurach | 43 | 13.805657 | 47.141452 |
| T-05 | ns | Rantenbach | 82 | 14.171514 | 47.116360 |
| T-06 | ns | Wölzer Bach | 103 | 14.373674 | 47.150797 |
| T-07 | ns | Pölsbach | 146 | 14.736360 | 47.186827 |
| T-08 | ns | Granitzenbach | 148 | 14.763009 | 47.181206 |
| T-09 | ns | Sonngrabenbach | 175 | 14.947759 | 47.297808 |
| T-10 | ns | Lobmingbach | 180 | 14.988006 | 47.317105 |
| T-11 | ns | Liesing | 184 | 15.013788 | 47.344197 |
| T-12 | ns | Gößbach | 195 | 15.107923 | 47.362150 |
| T-13 | ns | Vordernberger Bach | 197 | 15.089013 | 47.376046 |
| T-14 | ns | Mürz | 217 | 15.285377 | 47.438565 |
| T-15 | ns | Breitenauerbach | 229 | 15.361088 | 47.347025 |
| T-16 | ns | Gamsgraben | 240 | 15.271343 | 47.284347 |
| T-17 | ns | Rötschbach | 260 | 15.334312 | 47.165453 |
| T-18 | ns | Grazbach | 275 | 15.434961 | 47.064127 |
| T-19 | ns | Raababach | 284 | 15.476591 | 46.997807 |
| T-20 | ns | Sulm | 315 | 15.544173 | 46.757859 |
| T-21 | ns | Drauchenbach | 355 | 15.989354 | 46.692367 |
| T-22 | ns | Boračevski potok | 359 | 16.046938 | 46.641362 |
| T-23 | ns | Razbremenilni kanal Ledav - Mura | 370 | 16.163822 | 46.609481 |
| T-24 | ns | Ščavnica | 384 | 16.287192 | 46.522351 |
| T-25 | ns | Ledava | 416 | 16.503905 | 46.513704 |

ns – not specific, M – main channel, T – tributary

Table S2: Descriptive statistics of 57 elements in STS samples in main channel and tributaries

| El. | unit | LOD | ARPD (%) | R (%) | main channel | | | | | tributaries | | | | |
| --- | --- | --- | --- | --- | --- | --- | --- | --- | --- | --- | --- | --- | --- | --- |
|  |  |  |  |  | min | Q1 | me | Q3 | max | min | Q1 | me | Q3 | max |
|  |  |  |  |  | <0.063 mm | | | | | <0.063 mm | | | | |
| Ag | mg/kg | 0.02 | 16 | 90 | 0.05 | 0.09 | 0.09 | 0.12 | 0.50 | 0.03 | 0.04 | 0.08 | 0.13 | 0.46 |
| Al | % | 0.01 | 3 | 98 | 6.5 | 6.9 | 7.2 | 8.0 | 9.5 | 3.8 | 6.05 | 7.1 | 7.7 | 8.1 |
| As | mg/kg | 0.2 | 7 | 95 | 6.9 | 8.7 | 11 | 12 | 37 | 2.8 | 8.05 | 12 | 14 | 220 |
| Ba | mg/kg | 1 | 4 | 101 | 205 | 380 | 420 | 470 | 675 | 200 | 310 | 380 | 510 | 720 |
| Be | mg/kg | 1 | 5 | 94 | 1.0 | 2.0 | 2.0 | 2.0 | 3.0 | 0.50 | 1.0 | 2.0 | 2.0 | 3.0 |
| Bi | mg/kg | 0.04 | 11 | 102 | 0.23 | 0.28 | 0.32 | 0.37 | 1.2 | 0.12 | 0.20 | 0.30 | 0.38 | 1.2 |
| Ca | % | 0.01 | 3 | 98 | 2.0 | 3.1 | 3.0 | 4.2 | 9.0 | 0.91 | 1.7 | 3.5 | 4.9 | 12 |
| Cd | mg/kg | 0.02 | 8 | 94 | 0.01 | 0.21 | 0.23 | 0.28 | 0.39 | 0.13 | 0.19 | 0.22 | 0.27 | 0.50 |
| Ce | mg/kg | 0.02 | 5 | 99 | 79 | 90 | 99 | 120 | 250 | 44 | 71 | 80 | 125 | 320 |
| Co | mg/kg | 0.2 | 3 | 105 | 8.8 | 14 | 16 | 18 | 27 | 8.9 | 15 | 17 | 20 | 57 |
| Cr | mg/kg | 1 | 4 | 96 | 43 | 80 | 85 | 100 | 140 | 40 | 80 | 93 | 140 | 1900 |
| Cs | mg/kg | 0.1 | 5 | 99 | 1.0 | 4.1 | 4.6 | 5.0 | 7.7 | 1.8 | 3.5 | 4.7 | 5.4 | 7.9 |
| Cu | mg/kg | 0.1 | 5 | 100 | 11 | 24 | 29 | 33 | 58 | 17 | 26 | 31 | 40 | 76 |
| Dy | mg/kg | 0.1 | 5 | 94 | 4.4 | 5.0 | 5.9 | 6.9 | 11 | 2.8 | 4.0 | 5.1 | 7.4 | 14 |
| Er | mg/kg | 0.1 | 4 | 90 | 2.2 | 2.5 | 3.0 | 3.5 | 6.2 | 1.4 | 2.0 | 2.3 | 4.2 | 6.5 |
| Eu | mg/kg | 0.1 | 4 | 94 | 1.5 | 1.6 | 1.9 | 2.2 | 3.9 | 0.90 | 1.3 | 1.6 | 2.4 | 4.2 |
| Fe | % | 0.01 | 2 | 100 | 3.5 | 3.9 | 4.4 | 5.0 | 6.9 | 2.3 | 4.0 | 4.5 | 5.2 | 8.0 |
| Ga | mg/kg | 0.02 | 3 | 97 | 12 | 15 | 17 | 18 | 23 | 8.5 | 14 | 17 | 18 | 23 |
| Gd | mg/kg | 0.1 | 5 | 95 | 5.8 | 6.5 | 7.2 | 8.8 | 15 | 3.9 | 5.3 | 6.5 | 9.3 | 20 |
| Hf | mg/kg | 0.02 | 7 | 87 | 0.08 | 0.27 | 0.49 | 0.60 | 0.74 | 0.06 | 0.31 | 0.58 | 0.78 | 1.3 |
| Ho | mg/kg | 0.1 | 5 | 86 | 0.75 | 0.90 | 1.1 | 1.2 | 2.1 | 0.50 | 0.70 | 0.85 | 1.4 | 2.3 |
| In | mg/kg | 0.01 | 7 | 100 | 0.06 | 0.07 | 0.07 | 0.08 | 0.11 | 0.04 | 0.06 | 0.07 | 0.08 | 0.12 |
| K | % | 0.01 | 4 | 101 | 0.70 | 1.5 | 1.6 | 1.8 | 2.8 | 0.69 | 1.1 | 1.5 | 1.8 | 2.9 |
| La | mg/kg | 0.1 | 6 | 98 | 39 | 44 | 49 | 58 | 120 | 20 | 35 | 39 | 62 | 160 |
| Li | mg/kg | 0.1 | 5 | 99 | 8.9 | 36 | 40 | 45 | 53 | 16 | 23 | 37 | 49 | 55 |
| Lu | mg/kg | 0.1 | 6 | 93 | 0.30 | 0.30 | 0.30 | 0.40 | 0.8 | 0.20 | 0.20 | 0.30 | 0.50 | 0.80 |
| Mg | % | 0.01 | 2 | 100 | 0.92 | 1.7 | 2.0 | 2.2 | 3.6 | 0.93 | 1.2 | 1.7 | 2.8 | 7.4 |
| Mn | mg/kg | 1 | 3 | 102 | 830 | 940 | 1100 | 1200 | 1900 | 580 | 880 | 1150 | 1500 | 2400 |
| Mo | mg/kg | 0.05 | 18 | 94 | 0.36 | 0.84 | 1.4 | 1.7 | 3.5 | 0.44 | 0.61 | 0.91 | 1.2 | 25 |
| Na | % | 0.001 | 2 | 102 | 1.3 | 1.4 | 1.5 | 1.6 | 3.6 | 0.65 | 0.87 | 1.3 | 1.5 | 2.1 |
| Nb | mg/kg | 0.04 | 5 | 91 | 4.4 | 10 | 12 | 14 | 23 | 3.6 | 8.5 | 9.6 | 15 | 24 |
| Nd | mg/kg | 0.1 | 5 | 99 | 36 | 41 | 44 | 54 | 110 | 23 | 33 | 36 | 58 | 140 |
| Ni | mg/kg | 0.1 | 4 | 102 | 15 | 41 | 49 | 57 | 81 | 22 | 39 | 47 | 74 | 710 |
| P | % | 0.001 | 4 | 95 | 0.10 | 0.11 | 0.14 | 0.16 | 0.24 | 0.06 | 0.10 | 0.13 | 0.19 | 0.24 |
| Pb | mg/kg | 0.02 | 9 | 99 | 16 | 25 | 28 | 30 | 46 | 12 | 17 | 27 | 35 | 44 |
| Pr | mg/kg | 0.1 | 5 | 97 | 10 | 11 | 12 | 14 | 29 | 5.4 | 8.1 | 9.55 | 15 | 37 |
| Rb | mg/kg | 0.1 | 6 | 100 | 24 | 75 | 81 | 85 | 120 | 35 | 51 | 67 | 91 | 115 |
| Sb | mg/kg | 0.02 | 14 | 92 | 0.34 | 1.1 | 1.2 | 1.4 | 2.2 | 0.28 | 0.75 | 1.1 | 1.5 | 8.0 |
| Sc | mg/kg | 0.1 | 2 | 99 | 13 | 15 | 18 | 20 | 33 | 8.8 | 13 | 15 | 22 | 29 |
| Se | mg/kg | 0.002 | 29 | 111 | 0.15 | 0.30 | 0.45 | 0.60 | 1.1 | 0.50 | 0.63 | 0.80 | 1.0 | 1.3 |
| Sm | mg/kg | 0.1 | 6 | 97 | 7.1 | 7.9 | 8.6 | 10 | 20 | 4.5 | 6.4 | 7.4 | 11 | 25 |
| Sn | mg/kg | 0.1 | 11 | 92 | 1.7 | 2.5 | 3.2 | 3.7 | 7.3 | 1.4 | 2.2 | 3.1 | 4.3 | 7.0 |
| Sr | mg/kg | 1 | 3 | 100 | 160 | 180 | 210 | 233 | 900 | 92 | 135 | 160 | 240 | 720 |
| Ta | mg/kg | 0.1 | 7 | 85 | 0.30 | 0.70 | 0.80 | 1.0 | 1.8 | 0.30 | 0.65 | 0.75 | 1.0 | 2.0 |
| Tb | mg/kg | 0.1 | 5 | 87 | 0.75 | 0.90 | 1.0 | 1.2 | 2.0 | 0.50 | 0.70 | 0.90 | 1.3 | 2.5 |
| Te | mg/kg | 0.05 | 26 | 107 | 0.03 | 0.05 | 0.06 | 0.07 | 0.16 | 0.03 | 0.05 | 0.07 | 0.09 | 0.52 |
| Th | mg/kg | 0.1 | 6 | 93 | 9.5 | 12 | 13 | 15 | 32 | 4.1 | 8.2 | 9.7 | 17 | 48 |
| Ti | % | 0.001 | 3 | 100 | 0.44 | 0.50 | 0.53 | 0.59 | 1.1 | 0.25 | 0.37 | 0.46 | 0.68 | 1.1 |
| Tl | mg/kg | 0.05 | 5 | 102 | 0.03 | 0.39 | 0.43 | 0.48 | 0.69 | 0.23 | 0.33 | 0.42 | 0.60 | 0.83 |
| Tm | mg/kg | 0.1 | 2 | 90 | 0.30 | 0.30 | 0.40 | 0.50 | 0.90 | 0.20 | 0.30 | 0.30 | 0.60 | 0.90 |
| U | mg/kg | 0.1 | 8 | 91 | 2.1 | 2.4 | 2.6 | 3.0 | 4.8 | 1.4 | 2.0 | 2.3 | 3.3 | 8.5 |
| V | mg/kg | 2 | 3 | 99 | 92 | 100 | 110 | 125 | 170 | 65 | 100 | 110 | 130 | 160 |
| W | mg/kg | 0.1 | 22 | 93 | 0.50 | 1.3 | 2.4 | 3.0 | 4.4 | 0.50 | 0.90 | 1.4 | 2.1 | 23 |
| Y | mg/kg | 0.1 | 3 | 92 | 22 | 25 | 28 | 33 | 61 | 14 | 19 | 22 | 40 | 62 |
| Yb | mg/kg | 0.1 | 4 | 92 | 2.0 | 2.2 | 2.6 | 2.9 | 5.6 | 1.3 | 1.8 | 1.9 | 3.8 | 5.7 |
| Zn | mg/kg | 0.2 | 3 | 99 | 32 | 93 | 100 | 110 | 180 | 63 | 85 | 98 | 120 | 200 |
| Zr | mg/kg | 0.2 | 5 | 86 | 2.0 | 10 | 19 | 21 | 24 | 1.3 | 9.3 | 17 | 28 | 46 |

Abbreviations: El – element; LOD – limit of detection; ARPD (%) – average relative percent difference; R (%) – recovery rate; min – minimum; Q1 – 25% quartile; Md – median; Q3 – 75% quartile; max – maximum

Table S3: Descriptive statistics of 36 elements in main channel, 26 in tributaries within low water regime and 32 elements in main channel of average water regime

| El | unit | LOD | R (%) | blank | LOW | | | | | | | | | | AVERAGE | | | | |
| --- | --- | --- | --- | --- | --- | --- | --- | --- | --- | --- | --- | --- | --- | --- | --- | --- | --- | --- | --- |
|  |  |  |  |  | MAIN CHANNEL | | | | | TRIBUTARIES | | | | | MAIN CHANNEL | | | | |
|  |  |  |  |  | Min | Q1 | Md | Q3 | Max | Min | Q1 | Md | Q3 | Max | Min | Q1 | Md | Q3 | Max |
| Ag | μg/L | 1 | 83 | <LOD | <LOD | 2.8 | 3.3 | 5.8 | 22 | <LOD | <LOD | <LOD | <LOD | <LOD | <LOD | <LOD | <LOD | <LOD | <LOD |
| Al | mg/L | 1 | 21 | <LOD | <LOD | 18 | 31 | 110 | 260 | 11 | 25 | 52 | 73 | 390 | 17 | 150 | 280 | 410 | 1500 |
| As | μg/L | 1 | 94 | <LOD | 16 | 54 | 83 | 110 | 340 | 1.0 | 17 | 39 | 63 | 410 | 3.2 | 140 | 220 | 330 | 960 |
| Ba | μg/L | 10 | 18 | <LOD | <LOD | 180 | 400 | 860 | 2400 | 10 | 165 | 300 | 640 | 3100 | 94 | 1000 | 1800 | 2600 | 9000 |
| Bi | μg/L | 1 | 94 | <LOD | <LOD | 1.2 | 6.4 | 8.3 | 15 | <LOD | <LOD | <LOD | <LOD | <LOD | <LOD | <LOD | <LOD | <LOD | <LOD |
| Ca | mg/L | 5 | 7 | <LOD | <LOD | 7.4 | 12 | 16 | 75 | <LOD | <LOD | <LOD | <LOD | <LOD | 100 | 280 | 330 | 370 | 1300 |
| Cd | μg/L | 1 | 98 | <LOD | <LOD | 0.94 | 5.6 | 7.0 | 23 | <LOD | <LOD | <LOD | <LOD | <LOD | <LOD | <LOD | <LOD | <LOD | <LOD |
| Ce | μg/L | 1 | 97 | <LOD | 3.4 | 28 | 54 | 160 | 540 | 6.1 | 22 | 63 | 100 | 500 | 8.7 | 190 | 400 | 570 | 2000 |
| Co | μg/L | 1 | 95 | <LOD | 2.0 | 11 | 34 | 65 | 200 | 2.7 | 9.3 | 22 | 33 | 140 | 2.0 | 62 | 140 | 220 | 620 |
| Cr | μg/L | 10 | 37 | <LOD | 290 | 340 | 470 | 550 | 880 | 140 | 310 | 355 | 450 | 880 | 480 | 810 | 1100 | 1500 | 2700 |
| Cu | μg/L | 5 | 105 | 220 | 64 | 175 | 270 | 350 | 1300 | 48 | 96 | 140 | 235 | 750 | <LOD | <LOD | <LOD | <LOD | <LOD |
| Dy | μg/L | 1 | 74 | <LOD | <LOD | 2.5 | 4.1 | 12 | 39 | <LOD | 1.5 | 4.4 | 7.8 | 37 | 2.0 | 12 | 24 | 32 | 96 |
| Er | μg/L | 1 | 63 | <LOD | <LOD | 1.1 | 2.0 | 6.1 | 21 | <LOD | 1.1 | 2.0 | 3.9 | 21 | 2.0 | 4.0 | 12 | 16 | 44 |
| Eu | μg/L | 1 | 65 | <LOD | <LOD | <LOD | 1.1 | 3.7 | 12 | <LOD | <LOD | <LOD | <LOD | <LOD | 2.0 | 4.0 | 8.0 | 8.0 | 32 |
| Fe | mg/L | 0.05 | 69 | <LOD | 4.9 | 38 | 65 | 180 | 390 | 5.0 | 29 | 69 | 140 | 920 | 14 | 140 | 290 | 420 | 1200 |
| Ga | μg/L | 5 | 163 | <LOD | <LOD | 36 | 75 | 180 | 490 | 5.0 | 36 | 62 | 110 | 630 | 10 | 57 | 100 | 160 | 580 |
| Gd | μg/L | 1 | 111 | <LOD | <LOD | 3.1 | 5.4 | 18 | 57 | <LOD | 2.0 | 6.9 | 11 | 53 | 2.0 | 12 | 32 | 40 | 140 |
| La | μg/L | 1 | 95 | <LOD | 1.5 | 16 | 29 | 83 | 260 | 3.6 | 9.4 | 31 | 51 | 260 | 4.1 | 83 | 180 | 255 | 855 |
| Li | μg/L | 5 | 73 | 67 | <LOD | 3.6 | 37 | 79 | 295 | <LOD | <LOD | <LOD | <LOD | <LOD | 45 | 160 | 320 | 440 | 1200 |
| Mg | mg/L | 1 | 82 | 2.8 | 8.5 | 14 | 25 | 42 | 135 | 4.0 | 15 | 21 | 31 | 73 | 8.7 | 62 | 110 | 150 | 380 |
| Mn | μg/L | 5 | 86 | <LOD | 290 | 1100 | 5500 | 6800 | 28000 | 220 | 650 | 1500 | 2300 | 20000 | 690 | 4800 | 15000 | 19000 | 34000 |
| Na | mg/L | 0.05 | 4 | <LOD | 10 | 12 | 14 | 16 | 36 | <LOD | <LOD | <LOD | <LOD | <LOD | 21 | 33 | 37 | 41 | 65 |
| Nd | μg/L | 1 | 95 | <LOD | <LOD | 17 | 29 | 75 | 245 | 3.6 | 8.9 | 28 | 48 | 240 | 5.3 | 84 | 180 | 260 | 560 |
| Ni | μg/L | 50 | 94 | <LOD | <LOD | 67 | 170 | 250 | 1500 | <LOD | <LOD | <LOD | <LOD | <LOD | <LOD | 210 | 550 | 790 | 1900 |
| Pb | μg/L | 1 | 78 | <LOD | 22 | 44 | 180 | 240 | 1400 | 7.0 | 53 | 93 | 150 | 1000 | 22 | 130 | 350 | 450 | 1400 |
| Pr | μg/L | 1 | 94 | <LOD | <LOD | 3.6 | 6.9 | 19 | 63 | <LOD | 2.6 | 7.5 | 12 | 60 | 2.0 | 20 | 44 | 64 | 220 |
| Rb | μg/L | 1 | 21 | <LOD | 6.3 | 29 | 56 | 170 | 430 | 1.8 | 18 | 76 | 120 | 590 | 26 | 180 | 360 | 500 | 1800 |
| Sc | μg/L | 5 | 59 | <LOD | <LOD | <LOD | <LOD | <LOD | <LOD | <LOD | <LOD | <LOD | <LOD | <LOD | <LOD | 20 | 44 | 60 | 240 |
| Sm | μg/L | 1 | 91 | <LOD | <LOD | 3.1 | 5.2 | 14 | 50 | <LOD | 2.2 | 6.9 | 10 | 46 | 2.0 | 20 | 36 | 48 | 170 |
| Sr | μg/L | 5 | 22 | <LOD | 170 | 240 | 290 | 370 | 1100 | 21 | 100 | 190 | 420 | 950 | 200 | 590 | 690 | 850 | 2700 |
| Th | μg/L | 1 | 86 | <LOD | <LOD | <LOD | <LOD | <LOD | <LOD | <LOD | <LOD | <LOD | <LOD | <LOD | 2.8 | 27 | 75 | 91 | 360 |
| Ti | mg/L | 0.01 | 19 | <LOD | 0.08 | 0.62 | 1.1 | 3.1 | 8.5 | 0.11 | 0.45 | 1.3 | 1.8 | 11 | 0.42 | 3.5 | 7.1 | 11 | 34 |
| U | μg/L | 0.5 | 60 | <LOD | <LOD | 1.9 | 2.9 | 5.3 | 18 | <LOD | 1.5 | 2.6 | 4.2 | 12 | 4.0 | 8.0 | 12 | 20 | 76 |
| V | μg/L | 5 | 57 | <LOD | <LOD | 37 | 78 | 220 | 530 | <LOD | 31 | 99 | 130 | 680 | 10 | 210 | 430 | 610 | 2200 |
| Y | μg/L | 1 | 65 | <LOD | <LOD | 7.0 | 20 | 55 | 210 | <LOD | 3.5 | 21 | 41 | 190 | 8.0 | 60 | 130 | 170 | 490 |
| Yb | μg/L | 1 | 52 | <LOD | <LOD | <LOD | 1.8 | 5.5 | 18 | <LOD | 1.0 | 1.9 | 3.9 | 17 | 2.0 | 4.0 | 12 | 16 | 44 |
| Zn | μg/L | 10 | 97 | <LOD | 29 | 770 | 1100 | 1900 | 3600 | <LOD | <LOD | <LOD | <LOD | <LOD | <LOD | <LOD | <LOD | <LOD | <LOD |
| Zr | μg/L | 1 | 7 | <LOD | <LOD | <LOD | 10 | 22 | 1900 | <LOD | <LOD | <LOD | <LOD | <LOD | <LOD | <LOD | <LOD | <LOD | <LOD |

Abbreviations: El – element; LOD – limit of detection; blank – elemental levels in blank filters; R (%) – recovery rate; Min – minimum; Q1 – 25%

quartile; Md – median; Q3 – 75% quartile; Max – maximum

**Table S4:** Element levels of 57 elements in stream sediments (main channel and tributaries); < 0.063 mm

|  | Ag | Al | As | Ba | Be | Bi | Ca | Cd | Ce | Co | Cr | Cs | Cu | Dy | Er | Eu | Fe | Ga | Gd | Hf |
| --- | --- | --- | --- | --- | --- | --- | --- | --- | --- | --- | --- | --- | --- | --- | --- | --- | --- | --- | --- | --- |
| unit | mg/kg | % | mg/kg | mg/kg | mg/kg | mg/kg | % | mg/kg | mg/kg | mg/kg | mg/kg | mg/kg | mg/kg | mg/kg | mg/kg | mg/kg | % | mg/kg | mg/kg | mg/kg |
| LOD | 0.02 | 0.01 | 0.2 | 1 | 1 | 0.04 | 0.01 | 0.02 | 0.02 | 0.2 | 1 | 0.1 | 0.1 | 0.1 | 0.1 | 0.1 | 0.01 | 0.02 | 0.1 | 0.02 |
| M-00 | 0.11 | 9.5 | 26 | 510 | 2.0 | 0.43 | 2.9 | 0.25 | 130 | 25 | 130 | 7.7 | 58 | 7.1 | 3.2 | 2.5 | 5.1 | 21 | 9.8 | 0.09 |
| M-01 | 0.25 | 9.1 | 37 | 675 | 3.0 | 0.45 | 2.7 | 0.39 | 90 | 27 | 140 | 7.3 | 58 | 5.9 | 3.0 | 1.9 | 5.3 | 22 | 7.2 | 0.08 |
| M-02 | 0.05 | 9.1 | 12 | 260 | 2.0 | 0.41 | 5.5 | 0.13 | 110 | 8.8 | 43 | 1.0 | 11 | 11 | 6.2 | 3.2 | 3.6 | 23 | 12 | 0.14 |
| M-03 | 0.09 | 6.9 | 12 | 205 | 1.0 | 0.27 | 8.5 | 0.01 | 180 | 19 | 99 | 3.2 | 53 | 8.6 | 3.7 | 3.0 | 4.3 | 15 | 12 | 0.10 |
| M-04 | 0.08 | 6.9 | 15 | 320 | 1.0 | 0.25 | 6.8 | 0.21 | 125 | 18 | 87 | 4.3 | 39 | 7.1 | 3.4 | 2.2 | 4.5 | 16 | 9.1 | 0.15 |
| M-05 | 0.09 | 8.0 | 11 | 420 | 2.0 | 0.29 | 5.2 | 0.18 | 130 | 17 | 85 | 3.8 | 39 | 6.7 | 3.2 | 2.2 | 4.2 | 17 | 8.8 | 0.17 |
| M-06 | 0.10 | 6.7 | 12 | 260 | 2.0 | 0.24 | 5.5 | 0.24 | 180 | 16 | 84 | 2.5 | 33 | 9.2 | 4.4 | 3.0 | 5.2 | 14 | 12 | 0.24 |
| M-07 | 0.11 | 6.5 | 12 | 230 | 1.0 | 0.37 | 6.1 | 0.25 | 250 | 16 | 86 | 2.1 | 42 | 11 | 5.5 | 3.9 | 6.1 | 12 | 15 | 0.33 |
| M-08 | 0.09 | 7.4 | 11 | 370 | 2.0 | 0.26 | 4.4 | 0.18 | 105 | 16 | 78 | 3.6 | 33 | 5.7 | 2.7 | 1.9 | 4.2 | 16 | 7.6 | 0.23 |
| M-09 | 0.07 | 7.0 | 11 | 350 | 2.0 | 0.23 | 3.9 | 0.19 | 99 | 13 | 61 | 2.9 | 25 | 6.0 | 3.1 | 1.9 | 4.2 | 15 | 7.3 | 0.25 |
| M-10 | 0.12 | 7.2 | 14 | 370 | 2.0 | 0.30 | 4.1 | 0.26 | 160 | 16 | 74 | 3.6 | 29 | 8.5 | 4.4 | 2.8 | 5.7 | 15 | 11 | 0.43 |
| M-11 | 0.09 | 8.0 | 13 | 490 | 3.0 | 0.26 | 3.4 | 0.19 | 110 | 18 | 90 | 4.8 | 31 | 5.9 | 2.9 | 2.0 | 4.6 | 18 | 7.6 | 0.27 |
| M-12 | 0.09 | 7.8 | 10 | 440 | 2.0 | 0.28 | 3.2 | 0.19 | 120 | 16 | 79 | 4.4 | 27 | 6.4 | 3.2 | 2.1 | 4.8 | 17 | 8.3 | 0.31 |
| M-13 | 0.08 | 8.2 | 10 | 470 | 3.0 | 0.29 | 3.1 | 0.23 | 120 | 18 | 94 | 5.1 | 30 | 6.6 | 3.2 | 2.2 | 5.2 | 19 | 8.5 | 0.33 |
| M-14 | 0.07 | 7.8 | 11 | 430 | 3.0 | 0.26 | 3.2 | 0.21 | 110 | 17 | 83 | 4.3 | 26 | 6.3 | 3.2 | 2.1 | 5.0 | 18 | 8.1 | 0.36 |
| M-15 | 0.06 | 7.6 | 12 | 340 | 2.0 | 0.32 | 3.8 | 0.27 | 200 | 19 | 140 | 3.2 | 23 | 11 | 5.5 | 3.3 | 6.9 | 16 | 14 | 0.67 |
| M-16 | 0.12 | 8.7 | 12 | 560 | 3.0 | 0.35 | 2.4 | 0.30 | 87 | 21 | 110 | 4.8 | 41 | 6.9 | 3.5 | 1.6 | 4.9 | 18 | 6.5 | 0.43 |
| M-17 | 0.09 | 8.0 | 11 | 440 | 2.0 | 0.32 | 3.7 | 0.26 | 120 | 19 | 110 | 4.9 | 32 | 6.9 | 3.5 | 2.2 | 5.3 | 18 | 8.8 | 0.45 |
| M-18 | 0.09 | 8.0 | 13 | 480 | 3.0 | 0.31 | 2.9 | 0.21 | 99 | 18 | 99 | 5.1 | 29 | 5.7 | 2.8 | 1.9 | 4.7 | 19 | 7.3 | 0.42 |
| M-19 | 0.11 | 7.6 | 9.2 | 415 | 2.0 | 0.28 | 3.3 | 0.24 | 130 | 18 | 120 | 4.4 | 27 | 7.4 | 3.8 | 2.4 | 5.6 | 17 | 9.4 | 0.50 |
| M-20 | 0.16 | 8.5 | 17 | 600 | 3.0 | 0.36 | 3.4 | 0.28 | 89 | 22 | 99 | 7.0 | 39 | 4.8 | 2.3 | 1.6 | 4.6 | 22 | 6.5 | 0.59 |
| M-21 | 0.07 | 8.1 | 11 | 470 | 2.0 | 0.28 | 3.9 | 0.28 | 100 | 21 | 120 | 5.5 | 31 | 7.3 | 3.9 | 2.2 | 5.4 | 19 | 8.8 | 0.74 |
| M-22 | 0.09 | 8.0 | 10 | 545 | 2.0 | 0.29 | 4.0 | 0.30 | 100 | 20 | 110 | 5.4 | 28 | 6.2 | 3.1 | 1.9 | 4.7 | 18 | 7.6 | 0.62 |
| M-23 | 0.10 | 7.2 | 14 | 490 | 2.0 | 0.37 | 4.8 | 0.30 | 92 | 18 | 110 | 5.0 | 32 | 5.4 | 2.8 | 1.7 | 4.5 | 17 | 7.0 | 0.66 |
| M-24 | 0.14 | 7.0 | 10 | 450 | 2.0 | 0.36 | 5.9 | 0.33 | 89 | 17 | 100 | 4.8 | 34 | 5.4 | 2.8 | 1.7 | 4.3 | 16 | 6.6 | 0.62 |
| M-25 | 0.12 | 7.4 | 9.1 | 440 | 2.0 | 0.35 | 4.3 | 0.31 | 91 | 16 | 110 | 5.4 | 32 | 5.9 | 3.0 | 1.8 | 4.5 | 17 | 7.1 | 0.56 |
| M-26 | 0.09 | 6.9 | 8.4 | 420 | 2.0 | 0.28 | 3.3 | 0.23 | 79 | 13 | 76 | 4.5 | 22 | 4.4 | 2.2 | 1.5 | 3.5 | 16 | 5.9 | 0.60 |
| M-27 | 0.10 | 7.6 | 9.0 | 420 | 2.0 | 0.31 | 4.2 | 0.25 | 92 | 16 | 100 | 4.8 | 27 | 5.7 | 2.9 | 1.8 | 4.4 | 17 | 7.0 | 0.71 |
| M-28 | 0.18 | 7.2 | 10 | 435 | 2.0 | 0.44 | 3.0 | 0.29 | 83 | 15 | 77 | 2.9 | 32 | 6.0 | 3.1 | 1.6 | 3.9 | 15 | 6.1 | 0.25 |
| M-29 | 0.08 | 7.0 | 7.2 | 390 | 2.0 | 0.26 | 2.9 | 0.20 | 89 | 14 | 80 | 4.3 | 21 | 4.9 | 2.5 | 1.6 | 3.8 | 16 | 6.4 | 0.49 |
| M-30 | 0.50 | 6.6 | 7.1 | 400 | 2.0 | 0.26 | 3.2 | 0.21 | 100 | 12 | 83 | 4.5 | 22 | 5.2 | 2.5 | 1.7 | 3.8 | 15 | 6.9 | 0.62 |
| M-31 | 0.10 | 6.9 | 6.9 | 400 | 2.0 | 0.31 | 3.2 | 0.22 | 90 | 14 | 79 | 4.9 | 24 | 4.6 | 2.3 | 1.5 | 3.8 | 16 | 6.2 | 0.54 |
| M-32 | 0.46 | 7.0 | 8.3 | 410 | 2.0 | 0.32 | 3.0 | 0.23 | 90 | 14 | 82 | 4.9 | 25 | 4.8 | 2.4 | 1.6 | 3.9 | 16 | 6.5 | 0.60 |
| M-33 | 0.10 | 6.9 | 8.4 | 410 | 2.0 | 1.2 | 3.3 | 0.21 | 86 | 14 | 81 | 4.5 | 22 | 4.7 | 2.3 | 1.5 | 3.8 | 15 | 6.2 | 0.61 |
| M-34 | 0.09 | 6.8 | 8.6 | 390 | 2.0 | 0.43 | 3.2 | 0.22 | 100 | 14 | 85 | 4.8 | 24 | 6.9 | 3.5 | 1.7 | 4.0 | 18 | 7.0 | 0.43 |
| M-35 | 0.09 | 6.9 | 8.5 | 400 | 2.0 | 0.38 | 3.2 | 0.21 | 91 | 14 | 80 | 4.6 | 23 | 4.9 | 2.4 | 1.6 | 3.9 | 16 | 6.5 | 0.61 |
| M-36 | 0.09 | 6.8 | 8.4 | 400 | 2.0 | 0.41 | 3.1 | 0.21 | 84 | 13 | 73 | 4.6 | 22 | 4.6 | 2.3 | 1.5 | 3.7 | 15 | 6.0 | 0.59 |
| M-37 | 0.08 | 6.5 | 7.8 | 380 | 2.0 | 0.34 | 3.2 | 0.18 | 95 | 13 | 77 | 4.1 | 20 | 5.0 | 2.5 | 1.6 | 3.9 | 15 | 6.7 | 0.58 |
| M-38 | 0.12 | 7.1 | 12 | 460 | 2.0 | 0.58 | 2.7 | 0.29 | 81 | 15 | 85 | 5.7 | 30 | 4.5 | 2.2 | 1.5 | 4.0 | 17 | 5.8 | 0.59 |
| M-39 | 0.13 | 7.7 | 15 | 490 | 2.0 | 0.55 | 2.6 | 0.32 | 80 | 17 | 90 | 6.4 | 33 | 4.6 | 2.3 | 1.5 | 4.3 | 18 | 5.9 | 0.61 |
| M-40 | 0.10 | 6.6 | 8.7 | 380 | 2.0 | 0.37 | 2.9 | 0.23 | 96 | 13 | 80 | 4.2 | 21 | 5.1 | 2.6 | 1.6 | 3.8 | 15 | 6.8 | 0.57 |
| T-01 | 0.20 | 8.1 | 220 | 720 | 3.0 | 0.58 | 5.3 | 0.26 | 120 | 20 | 89 | 7.6 | 67 | 6.4 | 2.9 | 2.2 | 4.1 | 21 | 8.9 | 0.06 |
| T-02 | 0.12 | 7.8 | 12 | 665 | 2.0 | 1.2 | 3.5 | 0.19 | 120 | 19 | 110 | 3.5 | 31 | 7.9 | 4.0 | 2.8 | 4.6 | 23 | 9.7 | 0.13 |
| T-03 | 0.09 | 5.6 | 13 | 200 | 1.0 | 0.27 | 9.7 | 0.14 | 210 | 21 | 120 | 4.1 | 51 | 7.0 | 2.7 | 3.0 | 4.2 | 13 | 12 | 0.13 |
| T-04 | 0.05 | 3.8 | 15 | 200 | 0.50 | 0.15 | 12 | 0.19 | 51 | 8.8 | 40 | 4.6 | 17 | 2.8 | 1.4 | 0.90 | 2.3 | 8.5 | 3.9 | 0.15 |
| T-05 | 0.06 | 7.9 | 18 | 400 | 2.0 | 0.30 | 3.6 | 0.27 | 140 | 23 | 90 | 3.7 | 37 | 10 | 5.7 | 2.6 | 8.0 | 17 | 11 | 0.32 |
| T-06 | 0.03 | 7.8 | 2.8 | 320 | 2.0 | 0.28 | 3.7 | 0.20 | 150 | 20 | 79 | 3.4 | 24 | 9.1 | 4.8 | 2.7 | 6.7 | 16 | 11 | 0.30 |
| T-07 | 0.10 | 7.8 | 7.7 | 440 | 3.0 | 0.36 | 3.5 | 0.38 | 130 | 18 | 110 | 4.7 | 31 | 8.8 | 4.6 | 2.7 | 6.3 | 18 | 11 | 0.47 |
| T-08 | 0.04 | 7.7 | 3.7 | 290 | 2.0 | 0.37 | 3.1 | 0.27 | 320 | 17 | 150 | 3.0 | 22 | 14 | 6.5 | 4.2 | 7.3 | 15 | 20 | 0.61 |
| T-09 | 0.04 | 5.2 | 5.3 | 260 | 1.0 | 0.14 | 1.9 | 0.13 | 58 | 57 | 1900 | 2.0 | 39 | 4.1 | 2.1 | 1.3 | 5.7 | 12 | 5.0 | 0.28 |
| T-10 | 0.04 | 5.7 | 12 | 300 | 1.0 | 0.17 | 2.4 | 0.19 | 68 | 39 | 800 | 1.8 | 31 | 6.4 | 3.8 | 1.6 | 5.0 | 13 | 7.0 | 0.55 |
| T-11 | 0.06 | 6.7 | 11 | 370 | 2.0 | 0.25 | 2.2 | 0.14 | 82 | 11 | 73 | 4.1 | 76 | 4.6 | 2.2 | 1.5 | 3.4 | 15 | 6.2 | 1.0 |
| T-12 | 0.03 | 6.9 | 4.1 | 320 | 1.0 | 0.12 | 4.3 | 0.22 | 79 | 17 | 140 | 2.8 | 20 | 7.4 | 4.3 | 2.2 | 4.6 | 16 | 8.6 | 0.43 |
| T-13 | 0.18 | 4.9 | 14 | 320 | 1.0 | 0.37 | 7.9 | 0.17 | 80 | 17 | 130 | 3.2 | 27 | 3.8 | 1.4 | 1.3 | 4.5 | 11 | 4.8 | 1.3 |
| T-14 | 0.09 | 6.1 | 16 | 360 | 2.0 | 0.30 | 6.5 | 0.33 | 71 | 24 | 235 | 5.2 | 41 | 3.8 | 2.0 | 1.3 | 3.4 | 14 | 5.2 | 0.85 |
| T-15 | 0.23 | 7.2 | 70 | 420 | 2.0 | 0.23 | 4.6 | 0.22 | 120 | 21 | 150 | 4.7 | 36 | 5.7 | 2.8 | 2.1 | 4.8 | 18 | 8.4 | 1.3 |
| T-16 | 0.03 | 7.0 | 11 | 300 | 1.0 | 0.15 | 4.5 | 0.23 | 44 | 20 | 80 | 4.9 | 29 | 7.4 | 4.6 | 1.5 | 5.4 | 17 | 6.8 | 0.50 |
| T-17 | 0.05 | 6.0 | 10 | 360 | 2.0 | 0.15 | 6.0 | 0.19 | 82 | 15 | 70 | 4.1 | 22 | 4.0 | 1.7 | 1.7 | 3.7 | 14 | 5.8 | 0.79 |
| T-18 | nd | nd | nd | nd | nd | nd | nd | nd | nd | nd | nd | nd | nd | nd | nd | nd | nd | nd | nd | nd |
| T-19 | 0.08 | 7.1 | 10 | 490 | 2.0 | 0.34 | 1.5 | 0.17 | 77 | 16 | 79 | 4.8 | 30 | 4.2 | 2.1 | 1.4 | 3.9 | 18 | 5.6 | 0.73 |
| T-20 | 0.06 | 7.1 | 3.4 | 485 | 2.0 | 0.30 | 1.3 | 0.18 | 130 | 15 | 82 | 5.2 | 24 | 5.6 | 2.4 | 1.7 | 4.0 | 18 | 8.5 | 0.52 |
| T-21 | 0.08 | 7.8 | 14 | 570 | 3.0 | 0.40 | 1.0 | 0.23 | 79 | 17 | 93 | 6.7 | 35 | 4.0 | 2.0 | 1.4 | 4.4 | 21 | 5.5 | 1.0 |
| T-22 | 0.46 | 6.9 | 8.4 | 525 | 3.0 | 1.0 | 1.1 | 0.38 | 74 | 13 | 80 | 7.9 | 54 | 3.9 | 2.0 | 1.3 | 3.8 | 18 | 5.3 | 0.75 |
| T-23 | 0.14 | 7.5 | 12 | 540 | 2.0 | 0.42 | 1.3 | 0.31 | 73 | 18 | 94 | 6.4 | 36 | 4.2 | 2.1 | 1.4 | 4.5 | 20 | 5.4 | 0.70 |
| T-24 | 0.11 | 7.4 | 17 | 500 | 2.0 | 0.36 | 0.91 | 0.25 | 71 | 15 | 110 | 5.5 | 32 | 3.6 | 1.8 | 1.3 | 4.9 | 19 | 4.9 | 0.76 |
| T-25 | 0.34 | 7.2 | 12 | 580 | 2.0 | 0.46 | 2.1 | 0.50 | 61 | 16 | 82 | 6.5 | 43 | 4.0 | 2.0 | 1.2 | 4.3 | 18 | 5.0 | 0.67 |

Table S4: continued

|  | Ho | In | K | La | Li | Lu | Mg | Mn | Mo | Na | Nb | Nd | Ni | P | Pb | Pr | Rb | Sb | Sc | Se |
| --- | --- | --- | --- | --- | --- | --- | --- | --- | --- | --- | --- | --- | --- | --- | --- | --- | --- | --- | --- | --- |
| unit | mg/kg | mg/kg | % | mg/kg | mg/kg | mg/kg | % | % | mg/kg | % | mg/kg | mg/kg | mg/kg | % | mg/kg | mg/kg | mg/kg | mg/kg | mg/kg | mg/kg |
| LOD | 0.1 | 0.01 | 0.01 | 0.1 | 0.1 | 0.1 | 0.01 | 1 | 0.05 | 0.001 | 0.04 | 0.1 | 0.1 | 0.001 | 0.02 | 0.1 | 0.1 | 0.02 | 0.1 | 0.002 |
| M-00 | 1.2 | 0.08 | 2.4 | 66 | 52 | 0.30 | 1.8 | 0.15 | 0.83 | 1.7 | 7.9 | 61 | 81 | 0.15 | 29 | 16 | 110 | 0.86 | 26 | 0.60 |
| M-01 | 1.1 | 0.08 | 2.8 | 44 | 49 | 0.30 | 2.1 | 0.15 | 0.95 | 1.5 | 7.4 | 42 | 75 | 0.13 | 45 | 11 | 120 | 0.79 | 27 | 0.60 |
| M-02 | 2.1 | 0.11 | 0.70 | 50 | 8.9 | 0.80 | 0.92 | 0.09 | 0.36 | 3.6 | 6.8 | 55 | 15 | 0.21 | 28 | 14 | 24 | 0.34 | 33 | 0.15 |
| M-03 | 1.4 | 0.08 | 1.1 | 83 | 29 | 0.30 | 3.1 | 0.09 | 0.67 | 1.4 | 4.4 | 76 | 73 | 0.14 | 16 | 20 | 53 | 0.94 | 25 | 0.50 |
| M-04 | 1.3 | 0.07 | 1.4 | 61 | 37 | 0.40 | 3.6 | 0.10 | 0.84 | 1.4 | 8.6 | 55 | 57 | 0.13 | 22 | 15 | 68 | 1.4 | 21 | 0.70 |
| M-05 | 1.2 | 0.07 | 1.6 | 61 | 34 | 0.30 | 2.3 | 0.09 | 0.68 | 1.8 | 7.0 | 55 | 56 | 0.14 | 22 | 15 | 73 | 2.2 | 20 | 0.50 |
| M-06 | 1.6 | 0.06 | 1.1 | 88 | 27 | 0.50 | 2.1 | 0.12 | 0.83 | 1.6 | 14 | 81 | 47 | 0.19 | 30 | 21 | 52 | 1.3 | 23 | 0.15 |
| M-07 | 2.0 | 0.07 | 0.92 | 120 | 25 | 0.60 | 2.2 | 0.16 | 0.97 | 1.4 | 17 | 110 | 47 | 0.21 | 35 | 29 | 45 | 1.1 | 27 | 0.40 |
| M-08 | 1.0 | 0.06 | 1.6 | 51 | 39 | 0.30 | 2.2 | 0.09 | 0.65 | 1.6 | 8.1 | 46 | 52 | 0.13 | 23 | 12 | 75 | 1.2 | 18 | 0.15 |
| M-09 | 1.1 | 0.06 | 1.4 | 48 | 34 | 0.40 | 1.9 | 0.09 | 1.7 | 1.7 | 10 | 43 | 37 | 0.17 | 23 | 12 | 66 | 1.1 | 17 | 0.15 |
| M-10 | 1.6 | 0.07 | 1.4 | 79 | 36 | 0.50 | 2.0 | 0.13 | 0.73 | 1.5 | 17 | 74 | 39 | 0.20 | 25 | 19 | 68 | 1.3 | 22 | 0.45 |
| M-11 | 1.0 | 0.06 | 2.0 | 54 | 46 | 0.30 | 2.1 | 0.09 | 0.79 | 1.6 | 9.9 | 47 | 50 | 0.14 | 28 | 13 | 92 | 1.1 | 18 | 0.15 |
| M-12 | 1.1 | 0.06 | 1.8 | 58 | 46 | 0.40 | 1.9 | 0.10 | 0.87 | 1.6 | 12 | 52 | 43 | 0.16 | 26 | 14 | 87 | 1.3 | 18 | 0.30 |
| M-13 | 1.2 | 0.07 | 1.9 | 61 | 47 | 0.40 | 2.0 | 0.12 | 0.87 | 1.6 | 14 | 54 | 49 | 0.17 | 27 | 15 | 95 | 1.1 | 20 | 0.40 |
| M-14 | 1.1 | 0.07 | 1.8 | 55 | 43 | 0.40 | 1.9 | 0.11 | 0.69 | 1.7 | 14 | 51 | 47 | 0.17 | 25 | 13 | 84 | 1.1 | 19 | 0.40 |
| M-15 | 1.9 | 0.08 | 1.3 | 98 | 34 | 0.70 | 2.0 | 0.19 | 1.4 | 1.5 | 23 | 91 | 54 | 0.24 | 26 | 23 | 62 | 1.0 | 24 | 1.10 |
| M-16 | 1.2 | 0.07 | 2.4 | 43 | 46 | 0.40 | 2.2 | 0.08 | 0.84 | 1.5 | 15 | 39 | 71 | 0.14 | 32 | 11 | 85 | 1.3 | 18 | 0.60 |
| M-17 | 1.2 | 0.08 | 1.8 | 58 | 44 | 0.40 | 2.0 | 0.13 | 1.0 | 1.6 | 14 | 53 | 57 | 0.17 | 29 | 14 | 86 | 1.2 | 19 | 0.70 |
| M-18 | 1.0 | 0.07 | 1.9 | 49 | 47 | 0.30 | 2.0 | 0.11 | 1.1 | 1.7 | 12 | 44 | 58 | 0.14 | 27 | 12 | 91 | 1.4 | 18 | 0.45 |
| M-19 | 1.3 | 0.08 | 1.6 | 63 | 40 | 0.50 | 2.0 | 0.14 | 1.6 | 1.6 | 16 | 59 | 60 | 0.18 | 29 | 16 | 81 | 1.2 | 20 | 0.40 |
| M-20 | 0.80 | 0.08 | 2.5 | 44 | 53 | 0.30 | 2.1 | 0.08 | 2.0 | 1.4 | 9.7 | 39 | 68 | 0.11 | 35 | 11 | 120 | 1.8 | 17 | 0.60 |
| M-21 | 1.4 | 0.09 | 1.7 | 50 | 42 | 0.50 | 2.4 | 0.13 | 3.2 | 1.7 | 17 | 48 | 68 | 0.15 | 27 | 12 | 83 | 1.5 | 22 | 0.70 |
| M-22 | 1.1 | 0.08 | 1.7 | 49 | 41 | 0.40 | 2.4 | 0.12 | 3.5 | 1.8 | 14 | 45 | 66 | 0.15 | 34 | 12 | 83 | 1.4 | 18 | 0.50 |
| M-23 | 1.0 | 0.08 | 1.6 | 46 | 41 | 0.30 | 2.4 | 0.12 | 3.2 | 1.6 | 13 | 42 | 61 | 0.12 | 44 | 12 | 80 | 1.6 | 17 | 0.80 |
| M-24 | 1.0 | 0.09 | 1.6 | 43 | 36 | 0.35 | 2.7 | 0.13 | 2.4 | 1.5 | 11 | 40 | 53 | 0.13 | 46 | 10 | 78 | 1.7 | 15 | 0.60 |
| M-25 | 1.1 | 0.08 | 1.7 | 45 | 41 | 0.40 | 2.3 | 0.10 | 2.1 | 1.5 | 12 | 42 | 55 | 0.14 | 34 | 11 | 84 | 1.3 | 17 | 0.55 |
| M-26 | 0.75 | 0.09 | 1.6 | 39 | 33 | 0.30 | 1.8 | 0.09 | 1.6 | 1.6 | 11 | 36 | 40 | 0.10 | 25 | 10 | 80 | 1.2 | 13 | 0.75 |
| M-27 | 1.0 | 0.09 | 1.6 | 46 | 40 | 0.40 | 2.2 | 0.11 | 2.4 | 1.7 | 13 | 42 | 50 | 0.14 | 34 | 11 | 81 | 1.4 | 16 | 0.60 |
| M-28 | 1.1 | 0.06 | 1.7 | 41 | 34 | 0.40 | 1.8 | 0.09 | 1.8 | 1.4 | 10 | 37 | 48 | 0.11 | 30 | 10 | 66 | 1.6 | 14 | 0.30 |
| M-29 | 0.90 | 0.08 | 1.6 | 44 | 37 | 0.30 | 1.6 | 0.09 | 1.3 | 1.6 | 12 | 41 | 41 | 0.12 | 28 | 11 | 81 | 1.1 | 15 | 0.40 |
| M-30 | 0.90 | 0.07 | 1.5 | 50 | 36 | 0.30 | 1.7 | 0.10 | 2.0 | 1.4 | 13 | 45 | 40 | 0.11 | 25 | 12 | 78 | 1.2 | 14 | 0.40 |
| M-31 | 0.80 | 0.07 | 1.6 | 44 | 42 | 0.30 | 1.8 | 0.09 | 1.6 | 1.4 | 12 | 40 | 44 | 0.10 | 27 | 11 | 81 | 1.2 | 15 | 0.40 |
| M-32 | 0.90 | 0.07 | 1.7 | 45 | 41 | 0.30 | 1.7 | 0.10 | 1.7 | 1.4 | 12 | 41 | 43 | 0.11 | 28 | 11 | 85 | 1.1 | 15 | 0.40 |
| M-33 | 0.83 | 0.07 | 1.6 | 43 | 38 | 0.30 | 1.7 | 0.11 | 1.6 | 1.5 | 12 | 39 | 41 | 0.11 | 25 | 10 | 79 | 1.2 | 14 | 0.43 |
| M-34 | 1.2 | 0.07 | 1.6 | 49 | 46 | 0.40 | 1.7 | 0.12 | 1.7 | 1.4 | 15 | 44 | 40 | 0.12 | 29 | 12 | 85 | 1.6 | 15 | 0.30 |
| M-35 | 0.90 | 0.07 | 1.6 | 45 | 37 | 0.30 | 1.7 | 0.11 | 1.6 | 1.4 | 12 | 41 | 40 | 0.11 | 25 | 11 | 79 | 1.1 | 14 | 0.70 |
| M-36 | 0.80 | 0.07 | 1.6 | 43 | 41 | 0.30 | 1.6 | 0.10 | 1.4 | 1.4 | 12 | 38 | 39 | 0.11 | 26 | 11 | 79 | 1.1 | 14 | 0.15 |
| M-37 | 0.90 | 0.07 | 1.4 | 48 | 35 | 0.30 | 1.6 | 0.11 | 1.4 | 1.5 | 13 | 43 | 37 | 0.11 | 25 | 12 | 76 | 1.2 | 15 | 0.30 |
| M-38 | 0.80 | 0.09 | 1.7 | 40 | 45 | 0.30 | 1.6 | 0.11 | 1.6 | 1.3 | 10 | 37 | 46 | 0.12 | 30 | 10 | 96 | 1.4 | 14 | 0.50 |
| M-39 | 0.80 | 0.09 | 2.0 | 39 | 49 | 0.30 | 1.6 | 0.12 | 1.5 | 1.3 | 10 | 36 | 49 | 0.12 | 35 | 10 | 100 | 1.5 | 15 | 0.60 |
| M-40 | 0.90 | 0.08 | 1.5 | 47 | 36 | 0.30 | 1.6 | 0.11 | 1.7 | 1.5 | 12 | 42 | 41 | 0.11 | 27 | 12 | 75 | 1.1 | 14 | 0.15 |
| T-01 | 1.1 | 0.08 | 2.9 | 59 | 44 | 0.30 | 2.2 | 0.10 | 0.94 | 1.4 | 9.6 | 56 | 86 | 0.13 | 31 | 15 | 105 | 0.53 | 15 | 1.1 |
| T-02 | 1.4 | 0.10 | 1.7 | 53 | 18 | 0.50 | 1.7 | 0.10 | 0.90 | 1.6 | 8.2 | 60 | 53 | 0.18 | 38 | 15 | 51 | 0.85 | 29 | 0.80 |
| T-03 | 1.1 | 0.06 | 1.2 | 100 | 33 | 0.20 | 3.5 | 0.10 | 0.83 | 0.65 | 3.6 | 94 | 76 | 0.12 | 15 | 24 | 63 | 0.98 | 19 | 1.2 |
| T-04 | 0.50 | 0.04 | 1.0 | 25 | 28 | 0.20 | 6.6 | 0.06 | 1.2 | 0.69 | 3.9 | 23 | 22 | 0.06 | 17 | 5.7 | 50 | 2.6 | 9 | 0.80 |
| T-05 | 1.9 | 0.07 | 1.5 | 67 | 46 | 0.70 | 1.7 | 0.21 | 2.1 | 1.3 | 22 | 60 | 41 | 0.22 | 17 | 16 | 66 | 0.77 | 26 | 1.2 |
| T-06 | 1.6 | 0.07 | 1.4 | 71 | 41 | 0.60 | 1.7 | 0.16 | 0.59 | 1.4 | 17 | 66 | 31 | 0.19 | 19 | 18 | 51 | 0.28 | 24 | 0.90 |
| T-07 | 1.7 | 0.08 | 1.3 | 64 | 49 | 0.60 | 1.7 | 0.18 | 1.5 | 1.6 | 24 | 61 | 51 | 0.23 | 44 | 16 | 60 | 3.5 | 21 | 1.3 |
| T-08 | 2.3 | 0.07 | 1.0 | 160 | 34 | 0.80 | 1.5 | 0.24 | 0.77 | 1.4 | 23 | 140 | 45 | 0.16 | 35 | 37 | 49 | 0.51 | 26 | 1.2 |
| T-09 | 0.75 | 0.05 | 0.795 | 29 | 18 | 0.30 | 7.4 | 0.12 | 0.44 | 1.3 | 9.8 | 26 | 710 | 0.07 | 15 | 6.7 | 42 | 0.61 | 17 | 0.65 |
| T-10 | 1.3 | 0.06 | 0.78 | 32 | 16 | 0.50 | 5.5 | 0.13 | 0.44 | 1.5 | 9.2 | 34 | 450 | 0.09 | 13 | 7.9 | 36 | 0.79 | 18 | 0.60 |
| T-11 | 0.80 | 0.06 | 1.6 | 39 | 33 | 0.30 | 1.3 | 0.07 | 1.5 | 1.9 | 5.5 | 39 | 43 | 0.13 | 21 | 10 | 70 | 1.2 | 12 | 0.70 |
| T-12 | 1.4 | 0.08 | 0.69 | 36 | 21 | 0.50 | 2.5 | 0.14 | 1.2 | 1.9 | 9.2 | 42 | 71 | 0.11 | 18 | 10 | 35 | 0.73 | 23 | 1.0 |
| T-13 | 0.50 | 0.12 | 1.5 | 41 | 20 | 0.20 | 1.4 | 0.21 | 3.1 | 0.72 | 6.1 | 34 | 77 | 0.12 | 34 | 9.1 | 69 | 2.7 | 9 | 0.90 |
| T-14 | 0.70 | 0.06 | 1.6 | 36 | 33 | 0.30 | 3.6 | 0.07 | 25 | 1.3 | 16 | 33 | 230 | 0.10 | 35 | 8.2 | 85 | 1.7 | 11 | 0.70 |
| T-15 | 1.0 | 0.08 | 1.6 | 60 | 49 | 0.40 | 2.3 | 0.13 | 1.1 | 1.3 | 12 | 56 | 72 | 0.13 | 36 | 15 | 81 | 8.0 | 18 | 0.90 |
| T-16 | 1.5 | 0.08 | 0.73 | 20 | 21 | 0.60 | 2.4 | 0.13 | 1.2 | 2.1 | 7.2 | 24 | 34 | 0.09 | 12 | 5.4 | 35 | 0.95 | 24 | 0.90 |
| T-17 | 0.60 | 0.05 | 1.4 | 40 | 25 | 0.20 | 3.0 | 0.08 | 0.57 | 1.2 | 14 | 38 | 32 | 0.15 | 28 | 9.9 | 63 | 1.1 | 12 | 0.50 |
| T-18 | nd | nd | nd | nd | nd | nd | nd | nd | nd | nd | nd | nd | nd | nd | nd | nd | nd | nd | nd | nd |
| T-19 | 0.70 | 0.06 | 1.9 | 37 | 42 | 0.30 | 1.0 | 0.12 | 0.46 | 0.88 | 9.8 | 35 | 40 | 0.09 | 24 | 9.1 | 90 | 1.6 | 13 | 0.50 |
| T-20 | 0.90 | 0.06 | 1.8 | 63 | 51 | 0.30 | 1.0 | 0.10 | 0.88 | 1.1 | 9.8 | 57 | 37 | 0.09 | 26 | 15 | 91 | 0.60 | 13 | 0.50 |
| T-21 | 0.70 | 0.08 | 2.1 | 39 | 55 | 0.20 | 1.1 | 0.08 | 0.86 | 0.77 | 16 | 35 | 50 | 0.13 | 28 | 9.3 | 115 | 1.3 | 14 | 0.70 |
| T-22 | 0.70 | 0.07 | 1.7 | 37 | 54 | 0.20 | 1.0 | 0.06 | 0.91 | 1.1 | 9.2 | 33 | 40 | 0.22 | 41 | 8.5 | 100 | 1.3 | 13 | 0.60 |
| T-23 | 0.70 | 0.10 | 2.0 | 35 | 54 | 0.30 | 1.4 | 0.09 | 1.1 | 0.86 | 9.7 | 32 | 50 | 0.12 | 33 | 8.1 | 110 | 1.4 | 14 | 0.80 |
| T-24 | 0.60 | 0.07 | 1.9 | 34 | 47 | 0.20 | 1.0 | 0.14 | 0.53 | 0.95 | 9.2 | 31 | 39 | 0.19 | 26 | 8.0 | 100 | 1.0 | 13 | 0.50 |
| T-25 | 0.70 | 0.07 | 1.8 | 28 | 49 | 0.30 | 0.93 | 0.22 | 0.62 | 0.79 | 8.7 | 28 | 43 | 0.24 | 34 | 7.0 | 86 | 1.5 | 13 | 0.70 |

**Table S4:** continued

|  | Sm | Sn | Sr | Ta | Tb | Te | Th | Ti | Tl | Tm | U | V | W | Y | Yb | Zn | Zr | Eu/Eu* | Ce/Ce* | Gd/Gd* |
| --- | --- | --- | --- | --- | --- | --- | --- | --- | --- | --- | --- | --- | --- | --- | --- | --- | --- | --- | --- | --- |
| unit | **mg/kg** | **mg/kg** | **mg/kg** | **mg/kg** | **mg/kg** | **mg/kg** | **mg/kg** | **%** | **mg/kg** | **mg/kg** | **mg/kg** | **mg/kg** | **mg/kg** | **mg/kg** | **mg/kg** | **mg/kg** | **mg/kg** |  |  |  |
| LOD | **0.1** | **0.1** | **1** | **0.1** | **0.1** | **0.05** | **0.1** | **0.001** | **0.05** | **0.1** | **0.1** | **2** | **0.1** | **0.1** | **0.1** | **0.2** | **0.2** |  |  |  |
| M-00 | 12 | 2.8 | 270 | 0.60 | 1.3 | 0.12 | 17 | 0.53 | 0.69 | 0.40 | 3.3 | 170 | 1.9 | 33 | 2.4 | 100 | 2.0 | 1.0 | 0.96 | 0.89 |
| M-01 | 8.3 | 2.7 | 340 | 0.50 | 1.0 | 0.16 | 13 | 0.49 | 0.66 | 0.40 | 3.1 | 160 | 2.6 | 32 | 2.6 | 110 | 2.0 | 1.1 | 0.95 | 0.83 |
| M-02 | 12 | 2.5 | 900 | 0.60 | 1.8 | 0.13 | 13 | 0.50 | 0.14 | 0.90 | 4.8 | 130 | 2.7 | 61 | 5.6 | 32 | 2.0 | 1.2 | 1.0 | 0.73 |
| M-03 | 15 | 1.9 | 370 | 0.30 | 1.6 | 0.11 | 19 | 0.47 | 0.03 | 0.50 | 2.7 | 140 | 0.50 | 38 | 2.8 | 62 | 2.0 | 0.98 | 1.0 | 0.90 |
| M-04 | 11 | 2.1 | 260 | 0.70 | 1.2 | 0.07 | 15 | 0.57 | 0.37 | 0.50 | 2.5 | 130 | 1.0 | 34 | 2.9 | 110 | 4.0 | 0.95 | 0.98 | 0.95 |
| M-05 | 11 | 2.2 | 290 | 0.50 | 1.2 | 0.09 | 16 | 0.49 | 0.39 | 0.40 | 2.5 | 120 | 0.90 | 32 | 2.6 | 83 | 4.8 | 1.0 | 1.05 | 0.92 |
| M-06 | 15 | 1.7 | 270 | 1.1 | 1.6 | 0.10 | 22 | 0.76 | 0.25 | 0.60 | 3.3 | 110 | 1.3 | 45 | 3.8 | 72 | 7.3 | 1.0 | 1.1 | 0.94 |
| M-07 | 20 | 2.0 | 280 | 1.3 | 2.0 | 0.11 | 32 | 0.92 | 0.23 | 0.70 | 4.5 | 120 | 2.6 | 55 | 4.8 | 67 | 10 | 0.98 | 1.1 | 0.94 |
| M-08 | 9.0 | 2.1 | 230 | 0.60 | 1.0 | 0.08 | 14 | 0.51 | 0.38 | 0.40 | 2.3 | 110 | 0.90 | 27 | 2.3 | 83 | 7.3 | 1.0 | 1.0 | 0.90 |
| M-09 | 8.5 | 1.9 | 230 | 0.70 | 1.0 | 0.06 | 13 | 0.54 | 0.36 | 0.40 | 2.4 | 98 | 1.2 | 30 | 2.6 | 73 | 8.2 | 1.1 | 1.0 | 0.85 |
| M-10 | 13 | 2.3 | 230 | 1.3 | 1.5 | 0.08 | 20 | 0.82 | 0.36 | 0.60 | 3.6 | 115 | 2.0 | 43 | 4.0 | 87 | 14 | 1.0 | 1.0 | 0.92 |
| M-11 | 9.2 | 2.5 | 210 | 0.70 | 1.0 | 0.07 | 14 | 0.52 | 0.49 | 0.40 | 2.6 | 120 | 1.3 | 28 | 2.5 | 93 | 8.3 | 1.0 | 0.99 | 0.85 |
| M-12 | 10 | 2.5 | 220 | 0.90 | 1.1 | 0.05 | 15 | 0.59 | 0.44 | 0.40 | 2.8 | 120 | 1.3 | 31 | 2.7 | 91 | 10 | 1.0 | 0.98 | 0.90 |
| M-13 | 10 | 3.8 | 220 | 1.0 | 1.2 | 0.11 | 16 | 0.65 | 0.23 | 0.40 | 2.9 | 130 | 1.2 | 32 | 2.9 | 100 | 12 | 1.05 | 0.94 | 0.88 |
| M-14 | 9.8 | 2.8 | 230 | 1.0 | 1.1 | 0.06 | 14 | 0.62 | 0.42 | 0.40 | 2.8 | 120 | 1.2 | 33 | 2.8 | 96 | 13 | 1.0 | 1.0 | 0.87 |
| M-15 | 17 | 2.8 | 250 | 1.8 | 1.8 | 0.07 | 28 | 1.1 | 0.31 | 0.70 | 4.7 | 120 | 4.4 | 53 | 5.0 | 86 | 22 | 0.97 | 1.1 | 0.99 |
| M-16 | 7.7 | 3.4 | 195 | 1.0 | 1.2 | 0.03 | 12 | 0.48 | 0.59 | 0.50 | 3.1 | 125 | 1.5 | 25 | 3.0 | 150 | 14 | 0.99 | 0.99 | 0.92 |
| M-17 | 10 | 4.2 | 230 | 1.05 | 1.2 | 0.07 | 15 | 0.69 | 0.45 | 0.50 | 3.1 | 120 | 1.3 | 34 | 3.1 | 105 | 15 | 0.99 | 1.0 | 0.93 |
| M-18 | 8.7 | 3.1 | 210 | 0.80 | 1.0 | 0.07 | 13 | 0.56 | 0.48 | 0.40 | 2.7 | 120 | 1.3 | 27 | 2.4 | 105 | 14 | 1.0 | 0.98 | 0.89 |
| M-19 | 11 | 4.9 | 230 | 1.1 | 1.3 | 0.06 | 16 | 0.74 | 0.41 | 0.50 | 3.0 | 125 | 1.6 | 37 | 3.4 | 100 | 18 | 1.05 | 1.0 | 0.89 |
| M-20 | 7.8 | 3.2 | 190 | 0.60 | 0.90 | 0.07 | 12 | 0.45 | 0.62 | 0.30 | 2.7 | 130 | 2.0 | 23 | 2.1 | 140 | 21 | 0.98 | 0.98 | 0.92 |
| M-21 | 10 | 3.5 | 200 | 1.1 | 1.2 | 0.03 | 12 | 0.88 | 0.51 | 0.50 | 3.2 | 140 | 2.4 | 38 | 3.6 | 110 | 23 | 1.0 | 1.1 | 0.88 |
| M-22 | 9.1 | 3.7 | 210 | 0.90 | 1.1 | 0.06 | 12 | 0.64 | 0.45 | 0.40 | 2.7 | 130 | 2.4 | 30 | 2.8 | 120 | 21 | 0.99 | 0.96 | 0.90 |
| M-23 | 8.3 | 7.0 | 200 | 0.80 | 0.90 | 0.05 | 12 | 0.56 | 0.42 | 0.40 | 2.6 | 110 | 2.3 | 27 | 2.5 | 135 | 23 | 0.98 | 0.94 | 0.94 |
| M-24 | 7.9 | 4.1 | 210 | 0.80 | 0.95 | 0.05 | 11 | 0.53 | 0.47 | 0.40 | 2.5 | 110 | 2.6 | 27 | 2.5 | 180 | 21 | 0.99 | 1.0 | 0.90 |
| M-25 | 8.3 | 3.7 | 205 | 0.85 | 1.0 | 0.04 | 12 | 0.54 | 0.48 | 0.40 | 2.7 | 110 | 2.1 | 29 | 2.7 | 130 | 19 | 1.0 | 0.98 | 0.91 |
| M-26 | 7.1 | 3.2 | 180 | 0.80 | 0.75 | 0.05 | 10 | 0.50 | 0.42 | 0.30 | 2.1 | 94 | 2.2 | 22 | 2.0 | 98 | 20 | 1.0 | 1.0 | 0.88 |
| M-27 | 8.2 | 4.6 | 210 | 1.0 | 1.0 | 0.03 | 11 | 0.59 | 0.43 | 0.40 | 2.6 | 110 | 3.2 | 29 | 2.6 | 120 | 24 | 1.05 | 1.0 | 0.87 |
| M-28 | 7.3 | 3.4 | 170 | 0.70 | 1.0 | 0.06 | 10 | 0.50 | 0.48 | 0.40 | 2.2 | 100 | 2.4 | 22 | 2.6 | 140 | 20 | 1.05 | 0.98 | 0.85 |
| M-29 | 7.8 | 7.3 | 190 | 0.80 | 0.90 | 0.03 | 11 | 0.51 | 0.40 | 0.30 | 2.2 | 100 | 3.1 | 26 | 2.2 | 100 | 17 | 0.99 | 0.93 | 0.92 |
| M-30 | 8.6 | 3.1 | 170 | 0.90 | 0.90 | 0.03 | 13 | 0.55 | 0.44 | 0.30 | 2.5 | 92 | 3.6 | 25 | 2.2 | 100 | 21 | 0.96 | 0.97 | 0.95 |
| M-31 | 7.6 | 3.2 | 180 | 0.80 | 0.80 | 0.03 | 11 | 0.49 | 0.44 | 0.30 | 2.3 | 99 | 3.0 | 23 | 2.0 | 110 | 20 | 0.96 | 1.0 | 0.97 |
| M-32 | 8.0 | 3.3 | 180 | 0.80 | 0.90 | 0.06 | 12 | 0.50 | 0.45 | 0.30 | 2.5 | 100 | 3.4 | 24 | 2.1 | 110 | 22 | 0.96 | 0.96 | 0.94 |
| M-33 | 7.5 | 3.5 | 180 | 0.83 | 0.83 | 0.03 | 11 | 0.52 | 0.43 | 0.30 | 2.4 | 97 | 2.9 | 23 | 2.1 | 100 | 21 | 0.98 | 1.0 | 0.93 |
| M-34 | 8.6 | 3.1 | 180 | 1.0 | 1.2 | 0.06 | 13 | 0.56 | 0.42 | 0.50 | 2.5 | 98 | 3.3 | 26 | 3.0 | 110 | 22 | 0.95 | 0.97 | 0.95 |
| M-35 | 7.9 | 3.5 | 180 | 0.90 | 0.85 | 0.05 | 12 | 0.55 | 0.43 | 0.30 | 2.4 | 97 | 3.3 | 25 | 2.2 | 100 | 21 | 0.98 | 0.98 | 0.93 |
| M-36 | 7.5 | 3.9 | 170 | 0.80 | 0.80 | 0.03 | 11 | 0.52 | 0.42 | 0.30 | 2.3 | 96 | 2.8 | 22 | 2.0 | 100 | 22 | 0.96 | 0.92 | 0.92 |
| M-37 | 8.2 | 2.9 | 180 | 0.90 | 0.90 | 0.03 | 12 | 0.53 | 0.39 | 0.30 | 2.4 | 93 | 2.4 | 26 | 2.3 | 98 | 21 | 0.94 | 0.94 | 0.98 |
| M-38 | 7.1 | 4.4 | 160 | 0.70 | 0.80 | 0.06 | 11 | 0.44 | 0.51 | 0.30 | 2.2 | 100 | 3.6 | 22 | 2.0 | 130 | 22 | 1.0 | 0.95 | 0.88 |
| M-39 | 7.1 | 3.4 | 170 | 0.70 | 0.80 | 0.05 | 11 | 0.44 | 0.57 | 0.30 | 2.2 | 110 | 3.3 | 23 | 2.0 | 140 | 22 | 1.0 | 1.0 | 0.88 |
| M-40 | 8.2 | 4.5 | 170 | 0.80 | 0.90 | 0.05 | 13 | 0.52 | 0.41 | 0.30 | 2.4 | 97 | 3.0 | 26 | 2.3 | 96 | 19 | 0.94 | 0.97 | 0.99 |
| T-01 | 11 | 4.0 | 380 | 0.80 | 1.1 | 0.17 | 20 | 0.27 | 0.83 | 0.40 | 5.0 | 100 | 1.9 | 31 | 2.3 | 89 | 1.3 | 0.97 | 1.0 | 0.93 |
| T-02 | 12 | 2.7 | 720 | 0.70 | 1.3 | 0.52 | 11 | 0.55 | 0.44 | 0.60 | 3.2 | 160 | 2.2 | 39 | 3.5 | 72 | 1.9 | 1.15 | 1.1 | 0.75 |
| T-03 | 17 | 1.7 | 235 | 0.30 | 1.4 | 0.11 | 21 | 0.40 | 0.34 | 0.30 | 1.9 | 130 | 2.5 | 28 | 1.8 | 70 | 3.0 | 0.90 | 1.1 | 0.94 |
| T-04 | 4.5 | 1.4 | 130 | 0.30 | 0.50 | 0.03 | 5.9 | 0.25 | 0.29 | 0.20 | 2.1 | 65 | 0.80 | 14 | 1.3 | 63 | 4.3 | 0.95 | 1.1 | 0.99 |
| T-05 | 12 | 2.4 | 250 | 1.6 | 1.6 | 0.07 | 17 | 1.0 | 0.37 | 0.80 | 3.3 | 140 | 1.2 | 54 | 5.4 | 100 | 9.5 | 1.1 | 1.0 | 0.90 |
| T-06 | 13 | 2.2 | 250 | 1.2 | 1.5 | 0.09 | 20 | 0.73 | 0.34 | 0.60 | 3.5 | 140 | 1.0 | 45 | 4.2 | 80 | 9.0 | 1.0 | 0.98 | 0.90 |
| T-07 | 12 | 7.0 | 290 | 1.8 | 1.5 | 0.09 | 17 | 1.1 | 0.37 | 0.60 | 3.7 | 140 | 1.3 | 44 | 4.0 | 120 | 15 | 1.05 | 1.05 | 0.88 |
| T-08 | 25 | 6.3 | 230 | 2.0 | 2.5 | 0.08 | 48 | 1.0 | 0.29 | 0.90 | 8.5 | 110 | 1.3 | 62 | 5.7 | 84 | 18 | 0.85 | 1.1 | 1.2 |
| T-09 | 5.5 | 1.7 | 160 | 0.70 | 0.70 | 0.04 | 6.4 | 0.58 | 0.26 | 0.30 | 1.4 | 120 | 0.70 | 21 | 1.9 | 97 | 8.4 | 1.1 | 1.1 | 0.82 |
| T-10 | 7.5 | 2.1 | 160 | 1.0 | 1.0 | 0.03 | 7.1 | 0.62 | 0.23 | 0.50 | 2.3 | 110 | 0.90 | 35 | 3.7 | 85 | 11 | 0.96 | 1.2 | 0.95 |
| T-11 | 7.7 | 5.4 | 140 | 0.50 | 0.80 | 0.06 | 9.5 | 0.36 | 0.40 | 0.30 | 2.8 | 84 | 0.90 | 21 | 1.8 | 85 | 32 | 0.92 | 1.05 | 0.95 |
| T-12 | 9.1 | 2.0 | 320 | 0.70 | 1.2 | 0.05 | 6.4 | 0.76 | 0.24 | 0.60 | 2.2 | 120 | 0.50 | 40 | 3.9 | 90 | 11 | 1.1 | 1.1 | 0.81 |
| T-13 | 6.5 | 2.7 | 92 | 0.40 | 0.50 | 0.10 | 8.1 | 0.26 | 0.43 | 0.20 | 2.1 | 95 | 1.5 | 14 | 1.3 | 95 | 46 | 0.98 | 1.1 | 0.86 |
| T-14 | 6.5 | 3.5 | 150 | 0.80 | 0.60 | 0.06 | 8.5 | 0.48 | 0.46 | 0.30 | 2.1 | 98 | 13 | 20 | 1.8 | 120 | 28 | 0.95 | 1.1 | 0.92 |
| T-15 | 11 | 4.7 | 220 | 0.80 | 1.0 | 0.08 | 13 | 0.58 | 0.46 | 0.40 | 2.3 | 130 | 1.0 | 26 | 2.6 | 105 | 45 | 0.93 | 1.0 | 0.93 |
| T-16 | 6.1 | 2.5 | 170 | 0.50 | 1.1 | 0.07 | 4.1 | 0.79 | 0.32 | 0.70 | 2.3 | 160 | 0.80 | 42 | 4.6 | 100 | 11 | 1.0 | 1.2 | 0.89 |
| T-17 | 7.3 | 1.7 | 150 | 0.90 | 0.70 | 0.03 | 8.3 | 0.64 | 0.41 | 0.20 | 1.4 | 110 | 0.70 | 19 | 1.4 | 85 | 30 | 1.1 | 1.0 | 0.75 |
| T-18 | nd | nd | nd | nd | nd | nd | nd | nd | nd | nd | nd | nd | nd | nd | nd | nd | nd | nd | nd | nd |
| T-19 | 6.8 | 3.4 | 110 | 0.70 | 0.70 | 0.06 | 9.4 | 0.43 | 0.56 | 0.30 | 1.7 | 110 | 1.5 | 19 | 1.8 | 140 | 24 | 0.99 | 1.0 | 0.91 |
| T-20 | 11 | 2.8 | 150 | 1.2 | 1.1 | 0.03 | 19 | 0.43 | 0.57 | 0.30 | 3.6 | 105 | 23 | 24 | 1.9 | 99 | 16 | 0.78 | 1.0 | 1.3 |
| T-21 | 6.9 | 3.4 | 130 | 1.0 | 0.70 | 0.08 | 11 | 0.44 | 0.74 | 0.30 | 2.2 | 120 | 2.2 | 19 | 1.8 | 120 | 41 | 0.98 | 1.0 | 0.89 |
| T-22 | 6.3 | 5 | 140 | 0.70 | 0.70 | 0.07 | 9.6 | 0.39 | 0.68 | 0.30 | 2.1 | 91 | 1.5 | 19 | 1.7 | 200 | 26 | 1.0 | 1.1 | 0.93 |
| T-23 | 6.4 | 3.7 | 120 | 0.80 | 0.70 | 0.06 | 9.8 | 0.38 | 0.68 | 0.30 | 2.4 | 120 | 3.3 | 20 | 1.8 | 130 | 25 | 1.05 | 1.2 | 0.86 |
| T-24 | 6.1 | 3.3 | 110 | 0.70 | 0.60 | 0.06 | 9.9 | 0.37 | 0.64 | 0.30 | 1.8 | 110 | 1.4 | 17 | 1.6 | 120 | 28 | 1.0 | 1.1 | 0.85 |
| T-25 | 5.8 | 4.5 | 140 | 0.60 | 0.70 | 0.03 | 8.7 | 0.36 | 0.62 | 0.30 | 1.8 | 92 | 1.5 | 19 | 1.9 | 180 | 24 | 0.97 | 1.1 | 0.93 |

Table S5: Element levels of 36(M) and 26(T) elements in SPM samples at low water regime (main channel and tributaries)

|  | Ag | Al | As | Ba | Bi | Ca | Cd | Ce | Co | Cr | Cu | Dy | Er | Eu | Fe | Ga | Gd | La | Li | Mg |
| --- | --- | --- | --- | --- | --- | --- | --- | --- | --- | --- | --- | --- | --- | --- | --- | --- | --- | --- | --- | --- |
| LOD | 1 | 1 | 1 | 10 | 1 | 5 | 1 | 1 | 1 | 10 | 5 | 1 | 1 | 1 | 0.05 | 5 | 1 | 1 | 5 | 1 |
| unit | µg/L | mg/L | µg/L | µg/L | µg/L | mg/L | µg/L | µg/L | µg/L | µg/L | µg/L | µg/L | µg/L | µg/L | mg/L | µg/L | µg/L | µg/L | µg/L | mg/L |
| M-00 | 3.3 | 13 | 54 | 380 | 0.73 | 7.4 | 0.73 | 4.8 | 3.2 | 400 | 64 | 0.73 | 0.73 | 0.73 | 0.49 | 68 | 0.73 | 2.8 | 37 | 8.5 |
| M-01 | 2.9 | 0.8 | 47 | 7.3 | 0.73 | 7.4 | 1.4 | 3.4 | 2.0 | 330 | 75 | 0.73 | 0.73 | 0.73 | 0.62 | 3.6 | 0.73 | 1.5 | 3.6 | 9.4 |
| M-02 | 3.1 | 20 | 340 | 240 | 0.73 | 3.6 | 1.6 | 24 | 20 | 390 | 130 | 2.5 | 1.6 | 0.73 | 4.1 | 47 | 2.5 | 13 | 3.6 | 15 |
| M-03 | 2.6 | 4.2 | 130 | 51 | 0.57 | 7.3 | 0.66 | 4.6 | 3.8 | 290 | 72 | 0.67 | 0.57 | 0.57 | 3.1 | 7.3 | 0.64 | 3.2 | 2.9 | 13 |
| M-04 | 3.1 | 26 | 110 | 210 | 0.57 | 7.0 | 0.94 | 46 | 15 | 340 | 100 | 3.1 | 1.6 | 0.82 | 4.7 | 46 | 5.1 | 26 | 8.1 | 20 |
| M-05 | 2.9 | 11 | 120 | 120 | 0.79 | 7.6 | 0.57 | 23 | 8.4 | 310 | 99 | 2.2 | 0.89 | 0.66 | 3.8 | 23 | 3.1 | 14 | 6.0 | 14 |
| M-06 | 3.1 | 11 | 120 | 110 | 0.62 | 9.7 | 0.57 | 21 | 9.7 | 290 | 100 | 1.8 | 1.1 | 0.59 | 3.7 | 22 | 2.4 | 13 | 2.9 | 19 |
| M-07 | 2.5 | 10 | 72 | 93 | 0.57 | 6.6 | 0.80 | 20 | 8.5 | 290 | 100 | 1.6 | 1.0 | 0.57 | 2.6 | 18 | 2.4 | 13 | 2.9 | 14 |
| M-08 | 3.1 | 16 | 96 | 130 | 1.2 | 6.3 | 0.57 | 26 | 9.5 | 340 | 350 | 2.9 | 1.0 | 0.66 | 3.9 | 25 | 2.8 | 16 | 2.9 | 14 |
| M-09 | 2.6 | 18 | 69 | 155 | 1.3 | 6.0 | 0.57 | 30 | 13 | 300 | 175 | 3.5 | 0.94 | 0.59 | 3.4 | 32 | 3.9 | 18 | 14 | 14 |
| M-10 | 2.4 | 24 | 72 | 370 | 1.1 | 6.6 | 0.57 | 24 | 8.9 | 300 | 190 | 3.9 | 0.84 | 0.57 | 3.1 | 67 | 3.5 | 14 | 52 | 14 |
| M-11 | 4.1 | 16 | 62 | 200 | 1.5 | 2.9 | 1.5 | 44 | 14 | 350 | 230 | 4.1 | 1.2 | 0.72 | 3.8 | 42 | 3.9 | 23 | 2.9 | 15 |
| M-12 | 2.7 | 17 | 37 | 130 | 1.5 | 5.7 | 0.94 | 37 | 12 | 310 | 230 | 3.8 | 1.5 | 0.71 | 2.8 | 25 | 4.0 | 20 | 2.9 | 13 |
| M-13 | 2.1 | 60 | 54 | 510 | 2.4 | 7.9 | 2.7 | 120 | 40 | 420 | 270 | 7.2 | 3.3 | 2.0 | 8.6 | 100 | 11 | 58 | 50 | 29 |
| M-14 | 0.88 | 19 | 30 | 180 | 1.0 | 5.7 | 1.2 | 31 | 11 | 340 | 145 | 2.3 | 1.1 | 0.69 | 2.8 | 36 | 2.6 | 17 | 6.0 | 14 |
| M-15 | 1.0 | 25 | 16 | 140 | 1.2 | 8.4 | 0.80 | 25 | 9.6 | 310 | 170 | 2.0 | 0.87 | 0.57 | 2.1 | 26 | 2.5 | 14 | 2.9 | 14 |
| M-16 | 22 | 31 | 53 | 400 | 15 | 12 | 6.4 | 59 | 21 | 470 | 410 | 4.1 | 2.0 | 1.4 | 9.4 | 75 | 5.4 | 34 | 23 | 25 |
| M-17 | 7.4 | 15 | 34 | 170 | 4.6 | 10 | 4.4 | 28 | 9.7 | 340 | 240 | 2.3 | 1.2 | 0.71 | 4.1 | 35 | 2.6 | 16 | 2.9 | 16 |
| M-18 | 12 | 20 | 32 | 240 | 7.7 | 8.5 | 4.4 | 30 | 12 | 405 | 385 | 2.5 | 1.2 | 0.57 | 4.5 | 41 | 3.1 | 17 | 2.9 | 18 |
| M-19 | 13 | 31 | 49 | 340 | 9.3 | 12 | 2.7 | 53 | 65 | 740 | 345 | 3.6 | 2.0 | 1.0 | 6.8 | 67 | 5.2 | 29 | 33 | 25 |
| M-20 | 11 | 35 | 55 | 475 | 7.5 | 10 | 9.6 | 54 | 34 | 510 | 310 | 4.1 | 1.9 | 1.6 | 6.0 | 83 | 5.4 | 29 | 77 | 22 |
| M-21 | 11 | 19 | 42 | 220 | 6.6 | 7.9 | 5.7 | 34 | 24 | 460 | 260 | 2.9 | 1.2 | 0.81 | 4.5 | 43 | 3.6 | 19 | 2.9 | 16 |
| M-22 | 11 | 27 | 50 | 350 | 8.5 | 12 | 7.3 | 48 | 35 | 520 | 265 | 4.3 | 2.2 | 1.1 | 5.7 | 66 | 6.0 | 26 | 8.1 | 21 |
| M-23 | 10 | 140 | 140 | 1100 | 9.3 | 32 | 15 | 190 | 81 | 650 | 430 | 14 | 6.5 | 4.4 | 16 | 230 | 19 | 91 | 100 | 59 |
| M-24 | 7.2 | 34 | 56 | 410 | 7.6 | 13 | 9.2 | 57 | 27 | 510 | 270 | 4.2 | 2.2 | 1.1 | 6.5 | 78 | 6.1 | 29 | 8.1 | 25 |
| M-25 | 9.6 | 260 | 330 | 2400 | 13 | 75 | 9.9 | 540 | 200 | 880 | 1300 | 39 | 21 | 12 | 39 | 490 | 57 | 260 | 295 | 135 |
| M-26 | 1.8 | 100 | 77 | 910 | 5.3 | 14 | 3.4 | 180 | 59 | 550 | 280 | 12 | 6.2 | 3.8 | 19 | 180 | 19 | 86 | 50 | 33 |
| M-27 | 4.3 | 130 | 145 | 1200 | 11 | 24 | 10 | 220 | 80 | 610 | 600 | 17 | 9.0 | 4.9 | 20 | 240 | 26 | 110 | 120 | 59 |
| M-28 | 2.8 | 92 | 97 | 760 | 7.5 | 16 | 7.0 | 140 | 48 | 530 | 330 | 11 | 6.1 | 2.8 | 13 | 150 | 15 | 70 | 52 | 42 |
| M-29 | 5.0 | 83 | 90 | 680 | 9.2 | 17 | 6.4 | 150 | 88 | 570 | 320 | 10 | 5.6 | 3.1 | 16 | 150 | 16 | 77 | 75 | 43 |
| M-30 | 4.2 | 120 | 110 | 910 | 7.5 | 18 | 15 | 170 | 69 | 590 | 550 | 14 | 7.1 | 4.1 | 19 | 180 | 20 | 87 | 150 | 47 |
| M-31 | 4.4 | 110 | 110 | 930 | 8.4 | 20 | 9.0 | 190 | 77 | 540 | 350 | 14 | 7.0 | 3.7 | 20 | 190 | 20 | 92 | 120 | 49 |
| M-32 | 3.7 | 64 | 70 | 520 | 5.2 | 12 | 6.9 | 100 | 47 | 450 | 270 | 8.4 | 4.5 | 2.0 | 12 | 110 | 11 | 52 | 43 | 29 |
| M-33 | 4.2 | 66 | 83 | 570 | 8.0 | 14 | 6.1 | 130 | 57 | 470 | 280 | 9.2 | 4.5 | 2.6 | 14 | 105 | 14 | 66 | 44 | 34 |
| M-34 | 4.6 | 140 | 210 | 1000 | 11 | 18 | 5.6 | 230 | 94 | 680 | 580 | 16 | 8.7 | 4.5 | 27 | 215 | 23 | 120 | 130 | 52 |
| M-35 | 5.8 | 120 | 110 | 910 | 9.5 | 17 | 23 | 190 | 74 | 560 | 480 | 14 | 6.7 | 4.1 | 19 | 180 | 20 | 96 | 100 | 45 |
| M-36 | 3.7 | 110 | 110 | 810 | 8.2 | 16 | 6.0 | 160 | 65 | 540 | 275 | 12 | 6.2 | 3.9 | 17 | 170 | 18 | 83 | 96 | 42 |
| M-37 | 3.2 | 150 | 120 | 1100 | 8.3 | 16 | 6.4 | 200 | 81 | 640 | 550 | 15 | 7.3 | 4.3 | 21 | 220 | 21 | 100 | 140 | 47 |
| M-38 | 3.1 | 110 | 190 | 1100 | 6.4 | 17 | 7.0 | 160 | 63 | 550 | 260 | 12 | 5.6 | 3.7 | 26 | 230 | 16 | 83 | 69 | 39 |
| M-39 | 2.7 | 96 | 105 | 800 | 6.3 | 16 | 8.9 | 140 | 61 | 540 | 240 | 10 | 5.6 | 3.0 | 18 | 160 | 15 | 71 | 79 | 35 |
| M-40 | 2.9 | 110 | 110 | 860 | 7.0 | 15 | 5.6 | 160 | 56 | 520 | 260 | 11 | 5.7 | 3.4 | 18 | 190 | 18 | 84 | 180 | 36 |
| T-01 | nd | 20 | 85 | 310 | nd | nd | nd | 7.1 | 3.8 | 140 | 55 | 0.65 | 0.50 | nd | 0.50 | 56 | 0.75 | 3.7 | nd | 4.0 |
| T-02 | nd | 14 | 6.0 | 78 | nd | nd | nd | 22 | 7.9 | 150 | 48 | 1.4 | 0.77 | nd | 2.1 | 17 | 2.0 | 9.4 | nd | 7.0 |
| T-03 | nd | 140 | 89 | 860 | nd | nd | nd | 100 | 110 | 590 | 640 | 9.9 | 5.5 | nd | 14 | 180 | 12 | 49 | nd | 63 |
| T-04 | nd | 11 | 30 | 74 | nd | nd | nd | 14 | 7.2 | 150 | 49 | 1.4 | 0.53 | nd | 2.4 | 10 | 1.7 | 7.6 | nd | 16 |
| T-05 | nd | 40 | 61 | 170 | nd | nd | nd | 55 | 16 | 340 | 100 | 3.8 | 1.4 | nd | 5.1 | 37 | 6.9 | 31 | nd | 13 |
| T-06 | nd | 73 | 4.5 | 300 | nd | nd | nd | 110 | 27 | 320 | 94 | 5.7 | 2.5 | nd | 5.7 | 63 | 11 | 59 | nd | 23 |
| T-07 | nd | 52 | 24 | 640 | nd | nd | nd | 110 | 33 | 355 | 160 | 7.1 | 3.2 | nd | 7.0 | 110 | 9.9 | 51 | nd | 23 |
| T-08 | nd | 50 | 21 | 350 | nd | nd | nd | 110 | 33 | 350 | 140 | 6.7 | 3.9 | nd | 8.0 | 71 | 9.6 | 59 | nd | 21 |
| T-09 | nd | 12 | 7.1 | 10 | nd | nd | nd | 11 | 20 | 310 | 85 | 0.82 | 0.79 | nd | 2.9 | 7.0 | 1.3 | 6.1 | nd | 31 |
| T-10 | nd | 25 | 3.5 | 44 | nd | nd | nd | 11 | 16 | 370 | 62 | 1.5 | 1.0 | nd | 1.8 | 9.4 | 2.0 | 5.4 | nd | 23 |
| T-11 | nd | 41 | 24 | 640 | nd | nd | nd | 35 | 9.3 | 330 | 230 | 3.0 | 1.2 | nd | 2.4 | 120 | 4.4 | 18 | nd | 11 |
| T-12 | nd | 13 | 5.9 | 77 | nd | nd | nd | 13 | 9.1 | 300 | 190 | 1.1 | 1.1 | nd | 6.9 | 17 | 1.9 | 7.3 | nd | 8.7 |
| T-13 | nd | 53 | 37 | 360 | nd | nd | nd | 63 | 29 | 430 | 130 | 4.4 | 2.0 | nd | 5.8 | 74 | 6.3 | 31 | nd | 18 |
| T-14 | nd | 87 | 70 | 390 | nd | nd | nd | 90 | 58 | 800 | 280 | 7.8 | 3.3 | nd | 7.3 | 95 | 9.3 | 46 | nd | 58 |
| T-15 | nd | 39 | 45 | 165 | nd | nd | nd | 33 | 19 | 300 | 98 | 2.9 | 1.7 | nd | 3.9 | 36 | 3.3 | 16 | nd | 30 |
| T-16 | nd | 23 | 1.0 | 10 | nd | nd | nd | 6.1 | 2.7 | 250 | 140 | 1.0 | 1.0 | nd | 0.75 | 5.0 | 1.3 | 3.6 | nd | 4.4 |
| T-17 | nd | 53 | 17 | 200 | nd | nd | nd | 64 | 25 | 390 | 140 | 4.0 | 1.9 | nd | 5.8 | 48 | 5.8 | 31 | nd | 29 |
| T-18 | nd | 31 | 40 | 270 | nd | nd | nd | 55 | 16 | 340 | 260 | 5.0 | 3.3 | nd | 10 | 61 | 7.5 | 34 | nd | 18 |
| T-19 | nd | 63 | 63 | 380 | nd | nd | nd | 90 | 23 | 450 | 140 | 8.4 | 4.8 | nd | 17 | 79 | 11 | 51 | nd | 20 |
| T-20 | nd | 52 | 39 | 300 | nd | nd | nd | 92 | 22 | 430 | 100 | 6.9 | 3.9 | nd | 18 | 62 | 9.0 | 50 | nd | 15 |
| T-21 | nd | 300 | 150 | 1700 | nd | nd | nd | 360 | 110 | 790 | 440 | 26 | 13 | nd | 32 | 340 | 38 | 190 | nd | 73 |
| T-22 | nd | 70 | 56 | 1300 | nd | nd | nd | 86 | 22 | 390 | 750 | 9.0 | 5.5 | nd | 28 | 235 | 12 | 120 | nd | 36 |
| T-23 | nd | 110 | 55 | 190 | nd | nd | nd | 28 | 7.3 | 500 | 96 | 2.4 | 1.2 | nd | 10 | 42 | 3.2 | 18 | nd | 18 |
| T-24 | nd | 190 | 410 | 2100 | nd | nd | nd | 260 | 55 | 650 | 235 | 21 | 11 | nd | 92 | 400 | 27 | 140 | nd | 48 |
| T-25 | nd | 390 | 260 | 3100 | nd | nd | nd | 500 | 140 | 880 | 480 | 37 | 21 | nd | 64 | 630 | 53 | 260 | nd | 72 |

*nd – not determined, LOD levels are expressed in mg or µg on filter*

**Table S5:** continued

|  | Mn | Na | Nd | Ni | Pb | Pr | Rb | Sm | Sr | Ti | U | V | Y | Yb | Zn | Zr | Eu/Eu* | Ce/Ce* | Gd/Gd* |
| --- | --- | --- | --- | --- | --- | --- | --- | --- | --- | --- | --- | --- | --- | --- | --- | --- | --- | --- | --- |
| LOD | 0.005 | 0.05 | 1 | 50 | 1 | 1 | 1 | 1 | 5 | 0.01 | 0.5 | 5 | 1 | 1 | 10 | 1 |  |  |  |
|  | mg/L | mg/L | µg/L | µg/L | µg/L | µg/L | µg/L | µg/L | µg/L | mg/L | µg/L | µg/L | µg/L | µg/L | µg/L | µg/L |  |  |  |
| M-00 | 0.03 | 12 | 2.7 | 36 | 22 | 1.0 | 11 | 0.73 | 195 | 0.08 | 1.4 | 3.6 | 20 | 0.73 | 57 | 32 | 4.1 | 0.40 | 0.12 |
| M-01 | 0.05 | 13 | 0.85 | 36 | 24 | 0.73 | 6.3 | 0.73 | 290 | 0.10 | 1.0 | 3.6 | 0.73 | 0.73 | 99 | 0.73 | 2.3 | 0.17 | 0.08 |
| M-02 | 0.37 | 14 | 13 | 82 | 84 | 3.1 | 49 | 2.4 | 280 | 0.84 | 6.4 | 48 | 11 | 1.7 | 130 | 0.73 | 1.5 | 1.1 | 0.71 |
| M-03 | 0.04 | 13 | 1.8 | 29 | 22 | 0.71 | 9.7 | 0.65 | 380 | 0.15 | 1.9 | 11 | 0.57 | 0.57 | 29 | 0.57 | 3.1 | 0.54 | 0.13 |
| M-04 | 0.10 | 11 | 24 | 80 | 43 | 6.1 | 52 | 3.4 | 240 | 0.94 | 2.3 | 58 | 9.2 | 1.7 | 200 | 3.2 | 1.3 | 0.95 | 1.5 |
| M-05 | 0.10 | 11 | 11 | 29 | 34 | 3.6 | 26 | 2.2 | 245 | 0.42 | 1.7 | 34 | 2.4 | 0.90 | 210 | 0.57 | 1.4 | 0.64 | 0.94 |
| M-06 | 0.11 | 12 | 12 | 59 | 40 | 2.7 | 24 | 2.8 | 310 | 0.40 | 2.1 | 31 | 5.0 | 0.72 | 200 | 0.57 | 0.93 | 1.2 | 0.89 |
| M-07 | 0.09 | 10 | 12 | 67 | 35 | 2.7 | 25 | 1.8 | 260 | 0.34 | 1.5 | 25 | 3.0 | 0.92 | 165 | 0.57 | 1.7 | 1.1 | 0.91 |
| M-08 | 0.10 | 17 | 20 | 61 | 45 | 3.3 | 29 | 2.9 | 230 | 0.39 | 1.6 | 33 | 3.5 | 0.88 | 2150 | 3.0 | 1.2 | 1.5 | 1.0 |
| M-09 | 0.11 | 19 | 27 | 75 | 44 | 4.6 | 37 | 3.9 | 220 | 0.63 | 2.0 | 37 | 4.9 | 1.1 | 2500 | 0.57 | 0.86 | 1.2 | 1.8 |
| M-10 | 0.10 | 25 | 23 | 61 | 48 | 3.5 | 28 | 2.0 | 230 | 0.41 | 2.2 | 29 | 24 | 1.0 | 2900 | 22 | 2.1 | 1.4 | 1.6 |
| M-11 | 0.11 | 23 | 34 | 73 | 46 | 5.8 | 33 | 3.8 | 200 | 0.65 | 1.8 | 38 | 14 | 1.1 | 3200 | 1.1 | 1.2 | 1.4 | 1.5 |
| M-12 | 0.08 | 20 | 29 | 75 | 43 | 4.7 | 32 | 3.3 | 170 | 0.62 | 1.9 | 35 | 15 | 0.90 | 3500 | 0.57 | 1.4 | 1.6 | 1.5 |
| M-13 | 0.21 | 15 | 52 | 150 | 81 | 13 | 115 | 8.2 | 270 | 2.6 | 5.5 | 130 | 32 | 3.0 | 770 | 5.4 | 1.3 | 1.1 | 1.3 |
| M-14 | 0.11 | 15 | 15 | 29 | 43 | 3.9 | 29 | 3.5 | 180 | 0.72 | 1.6 | 38 | 7.0 | 0.92 | 800 | 0.57 | 0.87 | 1.0 | 0.83 |
| M-15 | 0.09 | 15 | 16 | 29 | 38 | 3.0 | 29 | 2.6 | 250 | 0.52 | 1.7 | 32 | 4.4 | 0.79 | 620 | 0.57 | 1.2 | 1.4 | 1.0 |
| M-16 | 1.3 | 16 | 30 | 145 | 450 | 7.4 | 58 | 5.0 | 270 | 1.2 | 3.2 | 78 | 16 | 1.9 | 3600 | 7.5 | 1.4 | 1.0 | 0.84 |
| M-17 | 0.51 | 14 | 16 | 59 | 160 | 3.5 | 30 | 3.1 | 220 | 0.65 | 2.2 | 37 | 5.6 | 0.70 | 1350 | 0.57 | 1.1 | 1.2 | 0.82 |
| M-18 | 0.66 | 15 | 17 | 92 | 225 | 3.7 | 28 | 3.2 | 210 | 0.71 | 1.8 | 43 | 6.9 | 0.81 | 1800 | 4.4 | 0.88 | 1.2 | 1.3 |
| M-19 | 0.76 | 15 | 28 | 580 | 260 | 6.3 | 56 | 5.2 | 300 | 1.1 | 2.8 | 99 | 13 | 1.5 | 2100 | 7.1 | 1.0 | 1.2 | 1.2 |
| M-20 | 0.60 | 14 | 29 | 240 | 220 | 6.9 | 49 | 5.3 | 260 | 0.92 | 2.9 | 71 | 30 | 1.8 | 1900 | 80 | 1.5 | 1.1 | 0.71 |
| M-21 | 0.59 | 14 | 18 | 180 | 220 | 3.7 | 35 | 3.6 | 205 | 0.60 | 1.9 | 52 | 8.4 | 1.2 | 1500 | 160 | 1.0 | 1.4 | 1.0 |
| M-22 | 0.96 | 13 | 26 | 310 | 270 | 6.4 | 47 | 4.1 | 260 | 0.94 | 2.3 | 76 | 18 | 1.8 | 2550 | 4.4 | 1.4 | 1.0 | 1.2 |
| M-23 | 1.3 | 19 | 87 | 390 | 470 | 21 | 190 | 18 | 510 | 4.3 | 7.8 | 260 | 64 | 5.6 | 2200 | 20 | 1.1 | 1.2 | 0.89 |
| M-24 | 0.79 | 13 | 26 | 170 | 230 | 7.2 | 63 | 5.7 | 260 | 1.1 | 2.6 | 80 | 19 | 2.2 | 1500 | 5.0 | 0.89 | 0.93 | 1.2 |
| M-25 | 2.8 | 36 | 245 | 670 | 1400 | 63 | 430 | 50 | 1100 | 8.5 | 18 | 530 | 210 | 18 | 3000 | 66 | 1.1 | 1.1 | 1.1 |
| M-26 | 0.69 | 10 | 75 | 155 | 170 | 20 | 180 | 17 | 290 | 3.0 | 5.4 | 210 | 55 | 4.9 | 710 | 12 | 1.0 | 1.1 | 1.0 |
| M-27 | 0.87 | 17 | 110 | 320 | 355 | 27 | 220 | 22 | 450 | 3.7 | 6.9 | 260 | 86 | 7.9 | 1500 | 25 | 1.1 | 1.1 | 1.2 |
| M-28 | 0.55 | 17 | 67 | 215 | 250 | 16 | 140 | 14 | 360 | 2.8 | 4.3 | 190 | 52 | 4.1 | 910 | 16 | 0.95 | 1.1 | 1.2 |
| M-29 | 0.63 | 13 | 75 | 250 | 230 | 17 | 140 | 14 | 350 | 2.6 | 4.8 | 180 | 50 | 5.5 | 1100 | 18 | 1.1 | 1.3 | 1.2 |
| M-30 | 0.62 | 15 | 80 | 250 | 240 | 21 | 180 | 16 | 380 | 3.5 | 5.7 | 240 | 72 | 6.4 | 1100 | 1900 | 1.2 | 1.0 | 1.0 |
| M-31 | 0.67 | 18 | 87 | 250 | 270 | 21 | 190 | 16 | 480 | 3.1 | 5.3 | 340 | 65 | 6.5 | 1200 | 52 | 1.1 | 1.2 | 1.2 |
| M-32 | 0.43 | 11 | 52 | 1500 | 180 | 12 | 100 | 10 | 290 | 1.7 | 3.1 | 140 | 37 | 4.2 | 1100 | 17 | 0.94 | 1.1 | 1.3 |
| M-33 | 0.55 | 13 | 62 | 200 | 200 | 14 | 110 | 11 | 310 | 2.0 | 3.7 | 160 | 44 | 4.3 | 1100 | 13 | 1.2 | 1.2 | 1.2 |
| M-34 | 0.86 | 14 | 110 | 360 | 310 | 27 | 210 | 21 | 430 | 4.2 | 6.4 | 290 | 83 | 7.7 | 1500 | 45 | 1.1 | 1.1 | 1.2 |
| M-35 | 0.68 | 12 | 91 | 260 | 250 | 22 | 180 | 14 | 380 | 3.6 | 5.1 | 250 | 65 | 5.5 | 1350 | 19 | 1.6 | 1.1 | 1.1 |
| M-36 | 0.62 | 12 | 73 | 245 | 220 | 20 | 170 | 14 | 370 | 3.1 | 4.8 | 220 | 55 | 5.6 | 890 | 16 | 1.4 | 1.0 | 0.92 |
| M-37 | 0.72 | 13 | 95 | 240 | 240 | 23 | 230 | 19 | 420 | 4.4 | 5.3 | 290 | 68 | 6.3 | 1200 | 22 | 1.0 | 1.1 | 1.0 |
| M-38 | 0.58 | 12 | 76 | 235 | 180 | 19 | 170 | 15 | 480 | 3.4 | 4.5 | 240 | 55 | 5.9 | 810 | 16 | 1.2 | 1.0 | 0.94 |
| M-39 | 0.51 | 11 | 63 | 170 | 180 | 16 | 150 | 14 | 360 | 2.9 | 3.8 | 210 | 45 | 5.0 | 860 | 10 | 1.0 | 1.1 | 1.1 |
| M-40 | 0.53 | 12 | 78 | 180 | 180 | 19 | 160 | 14 | 360 | 3.1 | 5.5 | 220 | 88 | 5.2 | 790 | 82 | 1.1 | 1.1 | 1.2 |
| T-01 | 0.02 | nd | 4.3 | nd | 7.0 | 0.91 | 7.4 | 0.94 | 72 | 0.11 | 2.2 | 2.5 | 62 | 0.50 | nd | nd | nd | 1.2 | nd |
| T-02 | 0.06 | nd | 8.9 | nd | 17 | 2.6 | 15 | 2.1 | 85 | 0.45 | 1.5 | 26 | 3.5 | 1.0 | nd | nd | nd | 0.90 | nd |
| T-03 | 0.59 | nd | 52 | nd | 200 | 12 | 250 | 11 | 590 | 1.7 | 4.2 | 220 | 41 | 4.5 | nd | nd | nd | 1.2 | nd |
| T-04 | 0.06 | nd | 7.3 | nd | 20 | 1.9 | 20 | 1.4 | 100 | 0.44 | 1.3 | 28 | 1.2 | 0.6 | nd | nd | nd | 0.95 | nd |
| T-05 | 0.07 | nd | 27 | nd | 22 | 6.9 | 78 | 6.3 | 140 | 1.3 | 3.2 | 83 | 5.4 | 1.5 | nd | nd | nd | 1.0 | nd |
| T-06 | 0.09 | nd | 55 | nd | 230 | 15 | 120 | 9.2 | 160 | 2.5 | 2.8 | 120 | 17 | 1.9 | nd | nd | nd | 0.95 | nd |
| T-07 | 0.23 | nd | 47 | nd | 130 | 13 | 110 | 10 | 240 | 2.7 | 5.6 | 130 | 21 | 2.4 | nd | nd | nd | 1.0 | nd |
| T-08 | 0.22 | nd | 52 | nd | 56 | 13 | 95 | 10 | 160 | 2.6 | 8.0 | 120 | 27 | 3.9 | nd | nd | nd | 1.1 | nd |
| T-09 | 0.04 | nd | 5.3 | nd | 12 | 1.7 | 9.0 | 1.2 | 21 | 0.35 | 0.25 | 25 | 0.50 | 0.50 | nd | nd | nd | 0.68 | nd |
| T-10 | 0.05 | nd | 5.3 | nd | 10 | 1.5 | 5.9 | 2.0 | 40 | 0.36 | 0.50 | 25 | 1.0 | 1.0 | nd | nd | nd | 0.86 | nd |
| T-11 | 0.15 | nd | 18 | nd | 1000 | 4.5 | 18 | 3.1 | 420 | 0.34 | 6.5 | 31 | 120 | 1.0 | nd | nd | nd | 1.0 | nd |
| T-12 | 0.08 | nd | 7.3 | nd | 120 | 2.0 | 12 | 2.2 | 79 | 0.64 | 1.0 | 34 | 1.0 | 1.0 | nd | nd | nd | 0.74 | nd |
| T-13 | 0.41 | nd | 28 | nd | 93 | 7.5 | 94 | 6.9 | 190 | 0.89 | 2.9 | 99 | 14 | 1.9 | nd | nd | nd | 1.0 | nd |
| T-14 | 0.27 | nd | 46 | nd | 950 | 10 | 160 | 11 | 470 | 1.4 | 4.2 | 160 | 29 | 3.0 | nd | nd | nd | 1.2 | nd |
| T-15 | 0.11 | nd | 13 | nd | 64 | 3.5 | 30 | 2.7 | 250 | 0.96 | 1.7 | 64 | 4.7 | 1.1 | nd | nd | nd | 1.1 | nd |
| T-16 | 0.02 | nd | 3.6 | nd | 115 | 1.1 | 1.8 | 1.0 | 52 | 0.26 | 0.50 | 10 | 1.0 | 1.0 | nd | nd | nd | 0.62 | nd |
| T-17 | 0.16 | nd | 28 | nd | 150 | 6.9 | 42 | 4.7 | 140 | 1.8 | 1.2 | 105 | 8.0 | 1.4 | nd | nd | nd | 1.2 | nd |
| T-18 | 0.21 | nd | 29 | nd | 140 | 7.6 | 45 | 6.9 | 240 | 0.76 | 1.8 | 75 | 22 | 2.2 | nd | nd | nd | 0.87 | nd |
| T-19 | 0.16 | nd | 48 | nd | 84 | 11 | 76 | 9.0 | 220 | 1.8 | 2.0 | 130 | 31 | 3.9 | nd | nd | nd | 1.2 | nd |
| T-20 | 0.19 | nd | 48 | nd | 55 | 11 | 84 | 10 | 230 | 1.6 | 2.6 | 145 | 31 | 2.8 | nd | nd | nd | 1.1 | nd |
| T-21 | 0.63 | nd | 170 | nd | 280 | 44 | 440 | 33 | 640 | 8.7 | 9.8 | 500 | 160 | 11 | nd | nd | nd | 1.0 | nd |
| T-22 | 0.07 | nd | 47 | nd | 53 | 10 | 190 | 9.7 | 950 | 1.6 | 3.0 | 110 | 51 | 4.7 | nd | nd | nd | 1.3 | nd |
| T-23 | 0.06 | nd | 16 | nd | 62 | 3.6 | 26 | 3.1 | 190 | 0.71 | 1.7 | 51 | 3.5 | 1.3 | nd | nd | nd | 1.1 | nd |
| T-24 | 0.51 | nd | 120 | nd | 130 | 32 | 280 | 25 | 580 | 5.7 | 6.0 | 490 | 100 | 9.4 | nd | nd | nd | 1.0 | nd |
| T-25 | 2.0 | nd | 240 | nd | 250 | 60 | 590 | 46 | 810 | 11 | 12 | 680 | 190 | 17 | nd | nd | nd | 1.1 | nd |

*nd – not determined, LOD levels are expressed in mg or µg on filter*

**Table S6:** Element levels of 32 elements in SPM samples at average water regime (main channel)

|  | Al | As | Ba | Ca | Ce | Co | Cr | Dy | Er | Eu | Fe | Ga | Gd | La | Li | Mg | Mn |
| --- | --- | --- | --- | --- | --- | --- | --- | --- | --- | --- | --- | --- | --- | --- | --- | --- | --- |
| LOD | 1 | 1 | 0.01 | 5 | 1 | 1 | 0.01 | 1 | 1 | 1 | 0.05 | 5 | 1 | 1 | 5 | 1 | 0.005 |
| unit | mg/L | μg/L | mg/L | mg/L | μg/L | μg/L | mg/L | μg/L | μg/L | μg/L | mg/L | μg/L | μg/L | μg/L | μg/L | mg/L | mg/L |
| M-00 | 26 | 3.2 | 0.44 | 100 | 8.7 | 2.0 | 0.70 | 2.0 | 2.0 | 2.0 | 14 | 15 | 2.0 | 4.1 | 45 | 8.7 | 0.69 |
| M-01 | 17 | 19 | 0.09 | 170 | 11 | 2.0 | 0.48 | 2.0 | 2.0 | 2.0 | 18 | 10 | 2.7 | 4.1 | 52 | 11 | 1.6 |
| M-02 | 29 | 220 | 0.24 | 245 | 18 | 2.0 | 0.49 | 2.7 | 2.0 | 2.0 | 45 | 10 | 4.0 | 6.8 | 65 | 18 | 1.7 |
| M-03 | 360 | 370 | 2.0 | 1300 | 400 | 310 | 1.5 | 32 | 16 | 8.0 | 450 | 110 | 40 | 175 | 490 | 340 | 21 |
| M-04 | 66 | 66 | 0.35 | 250 | 58 | 14 | 0.59 | 4.0 | 2.0 | 2.0 | 51 | 10 | 8.0 | 31 | 93 | 35 | 1.7 |
| M-05 | 120 | 180 | 0.74 | 320 | 100 | 50 | 0.66 | 8.0 | 4.0 | 4.0 | 100 | 45 | 8.0 | 51 | 140 | 62 | 5.1 |
| M-06 | 560 | 550 | 3.0 | 790 | 570 | 320 | 1.7 | 40 | 20 | 12 | 495 | 190 | 48 | 260 | 585 | 310 | 22 |
| M-07 | 240 | 200 | 1.6 | 360 | 270 | 100 | 1.06 | 16 | 8.0 | 4.0 | 250 | 81 | 20 | 120 | 250 | 110 | 6.6 |
| M-08 | 56 | 10 | 0.29 | 220 | 50 | 10 | 0.60 | 4.0 | 2.0 | 2.0 | 44 | 10 | 4.0 | 27 | 97 | 28 | 1.4 |
| M-09 | 150 | 170 | 1.1 | 330 | 170 | 62 | 1.0 | 12 | 8.0 | 4.0 | 140 | 57 | 16 | 83 | 200 | 67 | 4.9 |
| M-10 | 110 | 130 | 0.87 | 300 | 130 | 46 | 0.80 | 8.0 | 4.0 | 4.0 | 110 | 81 | 12 | 59 | 130 | 58 | 3.4 |
| M-11 | 140 | 140 | 0.89 | 300 | 190 | 66 | 0.77 | 12 | 4.0 | 4.0 | 140 | 45 | 12 | 91 | 160 | 68 | 4.6 |
| M-12 | 150 | 140 | 1.0 | 305 | 230 | 82 | 1.1 | 16 | 4.0 | 4.0 | 170 | 53 | 16 | 99 | 150 | 60 | 4.4 |
| M-13 | 110 | 94 | 0.79 | 360 | 160 | 50 | 0.72 | 8.0 | 4.0 | 4.0 | 110 | 37 | 12 | 75 | 160 | 55 | 4.6 |
| M-14 | 150 | 110 | 1.0 | 260 | 190 | 54 | 0.81 | 12 | 4.0 | 4.0 | 120 | 69 | 12 | 79 | 160 | 57 | 4.8 |
| M-15 | 130 | 100 | 1.0 | 280 | 190 | 50 | 0.81 | 8 | 4.0 | 4.0 | 110 | 53 | 12 | 83 | 145 | 51 | 4.4 |
| M-16 | 520 | 390 | 3.3 | 420 | 710 | 250 | 1.5 | 44 | 20 | 12 | 460 | 220 | 52 | 330 | 565 | 190 | 15 |
| M-17 | 370 | 260 | 2.3 | 410 | 490 | 180 | 1.2 | 28 | 12 | 8.0 | 320 | 140 | 36 | 220 | 465 | 140 | 17 |
| M-18 | 320 | 270 | 2.1 | 370 | 480 | 170 | 1.1 | 24 | 12 | 8.0 | 310 | 160 | 32 | 210 | 370 | 130 | 16 |
| M-19 | 250 | 220 | 1.7 | 370 | 390 | 140 | 1.0 | 24 | 8.0 | 8.0 | 260 | 93 | 28 | 180 | 320 | 110 | 14 |
| M-20 | 320 | 290 | 2.1 | 370 | 530 | 170 | 1.1 | 28 | 12 | 8.0 | 340 | 120 | 40 | 230 | 380 | 135 | 15 |
| M-21 | 265 | 250 | 1.8 | 370 | 480 | 170 | 1.2 | 28 | 12 | 8.0 | 320 | 97 | 36 | 210 | 380 | 130 | 15 |
| M-22 | 290 | 270 | 2.6 | 370 | 500 | 170 | 1.2 | 28 | 12 | 8.0 | 360 | 125 | 32 | 215 | 370 | 130 | 16 |
| M-23 | 300 | 290 | 2.3 | 390 | 510 | 170 | 1.1 | 28 | 12 | 8.0 | 250 | 120 | 36 | 220 | 370 | 140 | 18 |
| M-24 | 260 | 220 | 1.8 | 300 | 340 | 110 | 0.99 | 16 | 8.0 | 8.0 | 230 | 100 | 28 | 150 | 220 | 88 | 13 |
| M-25 | 620 | 440 | 3.9 | 450 | 910 | 290 | 1.8 | 48 | 20 | 16 | 550 | 240 | 72 | 390 | 530 | 205 | 25 |
| M-26 | 1500 | 960 | 9.0 | 390 | 2000 | 620 | 2.7 | 96 | 44 | 32 | 1200 | 580 | 140 | 855 | 1150 | 380 | 34 |
| M-27 | 1000 | 670 | 6.1 | 330 | 1400 | 430 | 2.0 | 76 | 32 | 24 | 815 | 380 | 100 | 610 | 825 | 265 | 25 |
| M-28 | 480 | 330 | 2.8 | 335 | 690 | 220 | 1.3 | 40 | 12 | 12 | 420 | 170 | 52 | 295 | 400 | 150 | 16 |
| M-29 | 340 | 260 | 2.1 | 250 | 550 | 150 | 1.1 | 28 | 12 | 8.0 | 330 | 120 | 40 | 235 | 320 | 110 | 15 |
| M-30 | 610 | 360 | 3.5 | 415 | 990 | 300 | 1.7 | 56 | 24 | 16 | 585 | 230 | 68 | 420 | 585 | 220 | 29 |
| M-31 | 450 | 360 | 2.6 | 350 | 820 | 270 | 1.5 | 40 | 20 | 12 | 520 | 165 | 60 | 355 | 465 | 180 | 26 |
| M-32 | 440 | 330 | 2.7 | 440 | 730 | 230 | 1.5 | 44 | 16 | 12 | 460 | 160 | 52 | 315 | 440 | 165 | 24 |
| M-33 | 310 | 250 | 1.8 | 210 | 540 | 170 | 1.0 | 28 | 16 | 8.0 | 340 | 110 | 40 | 230 | 280 | 120 | 19 |
| M-34 | 410 | 260 | 2.5 | 330 | 590 | 190 | 1.5 | 32 | 16 | 8.0 | 380 | 150 | 40 | 255 | 345 | 130 | 21 |
| M-35 | 580 | 350 | 3.4 | 370 | 830 | 270 | 1.5 | 48 | 20 | 16 | 530 | 210 | 60 | 355 | 540 | 190 | 25 |
| M-36 | 320 | 190 | 1.9 | 350 | 470 | 140 | 1.1 | 24 | 12 | 8.0 | 290 | 110 | 32 | 200 | 330 | 110 | 16 |
| M-37 | 280 | 170 | 1.8 | 260 | 390 | 110 | 1.0 | 20 | 8.0 | 8.0 | 235 | 100 | 24 | 170 | 265 | 86 | 14 |
| M-38 | 230 | 160 | 1.6 | 320 | 370 | 100 | 0.94 | 16 | 8.0 | 4.0 | 230 | 89 | 24 | 160 | 265 | 79 | 17 |
| M-39 | 180 | 140 | 1.2 | 330 | 300 | 86 | 1.1 | 16 | 8.0 | 4.0 | 240 | 61 | 24 | 130 | 165 | 65 | 13 |
| M-40 | 160 | 160 | 1.1 | 260 | 330 | 90 | 0.80 | 16 | 8.0 | 8.0 | 195 | 57 | 24 | 140 | 160 | 68 | 13 |

*nd – not determined, LOD levels are expressed in mg or µg on filter*

**Table S6:** continued

|  | Na | Nd | Ni | Pb | Pr | Rb | Sc | Sm | Sr | Th | Ti | U | V | Y | Yb | Eu/Eu* | Ce/Ce* | Gd/Gd* |
| --- | --- | --- | --- | --- | --- | --- | --- | --- | --- | --- | --- | --- | --- | --- | --- | --- | --- | --- |
|  | 0.05 | 1 | 0.05 | 0.001 | 1 | 0.001 | 5 | 1 | 0.005 | 5 | 0.01 | 0.5 | 0.005 | 1 | 1 |  |  |  |
|  | mg/L | μg/L | mg/L | mg/L | μg/L | mg/L | μg/L | μg/L | mg/L | μg/L | mg/L | μg/L | mg/L | μg/L | μg/L |  |  |  |
| M-00 | 32 | 5.3 | 0.03 | 0.10 | 2.7 | 0.03 | 1.8 | 2.0 | 0.20 | 5.9 | 0.42 | 4.0 | 0.01 | 8.0 | 2.0 | 3.5 | 0.21 | 0.11 |
| M-01 | 31 | 8.0 | 0.02 | 0.02 | 2.0 | 0.03 | 1.8 | 2.7 | 0.26 | 2.8 | 0.48 | 4.0 | 0.01 | 9.3 | 2.0 | 2.8 | 0.73 | 0.17 |
| M-02 | 32 | 12 | 0.28 | 0.04 | 4.0 | 0.03 | 1.8 | 4.0 | 0.39 | 3.2 | 1.0 | 4.0 | 0.03 | 15 | 2.0 | 1.8 | 0.43 | 0.28 |
| M-03 | 43 | 180 | 1.3 | 0.26 | 44 | 0.42 | 44 | 36 | 2.7 | 87 | 5.9 | 12 | 0.44 | 160 | 16 | 1.1 | 1.2 | 1.1 |
| M-04 | 37 | 32 | 0.12 | 0.04 | 8.0 | 0.08 | 1.8 | 8.0 | 0.46 | 15 | 1.5 | 4.0 | 0.07 | 24 | 2.0 | 1.1 | 0.93 | 0.79 |
| M-05 | 39 | 48 | 0.20 | 0.08 | 12 | 0.14 | 12 | 8.0 | 0.63 | 15 | 2.7 | 4.0 | 0.17 | 48 | 4.0 | 2.6 | 1.1 | 0.36 |
| M-06 | 35 | 260 | 1.1 | 0.35 | 64 | 0.58 | 92 | 48 | 1.9 | 91 | 15 | 20 | 0.79 | 200 | 20 | 1.2 | 1.2 | 0.88 |
| M-07 | 32 | 120 | 0.35 | 0.17 | 32 | 0.30 | 20 | 20 | 0.92 | 43 | 6.6 | 12 | 0.33 | 88 | 8.0 | 1.1 | 1.0 | 1.2 |
| M-08 | 21 | 28 | 0.05 | 0.02 | 8 | 0.06 | 1.8 | 4.0 | 0.42 | 11 | 1.5 | 4.0 | 0.06 | 20 | 2.0 | 2.8 | 0.70 | 0.38 |
| M-09 | 29 | 80 | 0.21 | 0.17 | 20 | 0.20 | 16 | 20 | 0.66 | 23 | 3.3 | 8.0 | 0.21 | 60 | 4.0 | 0.85 | 1.1 | 0.85 |
| M-10 | 28 | 60 | 0.17 | 0.09 | 16 | 0.14 | 20 | 12 | 0.61 | 19 | 3.1 | 4.0 | 0.21 | 44 | 4.0 | 1.6 | 1.0 | 0.58 |
| M-11 | 27 | 92 | 0.20 | 0.14 | 20 | 0.17 | 24 | 20 | 0.62 | 31 | 3.5 | 8.0 | 0.19 | 60 | 4.0 | 0.91 | 1.4 | 0.67 |
| M-12 | 27 | 100 | 0.25 | 0.12 | 24 | 0.20 | 24 | 24 | 0.56 | 31 | 4.1 | 8.0 | 0.27 | 60 | 8.0 | 0.72 | 1.3 | 0.91 |
| M-13 | 38 | 80 | 0.16 | 0.12 | 20 | 0.15 | 3.6 | 12 | 0.59 | 27 | 2.7 | 4.0 | 0.17 | 48 | 4.0 | 1.8 | 1.0 | 0.64 |
| M-14 | 36 | 84 | 0.21 | 0.11 | 20 | 0.18 | 12 | 16 | 0.57 | 27 | 3.5 | 8.0 | 0.23 | 52 | 4.0 | 1.2 | 1.3 | 0.65 |
| M-15 | 33 | 80 | 0.20 | 0.13 | 20 | 0.17 | 12 | 12 | 0.52 | 27 | 3.3 | 8.0 | 0.19 | 52 | 4.0 | 1.8 | 1.2 | 0.64 |
| M-16 | 43 | 320 | 0.90 | 0.42 | 80 | 0.64 | 100 | 64 | 1.1 | 110 | 15 | 24 | 0.83 | 220 | 20 | 0.88 | 1.1 | 1.0 |
| M-17 | 44 | 220 | 0.57 | 0.38 | 56 | 0.48 | 56 | 40 | 0.84 | 83 | 9.9 | 16 | 0.55 | 140 | 12 | 1.0 | 1.1 | 1.1 |
| M-18 | 36 | 200 | 0.55 | 0.41 | 52 | 0.41 | 60 | 36 | 0.75 | 79 | 8.9 | 16 | 0.53 | 140 | 12 | 1.1 | 1.2 | 0.92 |
| M-19 | 38 | 170 | 0.49 | 0.30 | 44 | 0.32 | 36 | 32 | 0.74 | 63 | 7.1 | 12 | 0.41 | 100 | 8.0 | 1.2 | 1.1 | 0.76 |
| M-20 | 33 | 230 | 0.65 | 0.39 | 60 | 0.44 | 48 | 44 | 0.82 | 79 | 9.1 | 16 | 0.51 | 150 | 12 | 0.89 | 1.1 | 1.2 |
| M-21 | 40 | 220 | 0.63 | 0.37 | 56 | 0.36 | 48 | 40 | 0.83 | 75 | 7.8 | 16 | 0.43 | 130 | 12 | 1.0 | 1.1 | 1.1 |
| M-22 | 60 | 220 | 0.68 | 0.43 | 56 | 0.36 | 48 | 44 | 0.82 | 79 | 8.0 | 16 | 0.46 | 140 | 12 | 0.85 | 1.1 | 0.94 |
| M-23 | 41 | 230 | 0.61 | 0.47 | 52 | 0.40 | 52 | 48 | 0.85 | 83 | 8.4 | 16 | 0.48 | 150 | 12 | 0.77 | 1.4 | 1.1 |
| M-24 | 35 | 140 | 0.41 | 0.35 | 40 | 0.34 | 44 | 24 | 0.66 | 59 | 6.5 | 12 | 0.40 | 96 | 8 | 1.7 | 0.93 | 0.70 |
| M-25 | 46 | 390 | 0.97 | 0.71 | 96 | 0.73 | 100 | 84 | 1.1 | 150 | 17 | 28 | 0.98 | 250 | 24 | 0.87 | 1.2 | 1.0 |
| M-26 | 62 | 860 | 1.9 | 1.4 | 220 | 1.8 | 240 | 170 | 1.6 | 360 | 34 | 76 | 2.2 | 490 | 44 | 0.91 | 1.2 | 1.0 |
| M-27 | 53 | 600 | 1.8 | 1.1 | 150 | 1.2 | 170 | 110 | 1.2 | 255 | 24 | 52 | 1.5 | 340 | 28 | 1.1 | 1.2 | 1.0 |
| M-28 | 39 | 290 | 0.75 | 0.54 | 76 | 0.55 | 76 | 64 | 0.82 | 220 | 12 | 24 | 0.73 | 180 | 16 | 0.85 | 1.1 | 1.0 |
| M-29 | 65 | 230 | 0.55 | 0.43 | 56 | 0.42 | 40 | 44 | 0.59 | 87 | 9.0 | 16 | 0.54 | 140 | 12 | 0.88 | 1.3 | 1.2 |
| M-30 | 44 | 410 | 0.97 | 0.71 | 100 | 0.74 | 130 | 84 | 1.0 | 155 | 18 | 32 | 0.97 | 270 | 24 | 0.89 | 1.2 | 1.0 |
| M-31 | 35 | 340 | 0.88 | 0.63 | 88 | 0.57 | 84 | 68 | 0.82 | 135 | 13 | 28 | 0.73 | 210 | 20 | 0.84 | 1.2 | 1.2 |
| M-32 | 40 | 310 | 0.81 | 0.59 | 80 | 0.51 | 64 | 60 | 0.86 | 120 | 11 | 24 | 0.69 | 180 | 20 | 1.0 | 1.1 | 1.0 |
| M-33 | 33 | 220 | 0.59 | 0.45 | 60 | 0.36 | 52 | 48 | 0.61 | 91 | 8.2 | 20 | 0.48 | 150 | 12 | 0.75 | 1.0 | 1.2 |
| M-34 | 37 | 260 | 0.79 | 0.49 | 64 | 0.50 | 60 | 52 | 0.76 | 91 | 11 | 16 | 0.61 | 170 | 16 | 0.72 | 1.2 | 1.2 |
| M-35 | 41 | 350 | 1.2 | 0.61 | 88 | 0.70 | 110 | 64 | 0.99 | 130 | 15 | 24 | 0.92 | 240 | 20 | 1.2 | 1.2 | 0.82 |
| M-36 | 41 | 200 | 0.75 | 0.37 | 48 | 0.40 | 56 | 36 | 0.69 | 79 | 7.8 | 12 | 0.50 | 130 | 12 | 1.1 | 1.3 | 0.91 |
| M-37 | 40 | 160 | 0.51 | 0.32 | 40 | 0.32 | 28 | 28 | 0.61 | 63 | 6.8 | 12 | 0.40 | 100 | 8.0 | 1.4 | 1.2 | 0.63 |
| M-38 | 40 | 160 | 0.47 | 0.31 | 40 | 0.30 | 52 | 36 | 0.62 | 63 | 5.8 | 12 | 0.37 | 100 | 8.0 | 0.49 | 1.2 | 1.6 |
| M-39 | 37 | 130 | 0.32 | 0.25 | 32 | 0.22 | 20 | 24 | 0.55 | 51 | 4.3 | 8.0 | 0.31 | 80 | 8.0 | 0.82 | 1.2 | 1.5 |
| M-40 | 37 | 140 | 0.32 | 0.26 | 36 | 0.22 | 28 | 28 | 0.53 | 51 | 4.1 | 12 | 0.27 | 88 | 8.0 | 1.3 | 1.1 | 0.60 |

*nd – not determined, LOD levels are expressed in mg or µg on filter*
